# Early Suppression of Macrophage Gene Expression by *Leishmania braziliensis*

**DOI:** 10.3389/fmicb.2018.02464

**Published:** 2018-10-15

**Authors:** Rosana Sousa, Viviane M. Andrade, Thomas Bair, Nicholas A. Ettinger, Luana Guimarães, Laura Andrade, Luiz H. Guimarães, Paulo R. L. Machado, Edgar M. Carvalho, Mary E. Wilson, Albert Schriefer

**Affiliations:** ^1^Serviço de Imunologia, Hospital Universitário Professor Edgard Santos, Universidade Federal da Bahia, Salvador, Brazil; ^2^DNA Facility, The University of Iowa, Iowa City, IA, United States; ^3^Deptartment of Pediatrics-Critical Care, Baylor College of Medicine, Houston, TX, United States; ^4^Centro de Formação em Saúde, Universidade Federal do Sul da Bahia, Teixeira de Freitas, Brazil; ^5^Instituto Nacional de Ciência e Tecnologia – Doenças Tropicais, Salvador, Brazil; ^6^Instituto Gonçalo Moniz, FIOCRUZ, Salvador, Brazil; ^7^Departments of Internal Medicine and Microbiology, VA Medical Center, The University of Iowa, Iowa City, IA, United States; ^8^Departamento de Ciências da Biointeração, Instituto de Ciências da Saúde, Universidade Federal da Bahia, Salvador, Brazil

**Keywords:** *Leishmania braziliensis*, strains, macrophage, gene expression, leishmaniasis, cutaneous, muco-cutaneous, disseminated

## Abstract

*Leishmania braziliensis* is an intracellular parasite that resides mostly in macrophages. Both the parasite genome and the clinical disease manifestations show considerable polymorphism. Clinical syndromes caused by *L. braziliensis* include localized cutaneous (CL), mucosal (ML), and disseminated leishmaniasis (DL). Our prior studies showed that genetically distinct *L. braziliensis* clades associate with different clinical types. Herein, we hypothesized that: (1) *L. braziliensis* induces changes in macrophage gene expression that facilitates infection; (2) infection of macrophages with strains associated with CL (clade B), ML (clade C), or DL (clade A) will differentially affect host cell gene expression, reflecting their different pathogenic mechanisms; and (3) differences between the strains will be reflected by differences in macrophage gene expression after initial exposure to the parasite. Human monocyte derived macrophages were infected with *L. braziliensis* isolates from clades A, B, or C. Patterns of gene expression were compared using Affymetrix DNA microarrays. Many transcripts were significantly decreased by infection with all isolates. The most dramatically decreased transcripts encoded proteins involved in signaling pathways, apoptosis, or mitochondrial oxidative phosphorylation. Some transcripts encoding stress response proteins were up-regulated. Differences between *L. braziliensis* clades were observed in the magnitude of change, rather than the identity of transcripts. Isolates from subjects with metastatic disease (ML and DL) induced a greater magnitude of change than isolates from CL. We conclude that *L. braziliensis* enhances its intracellular survival by inhibiting macrophage pathways leading to microbicidal activity. Parasite strains destined for dissemination may exert a more profound suppression than less invasive *L. braziliensis* strains that remain near the cutaneous site of inoculation.

## Introduction

Leishmaniasis refers to a spectrum of human diseases caused by protozoan parasites belonging to the genus *Leishmania*, subgenus either *Leishmania* or *Viannia*. All forms of leishmaniasis are initiated when the promastigote form of the parasite is introduced into the skin of a mammalian host during a sand fly bite, after which they enter local phagocytic cells. Thereafter, parasites convert to their intracellular amastigote form and reside intracellularly. Most parasites reside in host macrophages throughout chronic infection. Amastigotes multiply and spread to new phagocytes, disseminating through host tissues in a pattern dictated primarily by the particular species of the organism. Productive infections may be either asymptomatic, or lead to different clinical syndromes involving internal organs, skin and/or mucosal surfaces (Azulay and Azulay Junior, 1995; Murray et al., 2005).

*Leishmania braziliensis*, a member of the *Leishmania* and *Viannia* sub-genus, is prevalent in Latin America. *L. braziliensis* causes at least three clinical types of tegumentary disease: localized cutaneous (CL), mucosal (ML), and disseminated leishmaniasis (DL) (Costa et al., 1986; Carvalho et al., 1994; Azulay and Azulay Junior, 1995; Bacellar et al., 2002; Turetz et al., 2002; Murray et al., 2005). The most striking feature differentiating CL from either ML or DL is the degree of metastasis of the microorganism, with consequent disease manifestations limited to or distant from the original inoculation site. Localized cutaneous leishmaniasis causes ulcerated lesions restricted to the parasite entry site in the skin, whereas ML is defined by spread of lesions to non-adjacent mucosal surfaces of upper digestive and airways tracts. DL is characterized by parasite dissemination causing lesions throughout skin sites of the infected patient (Machado et al., 2011).

Individuals living in the region of Corte de Pedra in the state of Bahia, Northeast Brazil, can be afflicted with any of the above three phenotypes of *L. braziliensis* disease.

With the current study we want to follow up our prior observation that *L. braziliensis* isolates derived from individuals with different clinical syndromes can be distinguished by polymorphic markers in the *L. braziliensis* genome, and these markers distinguish separate clades of *L. braziliensis* (Schriefer et al., 2004; Queiroz et al., 2012; Guimaraes et al., 2016). We have used this information to track parasite isolates during their movement through time and geographic parts of endemic areas (Schriefer et al., 2004, 2009). The goal of the current study was to discern whether these genotypic differences lead to detectable differences in host macrophage responses. Because the earliest responses seem to be critical in leishmaniasis, we used a model that would be most relevant to initial infection of a naïve host with *L. braziliensis*. Three clades corresponding to each of the predominant forms of tegumentary leishmaniasis in the region were studied. Specifically, clade A contains primarily isolates from individuals with DL, clade B contains isolates from CL subjects, and isolates from individuals with ML are concentrated in clade C (Schriefer et al., 2004).

Successful infection and ultimate dissemination of microorganisms throughout the host likely depends, in part, on the very early parasite–host cell interactions. Other studies have documented different patterns of gene expression in host macrophages after phagocytosis of different *Leishmania* species (Chaussabel et al., 2003; Ettinger and Wilson, 2008). Due to the severity and the diverse clinical forms of disease caused by *L. braziliensis*, we hypothesized that macrophage responses to the *Leishmania* subgenus *Viannia* *braziliensis* might be unique, and that these responses may differ between infections initiated by the distinct clades of *L. braziliensis*. The purpose of the current study, therefore, was to characterize and compare the initial changes in macrophage gene expression after phagocytosis of the three distinct *L. braziliensis* isolates from the three different clades. Similar to other investigators, we chose to focus on changes that occur at the earliest steps of infection based on the assumption that the patterns of gene expression at the very onset of infection initiate the environment that locally lead to the immunopathologic changes that occur later in disease.

## Materials and Methods

### Parasites

*Leishmania braziliensis* isolates were originally derived from individuals with CL, ML, or DL diagnosed in the medical clinic in Corte de Pedra, Bahia, Brazil. The three types of leishmaniasis were defined are as follows. Localized cutaneous leishmaniasis consisted of an ulcerated skin lesion at a single body site with no more than two secondary or satellite lesions, without clinical evidence of mucosal involvement. Mucosal leishmaniasis was defined as the presence of an inflamed or ulcerated mucosal lesion at a site that was non-contiguous with any cutaneous lesion. ML most frequently involved the nasal septum, oropharyngeal cavity, and/or larynx. Disseminated leishmaniasis was defined as 10 or more skin lesions of mixed type (acneiform, papular, nodular, and/or ulcerated) located in two or more body parts (head, trunk, arms, and legs). A diagnosis of tegumentary leishmaniasis was made by isolation of parasites in culture from an aspirate or biopsy of a cutaneous or mucosal lesion. Additionally, all patients had a positive delayed hypersensitivity skin response to leishmania antigen (Montenegro Test).

*Leishmania braziliensis* isolates used in the present study were cultured from aspirates of lesion borders suspended in liver infusion tryptose/Novy, McNeal, Nicolle (LIT/NNN) medium then expanded in Schneider’s medium complemented with 10% heat inactivated fetal calf serum and 2 mM L-glutamine. Species determination was based upon HSP-70 PCR-RFLP (Garcia et al., 2004; Montalvo et al., 2010) and confirmed by real time PCR (Weirather et al., 2011). Parasites were frozen in 10% DMSO, 90% growth medium in liquid nitrogen and thawed prior to macrophage infection studies. All studies were conducted with parasites in stationary phase of growth.

### Human Studies Approvals

Studies were approved by Institutional Review Boards of the Federal University of Bahia (document of approval: CAAE– 3041.0.000.054.07) and The University of Iowa. Study subjects were healthy adults over age 18, and written consent was obtained from all of them.

### Macrophages and Macrophage Infections

Peripheral blood mononuclear cells were isolated from normal healthy male volunteers from Salvador, Brazil, who resided outside of regions endemic for *L. braziliensis* infection. Monocytes were separated from peripheral blood by Ficoll hypaque density sedimentation, and adherence to plastic. Cells were maintained in Teflon vials in 20% autologous serum, 2 mM L-glutamine in RPMI 1640 with 100 U/ml penicillin and 100 μg/ml streptomycin (reagents from GIBCO). After a 5 days culture at 37°C and 5% CO_2_, differentiated monocyte-derived macrophages (MDMs) were suspended in 10% heat-inactivated fetal calf serum (Sigma-Aldrich, St. Louis, MO, United States), 2 mM L-glutamine in RPMI 1640 with 100 U/ml penicillin and 100 μg/ml streptomycin (Gibco/ThermoFisher, Waltham, MA, United States) [RP-10] and allowed to adhere to glass coverslips for 4 h at 37°C and 5% CO_2_. Non-adherent cells were removed by rinsing, then adherent MDMs were incubated overnight in RP-10 at 37°C, 5% CO_2_.

Monocyte-derived macrophages from each of four donors were incubated in RP-10 with stationary phase *L. braziliensis* promastigotes from each of the three clades at a 2:1 parasite:MDM ratio. Incubations were synchronized by centrifugation at 60 ×*g* for 4 min at 4°C, and transferred to 37°C, 5% CO_2_. The synchronization step greatly enhances the efficiency of parasite infection, enabling experiments to achieve infection of a majority of MDMs on the coverslip (Rodriguez et al., 2004). Control MDMs were treated in parallel but parasites were not added. After 1 h, free parasites were removed by rinsing and the RP-10 cultures were returned to 37°C, 5% CO_2_ for an additional 3 h.

Duplicate coverslips from MDM-parasite co-cultures were removed, fixed in methanol and stained with Wright Giemsa (Diff Quik Hema 3, Fisher Scientific) to document the parasite loads. From the remaining samples, total RNA was extracted with Trizol (Invitrogen, Carlsbad, CA, United States). RNA was treated with DNaseI and purified with Qiagen RNeasy mini-kit (Qiagen, Hilden, Germany). The RNA quality was checked with the Agilent Model 2100 Bioanalyzer (Agilent Technologies, Palo Alto, CA, United States). The numbers of MDMs with internalized parasites were quantified microscopically. Sets of samples from a single donor in which all three infected conditions contained at least 80% of the macrophages with intracellular parasites, and in which all four conditions generated highly pure RNA, were chosen for microarrays.

### Microarrays

cDNA was generated from 50 ng of total RNA and amplified with SPIA, a small sample PCR-based isothermal amplification method, using the Ovation Pico RNA Amplification System, v2 (NuGEN Technologies, Cat. #3100) according to the manufacturer’s protocol. The amplified SPIA cDNA product was purified through a QIAGEN QIAquick PCR Purification column (QIAGEN Cat #28104), according to modifications from NuGEN, then 3.75 μg of this product were fragmented (average fragment size = 85 bases) and biotin labeled using the NuGEN FL-Ovation cDNA Biotin Module, v2 (NuGEN Technologies, Cat. #4200) per the manufacturer’s protocol. The resulting biotin-labeled cDNA was hybridized to the Human U133+2.0 arrays (Cat #900470) at 45°C for 18 h. Arrays were washed and subject to secondary labeling with fluorescent probes per the Affymetrix protocols. Arrays and associated procedures were performed at The University of Iowa DNA Core facility.

After quality assessment using *affyQCReport* (Gautier et al., 2004), data were imported into Partek (PartekGS, St. Louis, MO, United States) and normalized using gcRMA (Irizarry et al., 2003). Arrays were compared by both ANOVA and paired *t*-test models, using the Partek batch correction feature to correct for the different hybridization sets. Significance was assessed based on *p*-value with step-up FDR multiple testing correction and fold change cutoffs. Identification of cellular pathways possibly affected in macrophages by infection with parasites was carried out using Ingenuity Pathway Analysis (IPA, QIAGEN). All transcript abundance results consist in the positive or negative fold change (i.e., ratio) in the expression of the genes in infected as compared to non-infected MDMs.

The affymetrics DNA micro array chip includes internal controls of base line gene expression across samples, that include GAPDH and β-actin, besides a number of internal positive and negative controls at the edges of the chip. This allows the ‘affyQCReport’ software tool to assess basal gene expression level within and between chips, using the GAPDH/β-actin expression ratios, and to evaluate the uniformity of test cDNA hybridization, and thus the expression reading throughout the entire chip area. Only those experiments that passed these tests were further analyzed in this study.

### Validation of Selected Changes in Gene Expression

Transcripts selected for validation were significantly expressed in all samples, and changes in gene expression were statistically significant considering all four replicate samples in microarrays by ANOVA. Changes in expression observed on microarrays were validated by reverse transcriptase followed by qPCR to document gene expression in RNA samples from replicate MDM samples incubated without or with the representatives of *L. braziliensis* clades. cDNA was generated using the Superscript III First Strand Synthesis System kit (Invitrogen/ThermoFisher, Waltham, MA, United States) and random hexamers, followed by RNase H treatment according to the manufacturer’s instructions. Taqman and primer pairs for qPCR were purchased from Applied Biosciences, Inc. (ABI, Foster City, CA, United States). Data were analyzed using the ΔΔCT method (Tricarico et al., 2002).

### Statistical Analyses Other Than Microarrays

Differences in the percent of macrophages infected, or in the numbers of intracellular parasites per infected macrophage between the clades were compared using chi-square or one-way ANOVA, respectively. RT-qPCR expression data were analyzed for significant changes between donors using ANOVA. Changes in expression of individual transcripts were tested for significance by paired *t*-test. Comparisons of gene expression profiles between clades employed Friedman’s and paired one-tailed Wilcoxon tests. For experiments other than microarrays, comparisons were considered significant at *p* < 0.05. Statistical analyses were performed with either Partek or Prism GraphPad software.

## Results

### MDM Infections

**FIGURE 1:**
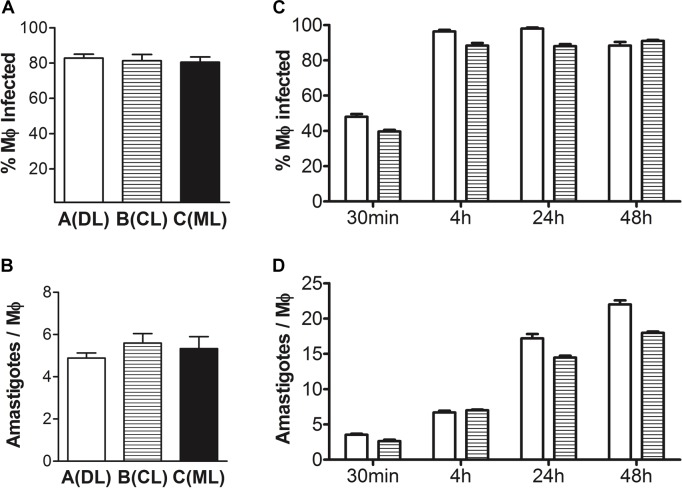
Parasite loads in infected MDMs used for microarrays. **(A)** The percentage of macrophages infected at a 2:1 (parasite:macrophage) ratio for 4 h with each isolate of *Leishmania braziliensis* from clades A, B, or C was quantified microscopically in four replicated donors (chi-square, *p* > 0.05). **(B)** Mean number of intracellular *L. braziliensis* parasites per infected macrophage in A (one-way ANOVA, *p* > 0.05). **(C)** Kinetics of the proportion of macrophages infected with isolates of *L. braziliensis* from clades A or B in four replicated donors, at a 2:1 (parasite:macrophage) ratio for 30 min to 48 h (chi-square from 4 to 48 h data points, *p* > 0.05). **(D)** Kinetics of the mean number of intracellular *L. braziliensis* parasites per infected macrophage in C (one-way ANOVA, *p* < 0.05; Pearson’s correlation, *R*^2^= 0.93, *p* = 0.037, for data combined between clades A and B parasites represented in the figure versus time post-infection). Data show mean ± SE after quantifying at least 400 macrophages per condition in MDMs from each donor.

Peripheral blood monocyte derived macrophages from four human donors were incubated *in vitro* with medium alone, or with each of three strains of *L. braziliensis* representative of clades A, B, or C under conditions promoting parasite phagocytosis. The three parasite strains used for all infections included one derived from an individual with CL (clade B), one from an individual with ML (clade C) and one from a subject with DL (clade A). Considering our prior observation that the 4 h time was optimal for microarray studies using *L. infantum* (Ettinger and Wilson, 2008), we chose the 4 h time point to compare responses to different strains of *L. braziliensis.* **Figure 1** shows that a large proportion of the MDMs contained intracellular parasites (**Figure 1A**; clade A 83 ± 5%, clade B 81 ± 8%, clade C 81 ± 7%, mean ± SD), with a mean of 5 parasites in each cell (**Figure 1B**; clade A 5.0 ± 0.6, clade B 5.6 ± 1.1, clade C 5.3 ± 1.4, mean ± SD). Neither the percent of MDMs infected nor the number of intracellular parasites per macrophage differed statistically between donors or between parasite clades within each donor (chi-square and one-way ANOVA, respectively).

We then checked changes in levels of MDM infections overtime. The proportion of MDM infected with *L. braziliensis* peaked at 4 h and remained constant up to 48 h, when last assessed (**Figure 1C**; chi-square, *p* > 0.05). However, the numbers of parasites per infected MDM continuously increased throughout the kinetics experiment (**Figure 1D**; one-way ANOVA, *p* < 0.05; Pearson’s correlation, *R*^2^= 0.93, *p* = 0.037, for data combined between clades A and B parasites represented in the figure versus time post-infection).

### Global Changes in Gene Expression Among Infected MDM

All transcripts that were significantly expressed in infected MDMs were considered in our analyses. Data were first analyzed within each donor for the change in expression between the infected versus the uninfected MDM conditions for each clade. Transcripts that were statistically significantly changed considering all four donors were determined using ANOVA with FDR multiple testing correction. Changes in gene expression induced by the three different clades in all four donors were compared using ANOVA. All transcripts noted in this report were selected because of a significance level of at least *p* < 0.0001. Most changes were at least twofold (i.e., Log_2_ = 1.0), with exceptions noted in specific instances.

Most transcripts were unchanged by infection, as expected. Examples of transcripts encoding proteins relevant to cellular functions that did not change in macrophages infected with any of the three clades included MTCH2 (mitochondrial carrier 2; overall *p* = 0.9562), TLL5 (tubulin tyrosine ligase-like family, member 5; overall *p* = 0.9556) and PLOD2 (procollagen-lysine, 2-oxoglutarate 5-dioxygenase 2; overall *p* = 0.9552) amongst many others.

More than 500 expressed transcripts were significantly changed to the *p* < 0.0001 level upon infection with any of the three strains of *L. braziliensis*. A comprehensive list of transcripts that changed and met the above significance criteria can be found in the **Supplementary Table 1**. Among the 576 altered transcripts, 487 were altered in macrophages infected with clade A, 156 in macrophages infected with clade B and 318 in macrophages infected with clade C. Considering all the transcripts whose abundance changed by the significance criteria, 471 transcripts were down-regulated whereas 89 were up-regulated. Significantly altered transcripts were classified according to functional pathways. The host processes affected by *L. braziliensis* phagocytosis distributed into four main categories: signal propagation, mitochondrial function, apoptosis, and response to external environment. The predominant pathways and genes whose expression was altered by *L. braziliensis* infection, and the changes in expression for each clade, are listed in **Table 1**.

**Table 1:** Examples of human monocyte derived macrophage genes whose expression was significantly changed by infection with *L. braziliensis* belonging to clades A, B, or C from Corte de Pedra (Statistical analyses by one-way ANOVA).

Functional group	Gene	*p*-Value			Log_2_ change in expression relative to uninfected MDMs			Gene description
		A	B	C	Clade			
					A	B	C	
**Decreased expression**								
**Signal propagation**								
Surface receptors	TLR8	9.15004e-005	0.0017291	8.18538e-005	-2.5	-1.9	-2.5	Toll-like receptor 8
	IL-12RB1	5.44261e-005	0.000572767	0.00147462	-1.8	-1.5	-1.5	Interleukin 12 receptor, beta one subunit
	IL-15RA	2.01434e-005	0.000597875	0.000135149	-2.2	-1.7	-1.9	Interleukin 15 receptor, alpha subunit
Signal transduction	MYD88	3.33232e-005	0.000460159	0.000161814	-1.7	-1.5	-1.6	Myeloid differentiation primary response (88); Activates NF-kB
	PELI1	0.000140615	0.00015842	4.70314e-005	-1.9	-1.9	-2.1	Pellino-related intracellular-signaling molecule; Activates NF-kB
	ECT2	6.22083e-005	0.000954735	0.00019954	-2.6	-2.0	-2.3	Epithelial cell transfer sequence 2 oncogene; Activates NF-kB
	VISA	0.000291381	4.07134e-005	0.000187217	-1.7	-2.0	-1.8	Virus-induced signaling adapter; Activates NF-kB
	NOD2	3.23249e-005	9.92379e-006	1.90858e-005	-2.4	-2.7	-2.5	Nuclear-binding oligomerization domain contining 2; Activates NF-kB
	STAT1	6.06884e-005	0.00179476	3.96141e-005	-1.4	-1.2	-1.4	Signal transduction and activation of transcription 1 JAK-STAT pathway
	STAT5A	3.97644e-005	0.00148146	0.000634802	-1.6	-1.4	-1.4	Signal transduction and activation of transcription 5 JAK-STAT pathway
	TYK2	2.32594e-005	0.000160678	3.71196e-005	-1.8	-1.6	-1.7	Non-receptor member of JAK family; JAK-STAT pathway
	JAK2	2.8929e-005	6.75267e-005	1.48982e-005	-2.2	-2.1	-2.4	Janus kinase 2; JAK-STAT pathway
	MAP2K5	0.00868219	0.000126235	9.53199e-006	-1.2	-1.5	-1.7	Mitogen-activated protein kinase kinase 5; MAPK pathway
	MAP2K3	6.23796e-005	0.000128993	6.32058e-005	-1.9	-1.8	-1.9	Mitogen-activated protein kinase kinase 3; MAPK pathway
	ICK	7.68298e-005	0.000600357	3.15881e-005	-2.3	-1.9	-2.5	Intestinal cell (MAK-like) kinase; Possibly MAPK pathway
	TRAF7	2.50164e-005	0.000317723	0.0002048	-1.4	-1.3	-1.3	TNF receptor-associated factor 7; MKKK of MAPK pathway
	PLCB2	1.7885e-005	5.43495e-005	3.08817e-006	-1.7	-1.6	-1.9	Phospholipase C, beta 2 chain; Activates Phospholipase C
	CAMKK2	8.90843e-006	0.000102443	1.61629e-005	-1.9	-1.6	-1.8	Calcium/calmodulin-dependent protein kinase kinase 2
	MFNG	4.41145e-005	0.00259564	0.00011307	-1.9	-1.5	-1.8	Manic fringe; Notch signaling pathway
**Transcriptional regulators**	TRERF1	4.08183e-005	0.00106701	0.000153032	-1.9	-1.6	-1.5	Transcriptional regulator
**Nuclear pore function**	XPO6	7.73493e-005	0.000158596	3.54427e-005	-1.5	-1.5	-1.6	Exportin 6; nuclear pore protein transporter
	RANBP10	2.96201e-005	0.000235132	0.000147206	-2.0	-1.7	-1.8	RAN binding prot 10; nuclear pore transport
	NUP62	4.25576e-005	0.000737207	0.000128261	-1.4	-1.3	-1.4	Nucleoporin 62 kDa
	NUP93	6.71235e-005	0.00070184	0.000213948	-1.6	-1.4	-1.5	Nucleoporin 93 kDa
	NUP214	1.07315e-005	0.00204607	0.00011174	-1.8	-1.3	-1.6	Nucleoporin 214 kDa
**Mitochondrial function**								
Electron transport	ISCU	5.90789e-006	4.1835e-005	1.09868e-005	-1.5	-1.4	-1.5	Iron–sulfur cluster scaffold homolog
	ISCA2	1.49189e-005	0.000379011	2.9555e-005	-2.4	-1.8	-2.3	Iron–sulfur cluster assembly 2 homolog
	NDUFA11	1.61998e-005	0.000285867	7.42091e-006	-1.4	-1.3	-1.4	NADH dehydrogenase subunit, mitochondrial encoded
	NDUFC1	3.5465e-005	0.000959163	4.37207e-005	-1.4	-1.3	-1.4	NADH dehydrogenase subunit, mitochondrial encoded
	NDUFB10	9.64108e-006	0.000432758	5.43291e-005	-1.5	-1.3	-1.4	NADH dehydrogenase subunit, mitochondrial encoded
	NDUFV3	4.55964e-006	0.000170585	1.11309e-005	-1.8	-1.5	-1.7	NADH dehydrogenase subunit, mitochondrial encoded
	NDUFS3	3.86571e-007	1.31216e-005	8.56046e-007	-1.6	-1.4	-1.5	NADH dehydrogenase subunit, mitochondrial encoded
	NDUFS2	3.55512e-005	0.000983593	0.000288838	-1.7	-1.4	-1.5	NADH dehydrogenase subunit, mitochondrial encoded
	NDUFB8	8.34828e-005	0.000590415	0.0012524	-1.3	-1.2	-1.2	NADH dehydrogenase subunit, mitochondrial encoded
	TFAM	3.42623e-005	0.00349217	8.02956e-005	-1.5	-1.3	-1.5	Transcription factor A, mitochondrial; encoded in the nucleus
Mitochondrial protein	MRPS14	8.24489e-005	0.000132693	0.000467884	-2.3	-2.2	-2.0	Mitochondrial ribosomal protein S14; encoded in the nucleus
synthesis	MRPS35	1.0742e-005	0.00014928	1.28405e-005	-1.5	-1.4	-1.5	Mitochondrial ribosomal protein S35; encoded in the nucleus
	MRPL16	2.17496e-005	0.000230342	8.89629e-005	-2.0	-1.7	-1.8	Mitochondrial ribosomal protein L16; encoded in the nucleus
	MRPL19	0.000265737	0.00231705	7.0312e-005	-1.8	-1.5	-1.9	Mitochondrial ribosomal protein L19; encoded in the nucleus
	MRPL41	4.5838e-005	0.00148749	0.000301997	-1.7	-1.4	-1.5	Mitochondrial ribosomal protein L41; encoded in the nucleus
	MRPL52	7.55371e-005	0.00318154	0.00012328	-1.5	-1.3	-1.5	Mitochondrial ribosomal protein L52; encoded in the nucleus
	MRP63	8.37002e-005	0.00353342	0.000356301	-1.6	-1.4	-1.5	Mitochondrial ribosomal protein 63; encoded in the nucleus
	PDHB	8.56049e-005	0.00209902	0.000120183	-1.8	-1.5	-1.7	Pyruvate dehydrogenase (lipoamide) beta
**Apoptosis**								
	TP53	0.000833372	0.0018592	7.78129e-005	-1.5	-1.5	-1.8	Tumor protein p53; Tumor suppressor
	MAPK14	2.07957e-005	0.000172754	5.78548e-005	-1.7	-1.5	-1.6	Phosphorylates and activates TP53
	FRAP1	7.21564e-005	0.000895603	0.000148447	-2.2	-1.8	-2.0	Phosphorylates and activates TP53
	YPEL3	1.10105e-005	0.000141583	2.17379e-005	-2.9	-2.2	-2.7	Tumor suppressor directly induced by TP53
	MRPL41	4.5838e-005	0.00148749	0.000301997	-1.7	-1.4	-1.5	TP53 translocation to mitochondrion
**Parkinson’s disease**								
	LRRK2	0.000111713	0.00051427	7.7878e-005	-3.9	-3.1	-4.1	PARK8; Induces apoptosis; Interacts with PARKIN and DJ-1
	DJ-1	8.46957e-006	0.0013984	0.000116928	-1.3	-1.2	-1.2	PARK7; Redox-sensitive chaperone and sensor of oxidative stress
	NUB1	0.000155931	0.000373989	6.53521e-005	-1.7	-1.6	-1.7	Negative regulator of ubiquitin-like proteins 1; Part of Lewy bodies
**Increased expression**						
**Transcriptional regulation**	HIC1	5.26337e-005	0.000501033	0.00023281	3.6	2.7	2.9	Transcription repressor Hypermethylated in Cancer 1
**Apoptosis**	ETS2	4.80848e-005	0.000607089	0.000184372	2.4	1.9	2.1	Probably transcriptional activator of TP53
**Proteasomal degradation**								
	UBC	7.4032e-005	0.00237149	0.000111829	1.2	1.1	1.2	Ubiquitin C; proteasome
	UBAP1	3.48504e-005	0.000293469	0.000225023	1.5	1.4	1.4	Ubiquitin associated protein 1 target ubiquitinilation
	SPSB1	7.87991e-005	0.000339641	0.000157297	2.4	2.1	2.2	Substrate recognition-E3 ubiquitin–protein ligase complex
	KLHL21	5.79205e-005	0.000134488	3.97511e-005	1.7	1.6	1.7	Kelch-like 21; Adapter of an E3 ubiquitin-protein ligase complex
	USP12	9.3778e-005	0.000972177	0.000104449	1.6	1.4	1.5	Ubiquitin specific peptidase 12; Deubiquitinating enzyme
**Environmental response**								
Metal binding	MT1M	9.05665e-005	0.000215829	2.07091e-005	7.6	6.3	11.0	Metallothionein 1M
	MT1X	0.000232234	0.000598304	5.30768e-005	2.7	2.4	3.2	Metallothionein 1X
	MT1F	0.000211204	0.000573519	4.83875e-005	2.6	2.4	3.2	Metallothionein 1F
	MT1E	8.88948e-006	2.39768e-005	2.1035e-006	3.4	3.0	4.2	Metallothionein 1E
	MT1G	1.43444e-006	6.48199e-006	4.16245e-007	2.7	2.4	3.1	Metallothionein 1G
	MT1H	1.17455e-005	4.00175e-005	3.2461e-006	2.7	2.4	3.1	Metallothionein 1H
Antioxidant response	TXNRD1	5.75177e-006	6.15629e-006	2.27237e-006	1.3	1.3	1.4	Thioredoxin reductase 1
Stress response	HSPA1A	7.71182e-005	0.000256787	5.82824e-005	5.8	4.6	6.1	Heat shock 70 kDa protein 1A

### Down-Regulated MDM Transcripts

**FIGURE 2:**
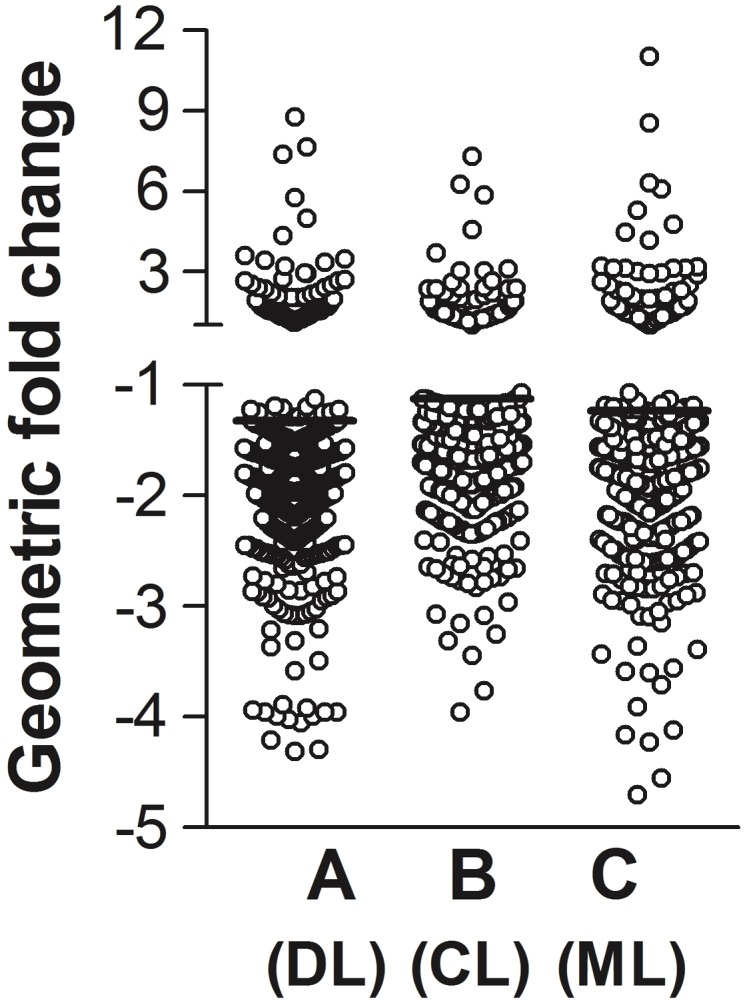
Global changes in infected MDM gene expression. MDMs from four healthy human donors were infected at a 2:1 (parasite:MDM) ratio with each of three isolates of *L. braziliensis* from clades A, B, or C (Schriefer et al., 2004). The clades A, B, and C isolates were drawn from patients with DL, CL, and ML, respectively. After 4 h, total RNA was extracted and processed for hybridization to Affymetrix human transcript microarrays. Fold changes were calculated by comparing fluorescence data representing the abundance of each transcript in infected versus uninfected MDMs from the same donor. Each dot in the figure represents the average fold change in abundance of each transcript in all four donors. Eighty-nine transcripts were significantly increased, and 471 transcripts were significantly decreased after MDM infection with each of the three *L. braziliensis* isolates (one-way ANOVA for repressed transcripts among MDM infected with clades A, B, or C, *p* < 0.0001).

The overwhelming majority (84.2%) of transcripts that were significantly altered by infection with any of the three *L. braziliensis* clades were repressed (**Figure 2**). Selected noteworthy transcripts, and *p*-values for each listed transcript (ANOVA), are displayed in **Table 1**. Genes belonging to three of the four categories mentioned above were predominantly down-regulated. The category with the largest number of significantly down-regulated transcripts encoded components of cellular signaling pathways. These included mRNAs encoding cell surface receptors, signal transduction proteins and one transcriptional regulator. Transcripts encoding nuclear permeability factors were also diminished.

The most highly repressed transcript encoding a cell surface receptor was Toll-like receptor 8, the receptor that senses single stranded RNA within the host cell. Also affected was the transcript for MyD88, a binding protein necessary for function of many TLRs, including TLR8 (Kenny and O’Neill, 2008). Transcripts encoding cytokine receptors or signaling intermediates in cytokine-initiated cascades that were down-modulated included subunits of receptors for IL-12 (IL12Rβ1), IL15 (IL15Rα), and IL-10 (IL10Rβ). Proteins transducing signals from cytokine receptors were also down-regulated, most notably Jak2, STAT1, STAT5A, TYK2, and two MAP kinase kinases (MAP2K5 and MAP2K3). Both the TLR and Jak-STAT pathways can converge on the transcription factor NF-κB (Oeckinghaus et al., 2011); thus these modifications could result in diminished pro-inflammatory responses. Overall, forty transcripts encoding signaling intermediates or transcriptional regulators were significantly affected at a *p* < 0.0001 level, and 35 of these (87.5%) were down-regulated. Surprisingly, transcripts encoding components of nuclear pores were also down-modulated by *L. braziliensis* infection, including two transcripts encoding Ran binding proteins (nuclear exportin 6, XPO6; and RBP10) and three nucleoporins (NUP62, NUP93, and NUP214).

Several transcripts encoding proteins that function in the mitochondrion were significantly decreased. These included proteins important for mitochondrial oxidative phosphorylation. The down-modulated nuclear-encoded proteins ISCU and ISCA2 are two of the three peptides that are translocated into the mitochondrion to serve as scaffold proteins for the biogenesis of iron–sulfur clusters (Rouault and Tong, 2005). These clusters are involved in electron transfer chains of both Complex I and Complex II during oxidative phosphorylation. In addition to the above, expression of all seven mitochondrially-encoded subunits of the NADH dehydrogenase constituting Complex I were significantly suppressed, possibly decreasing essential components of the mitochondrial electron transport chain (Andreyev et al., 2005). Transcripts encoding additional proteins responsible for mitochondrial protein synthesis were also suppressed, including one of two subunits of the major mitochondrial transcription factor TFAM and seven mitochondrial ribosomal proteins (MPRS 14, 35; MPRL 16, 19, 41, 52; MRP 63). Complex I and other mitochondrial proteins are essential for translocating protons across the mitochondrial inner membrane and generating the electrochemical potential gradient necessary for ATP production, generation of reactive oxygen species, and promoting apoptosis (Chomova and Racay, 2010). Thus, the above observations could have implications for macrophage programmed cell death, as well as cellular metabolic activity.

Other transcripts involved in the intrinsic apoptosis pathway (Jin and El-Deiry, 2005) that were suppressed by *L. braziliensis* exposure included TP53, which encodes tumor protein 53 or p53, and LRRK2, the major late-onset familial Parkinson’s disease associated gene [also called PARK 8 (Paisan-Ruiz et al., 2004; Zimprich et al., 2004)]. Expression of proteins that participate in p53 function were also decreased: MAPK14 and FRAP1 phosphorylate and activate p53; YPEL3 is induced by p53; MRPL41 stabilizes p53 and enhances its translocation into the mitochondrion (Kelley et al., 2010). LRRK2 (PARK 8) is hypothesized to play a role upstream of the MAPK pathway and to mediate both familial and sporadic Parkinson’s disease by inducing intrinsic neuronal apoptosis (Zimprich et al., 2004; Healy et al., 2008; Lin et al., 2009). Other genes that associate with Parkinson’s disease that were also repressed were NUB1 which encodes a Lewy body protein, and DJ-1 (aka PARK7) whose product can bind LRRK2 and is associated with a recessive form of Parkinson’s disease (Bonifati, 2007).

### Up-Regulated MDM Transcripts

Only 15.8% of transcripts significantly influenced by MDM infections were up-regulated (**Figure 2** and **Table 1**). Noteworthy up-regulated transcripts include the negative regulator of transcription HIC1, and a transcription factor activating the apoptosis protein TP53, ETS2 (Venanzoni et al., 1996). It is possible that the latter was induced in response to down-regulated expression of TP53 itself. Many of the other up-regulated transcripts encoded genes involved in response to environment conditions, sometimes involved in the cellular response to stress. Similar to our prior report, transcripts encoding several metallothionein proteins were highly up-regulated (Ettinger and Wilson, 2008). Metallothioneins are important for metal chelation and regulating cellular content of zinc. Because of their high cysteine content, metallothioneins not only chelate metal ions, but in some situations they can play a protective role against oxidant toxicity (Namdarghanbari et al., 2011). The transcript for thioredoxin reductase was also increased, as was the transcript of the HSP70 family member HSPA1A. Several transcripts encoding proteins associated with ubiquitin targeting were increased, suggesting an increase in proteins acting as chaperones for misfolded or damaged proteins targeted for degradation in the proteasome (Wong and Cuervo, 2010).

### Validation of Micro-Array Findings

Selected microarray findings were validated in two manners. First, amongst the transcripts that were significantly changed by *L. braziliensis* infection, some were chosen for validation using the original mRNAs employed in microarray hybridization. The transcripts were chosen to reflect a spectrum ranging from highly induced to highly repressed. Validation of mRNA changes was done by reverse transcriptase-qPCR. The mean fold changes according to each method are shown in **Table 2**. In all 30 conditions evaluated by RT-qPCR, the relative abundance of transcripts changed in the same direction as the microarray. Not surprisingly, there were differences in the magnitude of fold change between the two methods. Nonetheless the 10 transcripts fell into the same approximate rank order from highest to lowest fold change in expression, differing in order by 0–2 positions when aligned according to microarray versus RT-qPCR data.

**Table 2:** Changes in expression of 10 transcripts in MDMs infected with *L. braziliensis* isolates belonging to each clades (A, B, or C) were documented in independent assays of gene expression in four MDM donors.

Transcript	Clade	Fold change	
		Microarray (*n* = 4)	qPCR (*n* = 4)
MT1M	A	7.653	2.660
MT1M	B	6.269	2.405
MT1M	C	11.048	4.945
HSPA1A	A	5.763	4.869
HSPA1A	B	4.563	3.249
HSPA1A	C	6.101	4.414
MT1X	A	2.662	3.880
MT1X	B	2.384	2.898
MT1X	C	3.203	5.642
TXNRD1	A	1.337	1.524
TXNRD1	B	1.334	1.560
TXNRD1	C	1.377	1.855
UBC	A	1.179	1.444
UBC	B	1.111	1.283
UBC	C	1.170	1.351
DJ-1	A	0.406	0.843
DJ-1	B	0.435	0.949
DJ-1	C	0.435	0.985
TRERF1	A	0.268	0.453
TRERF1	B	0.330	0.486
TRERF1	C	0.354	0.574
IL-15RA	A	0.435	0.624
IL-15RA	B	0.536	0.541
IL-15RA	C	0.467	0.694
TLR8	A	0.178	0.435
TLR8	B	0.268	0.458
TLR8	C	0.178	0.482
LRRK2	A	0.067	0.537
LRRK2	B	0.117	0.553
LRRK2	C	0.058	0.482

*Data show the mean (log_2_)-fold induction of transcripts in infected relative to uninfected MDMs 4 h after parasite exposure. Results from the original DNA microarrays (3^*rd*^ column) are compared to new reverse transcriptase qPCR assays (4^*th*^ column). All changes from uninfected were statistically significant (microarray: see **Table 1**; qPCR results: *p* < 0.05 by paired *t*-test)*.

**FIGURE 3:**
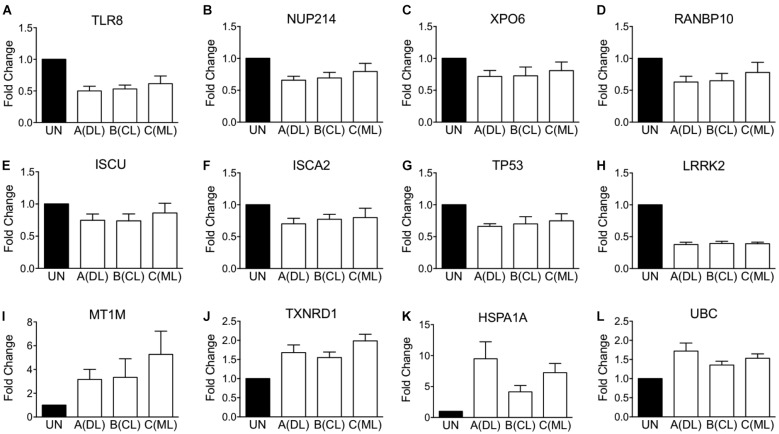
qPCR validation of changes in gene expression observed on microarrays. Validation performed using RNA samples of additional blood donors different than those included in microarray analysis shown in **Figure 2**. **(A–H)** Shows RT-qPCR of selected MDM transcripts whose expression was down-regulated, according to Affymetrix microarrays. **(I–L)** Shows RT-qPCR of selected MDM transcripts whose expression was up-regulated. Data consist in the average fold change elicited by each *L. braziliensis* clade representative relative to uninfected MDMs from the same donor.

Second, beyond validation with original microarray samples, we tested whether there would be similar changes in gene expression in MDMs from additional human blood donors. Selected transcripts that were validated by RT-qPCR are shown in **Figure 3**, showing down-modulated transcripts in panels A through H, and up-regulated transcripts in panels I through L. Although some of the minimally changed did not reach significance compared to uninfected MDMs, the overall directions of changes were concordant between data from new donors and original microarray results.

### MDM Transcripts Regulation According to Infection With *L. braziliensis* Clade

**FIGURE 4:**
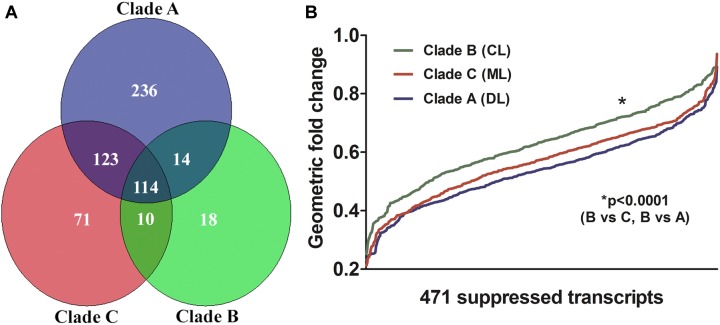
Gene expression profiles from the microarrays described and illustrated in **Figure 2** were collated according to the number of significantly altered transcripts in MDM infected with isolates from each of the three representatives of *L. braziliensis* clades (A, B, or C). **(A)** Venn diagram of the distribution of transcripts with changes in expression that reached statistical significance upon infection of MDMs with each *L. braziliensis* parasite. Sectors indicate the numbers of transcripts that were uniquely changed due to infection with one parasite clade, or transcripts that were changed by infection with more than one parasite clade (evaluation of gene expression employed ANOVA for detecting transcripts significantly affected by infections, and paired Student’s *t*-test for comparing the expression elicited by clades of parasites in infected MDM). **(B)** The magnitude of change in expression of 471 genes in MDM infected with each of the three *L. braziliensis* isolates is illustrated. Values represent the fold changes in expression of the 471 genes for which transcript abundance was significantly decreased by infection with any of the three parasite isolates tested. *L. braziliensis* isolates belong to clade A (DL; blue), clade B (CL; green), or clade C (ML; red). Each position on the x-axis corresponds to a single gene, plotted against its fold change in expression on the y-axis (Friedman’s test *p* < 0.002 for pair-wise comparisons between MDM infected with different parasite clades).

Transcripts of MDMs infected with *L. braziliensis* isolates belonging to each of the three different clades were regulated in the same general direction (induction/repression). However, the magnitude of change for many transcripts differed between the clades (**Figure 4**). Aggregate analysis of the MDM gene expression profiles, employing the built-in clustering capability of the Partek Genomics Suite (Partek, Inc., Chesterfield, MO, United States), was used to compare changes in gene expression induced by infection with parasite strains associated with metastatic diseases [clades A (DL), C (ML)] and with localized CL (clade B). As illustrated in **Figure 4A**, the overlap between transcripts significantly altered by infection with clades C and A was greater than the overlap between either clades C and B or clades A and B. The magnitude by which each of the parasite isolates suppressed the 471 down-modulated transcripts is plotted according to clade in **Figure 4B**. Although all changes were in the same direction, the plot illustrates that the magnitude of change was similar between isolates belonging to clades A and C, and both were more intense than changes induced by the clade B isolate. The difference between the suppressive effects of parasite isolates on gene expression was statistically significant (**Figure 4B**, Friedman’s test, *p* = 0.002; **Figure 2**, ANOVA, *p* < 0.0001).

**FIGURE 5:**
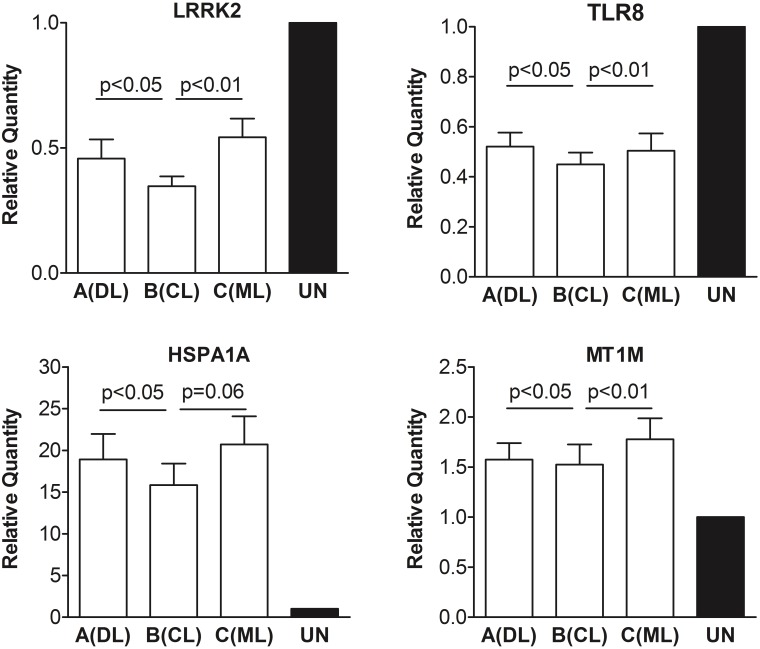
Changes in LRRK2, TLR8, HSPA1A, and MT1M expression were documented in independent assays of MDMs from eight additional human donors, distinct from those of experiments depicted in **Figures 1**–**4**. MDMs were infected with three representative *L. braziliensis* isolates of each clade (A, B, or C) used in microarray experiments. Data derived from total RNA extracted after 4 h of MDM infection at a MOI of two parasites per macrophage (2:1). The relative abundance of transcripts was assessed by RT-qPCR. *p*-Values correspond to pair-wise comparisons by one tailed paired Wilcoxon test.

We questioned whether additional parasite isolates belonging to clades A, B, or C would yield the same or different effects on macrophage gene expression. MDMs from eight new subjects were infected in parallel with nine *L. braziliensis* isolates, including three isolates from each of the three clades A, B, or C, respectively. The average fold changes in two of the most down-regulated transcripts (LRRK2 and TLR8) and two of the most up-regulated transcripts (HSPA1A and MT1M) were determined by RT-qPCR. In each case, the direction of fold change in MDMs from the new donors to a larger set of parasite isolates was similar to that observed in the original MDMs responding to the initial three parasite isolates (**Figure 5**). Furthermore, in each case the changes induced by clades A and C isolates were similar to each other, but different from changes induced by clade B (**Figure 5**).

### Kinetics of Selected Transcripts in Infected MDM

We evaluated the duration of change in expression of twenty significantly affected MDM genes up to 48 h post-infection of MDM from eight different donors. The transcripts were chosen to reflect the range of expressions detected by the micro array experiments. These data, shown in **Table 3**, indicate that the changes in expression of the chosen transcripts peaked at either 4 or 24 h. Considering all transcripts and all time points tested, the following transcripts reached significance: IL10RB, MYD88H, TRERF1, PARK7, UBC, MT1M, NDUFA11, and NUP214. Biological relevance would require a secondary study of protein abundance, but these data lead us to conclude that the kinetics of gene expression is dynamic over the first 48 h after macrophage infection, and that at least some transcripts reaching signficance at 4 h remained elevated or suppressed at 24 h.

**Table 3:** Expression kinetics of 21 transcripts significantly induced or repressed in human monocyte derived macrophages of eight donors after 4 h infection with an *L. braziliensis* isolate of a cutaneous leishmaniasis patient from Corte de Pedra, Brazil.

Transcript	4 h Mean (SE)	24 h Mean (SE)	48 h Mean (SE)	4 h × 24 h × 48 h *p*-value	4 h × 24 h *p*-value	4 h × 48 h *p*-value
LRRK2	0.44 (0.11)	0.64 (0.19)	0.30 (0.09)	0.2637	0.3751	0.3565
NUP214	0.55 (0.07)	1.17 (0.10)	0.86 (0.21)	0.0162*	0.0008***	0.1407
TRERF1	0.55 (0.11)	1.58 (0.40)	1.01 (0.27)	0.1443	0.0484*	0.1874
TP53	0.60 (0.10)	0.88 (0.15)	0.73 (0.19)	0.3708	0.1747	0.6276
IL10RB	0.60 (0.13)	1.18 (0.09)	0.53 (0.17)	0.0395*	0.0087**	0.8201
MAPK14	0.62 (0.12)	0.89 (0.21)	0.71 (0.17)	0.3497	0.2053	0.6732
TLR8	0.68 (0.15)	1.23 (0.29)	0.53 (0.18)	0.1667	0.2021	0.4237
NDUFA11	0.70 (0.08)	2.11 (0.57)	0.57 (0.19)	0.0490*	0.0430*	0.5232
PARK7	0.73 (0.05)	1.23 (0.07)	0.76 (0.19)	0.0304*	0.0001***	0.8683
XPO6	0.77 (0.10)	1.56 (0.47)	0.94 (0.30)	0.2905	0.1462	0.6111
MYD88H	0.85 (0.11)	1.64 (0.26)	0.68 (0.17)	0.0104*	0.0384*	0.4681
FRP1	0.86 (0.27)	1.61 (0.26)	0.94 (0.23)	0.1498	0.1212	0.8445
NUP93	1.02 (0.24)	1.06 (0.05)	0.98 (0.23)	0.9414	0.8573	0.9014
IL15RA	1.32 (0.40)	1.37 (0.28)	0.92 (0.22)	0.5385	0.9363	0.4214
NUB1	1.35 (0.37)	2.40 (0.56)	0.86 (0.27)	0.0929	0.2262	0.3887
UBC	1.39 (0.30)	3.27 (0.82)	0.77 (0.21)	0.0244*	0.0154*	0.1336
TXRND1	1.85 (0.28)	2.44 (0.68)	0.87 (0.25)	0.0621	0.3381	0.0730
MT1X	2.66 (0.69)	1.50 (0.30)	1.24 (0.41)	0.1087	0.1084	0.1095
MT1M	3.20 (0.71)	3.25 (1.04)	0.78 (0.29)	0.0214*	0.9564	0.0058**
HSPA1A	4.45 (2.14)	2.84 (0.90)	2.43 (1.44)	0.6523	0.5278	0.5245

*Statistical comparisons of three time points were done by ANOVA; comparisons between two times utilized Student’s *t*-test. ^∗^*p* < 0.05; ^∗∗^*p* < 0.01; ^∗∗∗^*p* < 0.001*.

## Discussion

Previous studies have directly or indirectly underscored the influences of both parasite and host on the clinical phenotypes of diseases caused by *L. braziliensis* (Kahl et al., 1991; Cabrera et al., 1995; Saravia et al., 1998, 2002; Schriefer et al., 2004, 2009; Castellucci et al., 2005, 2006; Salhi et al., 2008). In the present report, we tested whether the initial contact between human macrophages and genetically distinct *L. braziliensis* strains derived from individuals with different clinical forms of tegumentary leishmaniasis might lead to diverse gene expression patterns in the host macrophage. The *L. braziliensis* isolates we examined were chosen from representative individuals with either metastatic (disseminated or mucosal) forms of leishmaniasis, or localized (cutaneous) leishmaniasis. Each isolate had previously been typed as belonging to the three genetically distinct clades that correspond to the different forms of disease (A, C, or B, respectively) (Schriefer et al., 2004).

Remarkably, the parasites associated with the two forms of metastatic leishmaniasis (clades A and C) elicited changes in host macrophage gene expression that were more similar to each other than those induced by the isolate from a subject with localized cutaneous leishmaniasis (clade B). The differences primarily observed were in the magnitude of induction or repression of the affected transcripts, caused by the *L. braziliensis* from subjects with ML or DL as compared to the strain from a subject with CL.

*Leishmania braziliensis* infection down-regulated the expression of the majority of the significantly affected MDM transcripts. This is similar to the overall suppression of gene expression observed in other studies of murine and human macrophage infections with *Leishmania* spp. (Matlashewski and Buates, 2001; Chaussabel et al., 2003; Ettinger and Wilson, 2008; Gregory et al., 2008).

Prior work has suggested that there may be an overall suppression of pathways transducing signals from the extracellular environment in leishmania-infected macrophages. A report by Moore et al. was one of the earliest accounts indicating that *Leishmania* spp. infection of macrophages impairs the transmission of information from the macrophage surface to the nucleus (Moore et al., 1993). Subsequent reports showed that the initial contact between leishmania parasites and the host cell led to a transient activation of signaling mechanisms, but many signaling pathways are suppressed during established infection (Engwerda et al., 2004). Signaling through Jak/STAT and MAPK with resultant suppressed NF-κB activation are some of the most dramatically affected pathways (Junghae and Raynes, 2002; Bhardwaj et al., 2005; Forget et al., 2005; Ben-Othman et al., 2008; Calegari-Silva et al., 2009; Matte and Descoteaux, 2010). Changes leading to impaired regulatory mechanisms cited include protein phosphorylation state and targeting to the proteasome for degradation (Bhardwaj et al., 2005; Forget et al., 2005).

The current study also documents a general down-regulation of genes involved in transducing signals from TLRs or cytokines, all the way to the host cell nucleus. Taken together, these observations lead us to speculate that the changes in outside-inside signaling transcripts, primarily in a downward direction, creates an environment of anergy and paralyzes cellular functions just after host–cell invasion, which benefit the parasite.

Observations in the current study raise the hypothesis that infection with *L. braziliensis* may suppress or modify iron–sulfur clusters biogenesis and/or mitochondrial respiration. It remains to be seen whether subtle decreases in transcripts encoding components of Complex I is capable of affecting the generation of the electron potential at the mitochondrial inner membrane. Nonetheless evidence suggests that Complex I is involved in cellular apoptosis, and it is tempting to speculate that these changes might result in diminished mitochondrial signals leading to death of the infected cell. Indeed, the ability of *Leishmania* spp. infection to inhibit host cell apoptosis has long been recognized as a strategy promoting parasite persistence and survival (Moore and Matlashewski, 1994; Lisi et al., 2005; Ruhland et al., 2007).

Despite two reports showing that *L. major* infection of the murine RAW 264.7 macrophage cell line suppresses cytochrome c release from the mitochondrion, in part mediated by BCL-XL, preventing activation of caspases (Akarid et al., 2004; Donovan et al., 2009), our study did not reveal significant changes in expression of BCL-2 family members in human macrophages infected with *L. braziliensis*. However, transcripts encoding proteins involved in two major triggers of intrinsic pathway apoptosis were down-regulated. These were (1) TP53 and proteins that control its activation, stabilization, and translocation into the mitochondrion; and (2) LRRK2, which attaches to the mitochondrion and mediates apoptosis by a still poorly understood mechanism. Regarding LRRK2, SNPs in this gene are associated with the multibacillary form of human infection with *Mycobacterium leprae* (Zhang et al., 2009), another intracellular pathogen.

*Leishmania braziliensis* can lead to diverse manifestations in infected humans. CL can disseminate if left undiagnosed and untreated for an extended time period or when occurring in malnourished individuals. Disseminated forms of tegumentary leishmaniasis are also more common in individuals who harbor particular polymorphic risk-associated alleles compared to other genotypes (Llanos-Cuentas et al., 1984; Cabrera et al., 1995; Alcais et al., 1997; Turetz et al., 2002; Castellucci et al., 2005, 2006; Machado-Coelho et al., 2005; Salhi et al., 2008). Our prior reports suggested that genetic polymorphism of the parasite itself is also associated with the diversity of diseases caused by this parasite (Schriefer et al., 2004; Queiroz et al., 2012). The current report extends our understanding by showing that *L. braziliensis* strains belonging to distinct clades lead to subtle but consistently distinct behaviors upon interaction with human MDMs. The study leads to the hypothesis that prolonged macrophage survival due to a decrease in apoptosis, and a greater suppression of the responses to external stimuli may correspond to part of the mechanism that favor dissemination.

A practical limitation of this study was that full evaluation of the effects of parasite clade on global macrophage gene expression was performed with one representative of each major clade, using biological replicates to enable statistical evaluation. We were able to improve on this by doing validation with a larger set of strains per *L. braziliensis* clade, though validation was done on a limited number of transcripts. Although logistics did not allow us to examine a larger set of strains in this report, these data do lead us to relevant hypotheses that merit validation in further studies with a larger array of parasite lines from additional infected patients. Ongoing tests must be done before there will be ample comparisons leading to the mechanisms and consequences of host cell invasion by distinct groups of *L. braziliensis* isolates.

It must be emphasized that the current study focused on genetically distinct strains of *L. braziliensis*. These findings, as well as our previous molecular epidemiology observations on the associations between *L. braziliensis* genotypes and clinical forms of ATL (Queiroz et al., 2012; Guimaraes et al., 2016) undoubtedly work in concert with host genotype, health status of the host, coinfections, and epidemiologic factors to fully account for the polymorphic outcomes of infection. Examples of determinants shown to contribute different clinical phenotypes of *L. braziliensis* infection include concurrent infections (Meireles et al., 2017; Martinez et al., 2018), pregnancy (Morgan et al., 2007; Guimaraes et al., 2009), and the genetic background of the human host (Castellucci et al., 2005, 2006, 2014).

It should also be pointed out that these observations reflect the earliest events in the host–parasite relationship, and thus the immediate handling of infection by one of its preferred host cell types. These early events can initiate parasite killing or survival, and recruitment of additional host cells to infected tissues, to name some functional effects. In addition to the changes highlighted in Section “Results,” further studies could be used to probe differences that might lead to parasite dissemination versus containment at the local site. Relevant functions would include microbicidal response, survival of the infected cell, and recruitment of host cells that might promote parasite transport out of infected tissues (Charmoy et al., 2010; Goncalves et al., 2011; Ribeiro-Gomes et al., 2012). It is thus relevant that among transcripts up-regulated by infection with the “disseminating” clades A and C, but to a lesser extent clade B (localized disease), are a number of macrophage heat shock or stress response proteins, whereas transcripts relevant to inflammatory response pathways (TLR8, STAT1, TNFSF13B, SYK) in macrophages were down-regulated. These observations will warrant validation, followed by further examination of cellular interactions with respect to function of the implicated pathways. We expect that infected macrophages from chronically infected animals would present quite different patterns of gene expression, reflecting the inflammatory processes occurring in infected tissues at these time points. As there are multiple differences in the degree of transcript expression between clades A–C and clade B, we hypothesize that subtle differences in the degree of transcript suppression or stimulation may collectively contribute toward differences in disease manifestations including parasite dissemination. Finally, our observations also warrant future examination of protein abundance and/or function of the implicated pathways.

## Author Contributions

RS and VA performed parasite and macrophage isolation and cultivation, infection experiments, cultured cells RNA extractions, and data analyses. TB performed DNA micro array experiments and data analyses. NE standardized the macrophage culturing and differentiation in the lab, and helped in the micro array data analyses. LG and LA helped in macrophage culturing and infection experiments, and performed annotation of significantly expressed genes and pathways. LHG, PM, and EC were responsible for clinical work in the field that resulted in all isolates of *L. braziliensis* explored in the study, and were involved in the design of the research. MW and AS were responsible for team coordination, study design, data interpretation, and manuscript preparation.

## Conflict of Interest Statement

The authors declare that the research was conducted in the absence of any commercial or financial relationships that could be construed as a potential conflict of interest.

## References

[B1] Akarid K. Arnoult D. Micic-Polianski J. Sif J. Estaquier J. Ameisen J. C. (2004). Leishmania major-mediated prevention of programmed cell death induction in infected macrophages is associated with the repression of mitochondrial release of cytochrome c. *J. Leukoc. Biol.* 76 95–103. 10.1189/jlb.1001877 15075349

[B2] Alcais A. Abel L. David C. Torrez M. E. Flandre P. Dedet J. P. (1997). Risk factors for onset of cutaneous and mucocutaneous leishmaniasis in Bolivia. *Am. J. Trop. Med. Hyg.* 57 79–84. 10.4269/ajtmh.1997.57.79 9242324

[B3] Andreyev A. Y. Kushnareva Y. E. Starkov A. A. (2005). Mitochondrial metabolism of reactive oxygen species. *Biochemistry* 70 200–214. 15807660 10.1007/s10541-005-0102-7

[B4] Azulay R. D. Azulay Junior D. R. (1995). Immune-clinical-pathologic spectrum of leishmaniasis. *Int. J. Dermatol.* 34 303–307. 10.1111/j.1365-4362.1995.tb03608.x 7607788

[B5] Bacellar O. Lessa H. Schriefer A. Machado P. Ribeiro de Jesus A. Dutra W. O. (2002). Up-regulation of Th1-type responses in mucosal leishmaniasis patients. *Infect. Immun.* 70 6734–6740. 10.1128/IAI.70.12.6734-6740.2002 12438348 PMC132996

[B6] Ben-Othman R. Guizani-Tabbane L. Dellagi K. (2008). Leishmania initially activates but subsequently down-regulates intracellular mitogen-activated protein kinases and nuclear factor-kappaB signaling in macrophages. *Mol. Immunol.* 45 3222–3229. 10.1016/j.molimm.2008.02.019 18406464

[B7] Bhardwaj N. Rosas L. E. Lafuse W. P. Satoskar A. R. (2005). Leishmania inhibits STAT1-mediated IFN-gamma signaling in macrophages: increased tyrosine phosphorylation of dominant negative STAT1beta by *Leishmania mexicana*. *Int. J. Parasitol.* 35 75–82. 10.1016/j.ijpara.2004.10.018 15619518

[B8] Bonifati V. (2007). Genetics of parkinsonism. *Parkinsonism Relat. Disord.* 13(Suppl. 3), S233–S241. 10.1016/S1353-8020(08)70008-7 18267242

[B9] Cabrera M. Shaw M. A. Sharples C. Williams H. Castes M. Convit J. (1995). Polymorphism in tumor necrosis factor genes associated with mucocutaneous leishmaniasis. *J. Exp. Med.* 182 1259–1264. 10.1084/jem.182.5.1259 7595196 PMC2192198

[B10] Calegari-Silva T. C. Pereira R. M. De-Melo L. D. Saraiva E. M. Soares D. C. Bellio M. (2009). NF-kappaB-mediated repression of iNOS expression in *Leishmania amazonensis* macrophage infection. *Immunol. Lett.* 127 19–26. 10.1016/j.imlet.2009.08.009 19712696

[B11] Carvalho E. M. Barral A. Costa J. M. Bittencourt A. Marsden P. (1994). Clinical and immunopathological aspects of disseminated cutaneous leishmaniasis. *Acta Trop.* 56 315–325. 10.1016/0001-706X(94)90103-1 8023755

[B12] Castellucci L. Cheng L. H. Araujo C. Guimaraes L. H. Lessa H. Machado P. (2005). Familial aggregation of mucosal leishmaniasis in northeast Brazil. *Am. J. Trop. Med. Hyg.* 73 69–73. 10.4269/ajtmh.2005.73.69 16014836

[B13] Castellucci L. Menezes E. Oliveira J. Magalhaes A. Guimaraes L. H. Lessa M. (2006). IL6 -174 G/C promoter polymorphism influences susceptibility to mucosal but not localized cutaneous leishmaniasis in Brazil. *J. Infect. Dis.* 194 519–527. 10.1086/505504 16845637

[B14] Castellucci L. C. Almeida L. F. Jamieson S. E. Fakiola M. Carvalho E. M. Blackwell J. M. (2014). Host genetic factors in American cutaneous leishmaniasis: a critical appraisal of studies conducted in an endemic area of Brazil. *Memorias Instit. Oswaldo Cruz* 109 279–288. 10.1590/0074-0276140028 24863979 PMC4131779

[B15] Charmoy M. Brunner-Agten S. Aebischer D. Auderset F. Launois P. Milon G. (2010). Neutrophil-derived CCL3 is essential for the rapid recruitment of dendritic cells to the site of Leishmania major inoculation in resistant mice. *PLoS Pathog.* 6:e1000755. 10.1371/journal.ppat.1000755 20140197 PMC2816696

[B16] Chaussabel D. Semnani R. T. McDowell M. A. Sacks D. Sher A. Nutman T. B. (2003). Unique gene expression profiles of human macrophages and dendritic cells to phylogenetically distinct parasites. *Blood* 102 672–681. 10.1182/blood-2002-10-3232 12663451

[B17] Chomova M. Racay P. (2010). Mitochondrial complex I in the network of known and unknown facts. *Gen. Physiol. Biophys.* 29 3–11. 10.4149/gpb_2010_01_3 20371875

[B18] Costa J. M. Marsden P. D. Llanos-Cuentas E. A. Netto E. M. Carvalho E. M. Barral A. (1986). Disseminated cutaneous leishmaniasis in a field clinic in Bahia, Brazil: a report of eight cases. *J. Trop. Med. Hyg.* 89 319–323. 3806749

[B19] Donovan M. J. Maciuba B. Z. Mahan C. E. McDowell M. A. (2009). Leishmania infection inhibits cycloheximide-induced macrophage apoptosis in a strain-dependent manner. *Exp. Parasitol.* 123 58–64. 10.1016/j.exppara.2009.05.012 19500578 PMC2744835

[B20] Engwerda C. R. Ato M. Kaye P. M. (2004). Macrophages, pathology and parasite persistence in experimental visceral leishmaniasis. *Trends Parasitol.* 20 524–530. 10.1016/j.pt.2004.08.009 15471704

[B21] Ettinger N. A. Wilson M. E. (2008). Macrophage and T-cell gene expression in a model of early infection with the protozoan *Leishmania chagasi*. *PLoS Negl. Trop. Dis.* 2:e252. 10.1371/journal.pntd.0000252 18575603 PMC2427198

[B22] Forget G. Gregory D. J. Olivier M. (2005). Proteasome-mediated degradation of STAT1alpha following infection of macrophages with *Leishmania donovani*. *J. Biol. Chem.* 280 30542–30549. 10.1074/jbc.M414126200 15983048

[B23] Garcia L. Kindt A. Bermudez H. Llanos-Cuentas A. De Doncker S. Arevalo J. (2004). Culture-independent species typing of neotropical *Leishmania* for clinical validation of a PCR-based assay targeting heat shock protein 70 genes. *J. Clin. Microbiol.* 42 2294–2297. 10.1128/JCM.42.5.2294-2297.2004 15131217 PMC404633

[B24] Gautier L. Cope L. Bolstad B. M. Irizarry R. A. (2004). affy–analysis of Affymetrix GeneChip data at the probe level. *Bioinformatics* 20 307–315. 10.1093/bioinformatics/btg405 14960456

[B25] Goncalves R. Zhang X. Cohen H. Debrabant A. Mosser D. M. (2011). Platelet activation attracts a subpopulation of effector monocytes to sites of *Leishmania* major infection. *J. Exp. Med.* 208 1253–1265. 10.1084/jem.20101751 21606505 PMC3173254

[B26] Gregory D. J. Sladek R. Olivier M. Matlashewski G. (2008). Comparison of the effects of Leishmania major or *Leishmania donovani* infection on macrophage gene expression. *Infect. Immun.* 76 1186–1192. 10.1128/IAI.01320-07 18086813 PMC2258831

[B27] Guimaraes L. H. Machado P. R. Lago E. L. Morgan D. J. Schriefer A. Bacellar O. (2009). Atypical manifestations of tegumentary leishmaniasis in a transmission area of *Leishmania braziliensis* in the state of Bahia, Brazil. *Trans. R. Soc. Trop. Med. Hyg.* 103 712–715. 10.1016/j.trstmh.2009.04.019 19481233 PMC2714265

[B28] Guimaraes L. H. Queiroz A. Silva J. A. Silva S. C. Magalhaes V. Lago E. L. (2016). Atypical manifestations of cutaneous Leishmaniasis in a region endemic for *Leishmania braziliensis*: clinical, immunological and parasitological aspects. *PLoS Negl. Trop. Dis.* 10:e0005100. 10.1371/journal.pntd.0005100 27906988 PMC5131895

[B29] Healy D. G. Falchi M. O’Sullivan S. S. Bonifati V. Durr A. Bressman S. (2008). Phenotype, genotype, and worldwide genetic penetrance of LRRK2-associated Parkinson’s disease: a case-control study. *Lancet Neurol.* 7 583–590. 10.1016/S1474-4422(08)70117-0 18539534 PMC2832754

[B30] Irizarry R. A. Hobbs B. Collin F. Beazer-Barclay Y. D. Antonellis K. J. Scherf U. (2003). Exploration, normalization, and summaries of high density oligonucleotide array probe level data. *Biostatistics* 4 249–264. 10.1093/biostatistics/4.2.249 12925520

[B31] Jin Z. El-Deiry W. S. (2005). Overview of cell death signaling pathways. *Cancer Biol. Ther.* 4 139–163. 10.4161/cbt.4.2.1508 15725726

[B32] Junghae M. Raynes J. G. (2002). Activation of p38 mitogen-activated protein kinase attenuates *Leishmania donovani* infection in macrophages. *Infect. Immun.* 70 5026–5035. 10.1128/IAI.70.9.5026-5035.2002 12183549 PMC128247

[B33] Kahl L. P. Byram J. E. David J. R. Comerford S. A. Von Lichtenberg F. (1991). *Leishmania (Viannia) braziliensis*: comparative pathology of golden hamsters infected with isolates from cutaneous and mucosal lesions of patients residing in Tres Bracos. Bahia, Brazil. *Am. J. Trop. Med. Hyg.* 44 218–232. 10.4269/ajtmh.1991.44.218 1849379

[B34] Kelley K. D. Miller K. R. Todd A. Kelley A. R. Tuttle R. Berberich S. J. (2010). YPEL3, a p53-regulated gene that induces cellular senescence. *Cancer Res.* 70 3566–3575. 10.1158/0008-5472.CAN-09-3219 20388804 PMC2862112

[B35] Kenny E. F. O’Neill L. A. (2008). Signalling adaptors used by Toll-like receptors: an update. *Cytokine* 43 342–349. 10.1016/j.cyto.2008.07.010 18706831

[B36] Lin T. K. Liou C. W. Chen S. D. Chuang Y. C. Tiao M. M. Wang P. W. (2009). Mitochondrial dysfunction and biogenesis in the pathogenesis of Parkinson’s disease. *Chang. Gung Med. J.* 32 589–599. 20035637

[B37] Lisi S. Sisto M. Acquafredda A. Spinelli R. Schiavone M. Mitolo V. (2005). Infection with *Leishmania infantum* Inhibits actinomycin D-induced apoptosis of human monocytic cell line U-937. *J. Eukaryot. Microbiol.* 52 211–217. 10.1111/j.1550-7408.2005.00026.x 15926996

[B38] Llanos-Cuentas E. A. Marsden P. D. Cuba C. C. Barreto A. C. Campos M. (1984). Possible risk factors in development of mucosal lesions in leishmaniasis. *Lancet* 2:295. 10.1016/S0140-6736(84)90346-5 6146853

[B39] Machado P. R. Rosa M. E. Costa D. Mignac M. Silva J. S. Schriefer A. (2011). Reappraisal of the immunopathogenesis of disseminated leishmaniasis: in situ and systemic immune response. *Trans. R. Soc. Trop. Med. Hyg.* 105 438–444. 10.1016/j.trstmh.2011.05.002 21723576 PMC3157292

[B40] Machado-Coelho G. L. Caiaffa W. T. Genaro O. Magalhaes P. A. Mayrink W. (2005). Risk factors for mucosal manifestation of American cutaneous leishmaniasis. *Trans. R. Soc. Trop. Med. Hyg.* 99 55–61. 10.1016/j.trstmh.2003.08.001 15550262

[B41] Martinez D. Y. Verdonck K. Kaye P. M. Adaui V. Polman K. Llanos-Cuentas A. (2018). Tegumentary leishmaniasis and coinfections other than HIV. *PLoS Negl. Trop. Dis.* 12:e0006125. 10.1371/journal.pntd.0006125 29494584 PMC5832191

[B42] Matlashewski G. Buates S. (2001). General suppression of macrophage gene expression during *Leishmania donovani* infection. *J. Immunol.* 166 3416–3422. 10.4049/jimmunol.166.5.3416 11207299

[B43] Matte C. Descoteaux A. (2010). *Leishmania donovani* amastigotes impair gamma interferon-induced STAT1alpha nuclear translocation by blocking the interaction between STAT1alpha and importin-alpha5. *Infect. Immun.* 78 3736–3743. 10.1128/IAI.00046-10 20566692 PMC2937469

[B44] Meireles C. B. Maia L. C. Soares G. C. Teodoro I. P. P. Gadelha M. da Silva C. G. L. (2017). Atypical presentations of cutaneous leishmaniasis: a systematic review. *Acta Trop.* 172 240–254. 10.1016/j.actatropica.2017.05.022 28526427

[B45] Montalvo A. M. Fraga J. Monzote L. Montano I. De Doncker S. Dujardin J. C. (2010). Heat-shock protein 70 PCR-RFLP: a universal simple tool for *Leishmania* species discrimination in the New and Old World. *Parasitology* 137 1159–1168. 10.1017/S0031182010000089 20441679

[B46] Moore K. J. Labrecque S. Matlashewski G. (1993). Alteration of *Leishmania donovani* infection levels by selective impairment of macrophage signal transduction. *J. Immunol.* 150 4457–4465. 8482844

[B47] Moore K. J. Matlashewski G. (1994). Intracellular infection by Leishmania donovani inhibits macrophage apoptosis. *J. Immunol.* 152 2930–2937. 8144893

[B48] Morgan D. J. Guimaraes L. H. Machado P. R. D’Oliveira A. Jr. Almeida R. P. Lago E. L. (2007). Cutaneous leishmaniasis during pregnancy: exuberant lesions and potential fetal complications. *Clin. Infect. Dis.* 45 478–482. 10.1086/520017 17638198

[B49] Murray H. W. Berman J. D. Davies C. R. Saravia N. G. (2005). Advances in leishmaniasis. *Lancet* 366 1561–1577. 10.1016/S0140-6736(05)67629-5 16257344

[B50] Namdarghanbari M. Wobig W. Krezoski S. Tabatabai N. M. Petering D. H. (2011). Mammalian metallothionein in toxicology, cancer, and cancer chemotherapy. *J. Biol. Inorg. Chem.* 16 1087–1101. 10.1007/s00775-011-0823-6 21822976

[B51] Oeckinghaus A. Hayden M. S. Ghosh S. (2011). Crosstalk in NF-kappaB signaling pathways. *Nat. Immunol.* 12 695–708. 10.1038/ni.2065 21772278

[B52] Paisan-Ruiz C. Jain S. Evans E. W. Gilks W. P. Simon J. van der Brug M. (2004). Cloning of the gene containing mutations that cause PARK8-linked Parkinson’s disease. *Neuron* 44 595–600. 10.1016/j.neuron.2004.10.023 15541308

[B53] Queiroz A. Sousa R. Heine C. Cardoso M. Guimaraes L. H. Machado P. R. (2012). Association between an emerging disseminated form of leishmaniasis and *Leishmania (Viannia) braziliensis* strain polymorphisms. *J. Clin. Microbiol.* 50 4028–4034. 10.1128/JCM.02064-12 23035200 PMC3503016

[B54] Ribeiro-Gomes F. L. Peters N. C. Debrabant A. Sacks D. L. (2012). Efficient capture of infected neutrophils by dendritic cells in the skin inhibits the early anti-leishmania response. *PLoS Pathog.* 8:e1002536. 10.1371/journal.ppat.1002536 22359507 PMC3280984

[B55] Rodriguez N. E. Chang H. K. Wilson M. E. (2004). Novel program of macrophage gene expression induced by phagocytosis of *Leishmania chagasi*. *Infect. Immun.* 72 2111–2122. 10.1128/IAI.72.4.2111-2122.2004 15039333 PMC375188

[B56] Rouault T. A. Tong W. H. (2005). Iron-sulphur cluster biogenesis and mitochondrial iron homeostasis. *Nat. Rev. Mol. Cell Biol.* 6 345–351. 10.1038/nrm1620 15803140

[B57] Ruhland A. Leal N. Kima P. E. (2007). Leishmania promastigotes activate PI3K/Akt signalling to confer host cell resistance to apoptosis. *Cell Microbiol.* 9 84–96. 10.1111/j.1462-5822.2006.00769.x 16889626

[B58] Salhi A. Rodrigues V. Jr. Santoro F. Dessein H. Romano A. Castellano L. R. (2008). Immunological and genetic evidence for a crucial role of IL-10 in cutaneous lesions in humans infected with *Leishmania braziliensis*. *J. Immunol.* 180 6139–6148. 10.4049/jimmunol.180.9.6139 18424735

[B59] Saravia N. G. Segura I. Holguin A. F. Santrich C. Valderrama L. Ocampo C. (1998). Epidemiologic, genetic, and clinical associations among phenotypically distinct populations of *Leishmania (Viannia)* in Colombia. *Am J. Trop. Med. Hyg.* 59 86–94. 10.4269/ajtmh.1998.59.86 9684634

[B60] Saravia N. G. Weigle K. Navas C. Segura I. Valderrama L. Valencia A. Z. (2002). Heterogeneity, geographic distribution, and pathogenicity of serodemes of *Leishmania viannia* in Colombia. *Am. J. Trop. Med. Hyg.* 66 738–744. 10.4269/ajtmh.2002.66.738 12224583

[B61] Schriefer A. Guimaraes L. H. Machado P. R. Lessa M. Lessa H. A. Lago E. (2009). Geographic clustering of leishmaniasis in northeastern Brazil. *Emerg. Infect. Dis.* 15 871–876. 10.3201/eid1506.080406 19523284 PMC2727348

[B62] Schriefer A. Schriefer A. L. Goes-Neto A. Guimaraes L. H. Carvalho L. P. Almeida R. P. (2004). Multiclonal *Leishmania braziliensis* population structure and its clinical implication in a region of endemicity for American tegumentary leishmaniasis. *Infect. Immun.* 72 508–514. 10.1128/IAI.72.1.508-514.2004 14688132 PMC343999

[B63] Tricarico C. Pinzani P. Bianchi S. Paglierani M. Distante V. Pazzagli M. (2002). Quantitative real-time reverse transcription polymerase chain reaction: normalization to rRNA or single housekeeping genes is inappropriate for human tissue biopsies. *Anal. Biochem.* 309 293–300. 10.1016/S0003-2697(02)00311-1 12413463

[B64] Turetz M. L. Machado P. R. Ko A. I. Alves F. Bittencourt A. Almeida R. P. (2002). Disseminated leishmaniasis: a new and emerging form of leishmaniasis observed in northeastern Brazil. *J. Infect. Dis.* 186 1829–1834. 10.1086/345772 12447770

[B65] Venanzoni M. C. Robinson L. R. Hodge D. R. Kola I. Seth A. (1996). ETS1 and ETS2 in p53 regulation: spatial separation of ETS binding sites (EBS) modulate protein: DNA interaction. *Oncogene* 12 1199–1204. 8649821

[B66] Weirather J. L. Jeronimo S. M. Gautam S. Sundar S. Kang M. Kurtz M. A. (2011). Serial quantitative PCR assay for detection, species-discrimination and quantification of *Leishmania* spp. in human samples. *J. Clin. Microbiol.* 49 3892–3904. 10.1128/JCM.r00764-11 22042830 PMC3209110

[B67] Wong E. Cuervo A. M. (2010). Integration of clearance mechanisms: the proteasome and autophagy. *Cold Spring Harb. Perspect. Biol.* 2:a006734. 10.1101/cshperspect.a006734 21068151 PMC2982176

[B68] Zhang F. R. Huang W. Chen S. M. Sun L. D. Liu H. Li Y. (2009). Genomewide association study of leprosy. *N. Engl. J. Med.* 361 2609–2618. 10.1056/NEJMoa0903753 20018961

[B69] Zimprich A. Biskup S. Leitner P. Lichtner P. Farrer M. Lincoln S. (2004). Mutations in LRRK2 cause autosomal-dominant parkinsonism with pleomorphic pathology. *Neuron* 44 601–607. 10.1016/j.neuron.2004.11.005 15541309

