# Supplementary material for: Early Suppression of Macrophage Gene Expression by Leishmania braziliensis
Source: Front Microbiol. 2018 Oct 15;9:2464. doi: 10.3389/fmicb.2018.02464 (PMC6196312; doi:10.3389/fmicb.2018.02464)
Supplement: Supplementary file 1 [file Table_1.DOCX]

**Supplemental Table 1.** Extensive list of monocytes derived macrophages (MDM) genes significantly affected by infection with any *Leishmania braziliensis* isolate employed in the DNA micro array experiments. Only genes significantly affected in MDM of all four human volunteers were considered. One isolate per *L. braziliensis* clade (described in reference 10) was tested in the experiments. Criteria for significance and experimental details are described in the methods section.

| **Gene Title** | **Gene Symbol** | **p-value**  **(A vs. M)** | **GFoldChange**  **(A vs. M)** | **p-value**  **(B vs. M)** | **GFoldChange**  **(B vs. M)** | **p-value**  **(C vs. M)** | **GFoldChange**  **(C vs. M)** |
| --- | --- | --- | --- | --- | --- | --- | --- |
| **metallothionein 1M** | **MT1M** | **9.05665e-005** | **7.65333** | **0.000215829** | **6.26874** | **2.07091e-005** | **11.0482** |
| **heat shock 70kDa protein 1A /// heat shock 70kDa protein 1B** | **HSPA1A /// HSPA1B** | **7.71182e-005** | **5.76296** | **0.000256787** | **4.5629** | **5.82824e-005** | **6.10136** |
| **egl nine homolog 3 (C. elegans)** | **EGLN3** | **4.3673e-005** | **4.34749** | **0.000454606** | **3.03849** | **2.50548e-005** | **4.77388** |
| **ankyrin repeat domain 37** | **ANKRD37** | **8.3098e-005** | **2.51597** | **0.000497112** | **2.10065** | **9.83706e-005** | **2.47133** |
| **solute carrier family 2 (facilitated glucose transporter), member 3** | **SLC2A3** | **4.58304e-005** | **2.19797** | **0.000139352** | **1.99891** | **9.66865e-005** | **2.06067** |
| **Transcribed locus** | **---** | **3.73118e-005** | **2.16609** | **0.000196207** | **1.89186** | **1.9217e-005** | **2.29686** |
| **UDP-GlcNAc:betaGal beta-1,3-N-acetylglucosaminyltransferase 5** | **B3GNT5** | **4.70807e-005** | **2.03626** | **0.000284925** | **1.77746** | **7.06314e-005** | **1.97203** |
| **ATPase, Ca++ transporting, plasma membrane 2** | **ATP2B2** | **1.81113e-005** | **1.92387** | **0.000117866** | **1.69849** | **9.55738e-005** | **1.72099** |
| **mitogen-activated protein kinase kinase 3** | **MAP2K3** | **6.23796e-005** | **1.88078** | **0.000128993** | **1.78708** | **6.32058e-005** | **1.879** |
| **hypothetical protein FLJ35773** | **FLJ35773** | **4.70527e-006** | **1.78446** | **0.000358607** | **1.42599** | **2.29754e-005** | **1.63062** |
| **CDNA FLJ35910 fis, clone TESTI2009987** | **---** | **2.59085e-005** | **1.70852** | **0.000118163** | **1.56958** | **1.6202e-005** | **1.75729** |
| **ELL associated factor 1** | **EAF1** | **4.40119e-005** | **1.69947** | **0.000130805** | **1.59643** | **3.09432e-005** | **1.73598** |
| **kelch-like 21 (Drosophila)** | **KLHL21** | **5.79205e-005** | **1.68458** | **0.000134488** | **1.60473** | **3.97511e-005** | **1.72312** |
| **histone cluster 1, H3g** | **HIST1H3G** | **6.30657e-005** | **1.56921** | **0.00211651** | **1.33376** | **2.3893e-005** | **1.6524** |
| **guanine nucleotide binding protein (G protein), alpha 13** | **GNA13** | **2.92387e-005** | **1.48672** | **0.000405862** | **1.33772** | **3.17396e-005** | **1.4814** |
| **lactate dehydrogenase A** | **LDHA** | **6.76702e-005** | **1.38679** | **0.00104552** | **1.26212** | **4.45806e-005** | **1.40895** |
| **ribophorin II** | **RPN2** | **2.01053e-005** | **-1.351** | **0.00108586** | **-1.20451** | **8.04443e-005** | **-1.29376** |
| **ubiquitin specific peptidase 19** | **USP19** | **4.53415e-005** | **-1.464** | **0.000105496** | **-1.41339** | **3.88823e-005** | **-1.47374** |
| **tumor necrosis factor (ligand) superfamily, member 13b** | **TNFSF13B** | **2.94104e-005** | **-1.878** | **0.000196226** | **-1.6591** | **6.1439e-005** | **-1.78639** |
| **NADH dehydrogenase (ubiquinone) 1 alpha subcomplex, 11, 14.7kDa** | **NDUFA11** | **1.61998e-005** | **-1.3888** | **0.000285867** | **-1.26625** | **7.42091e-006** | **-1.42904** |
| **MRNA; cDNA DKFZp667B1718 (from clone DKFZp667B1718)** | **---** | **1.35123e-005** | **-1.5266** | **0.000483312** | **-1.32188** | **4.24368e-006** | **-1.61303** |
| **Spleen tyrosine kinase** | **SYK** | **2.07859e-005** | **-2.0477** | **0.000615216** | **-1.61622** | **1.15928e-005** | **-2.14553** |
| **MRNA; cDNA DKFZp762M127 (from clone DKFZp762M127)** | **---** | **2.33933e-005** | **-2.3263** | **0.000387207** | **-1.83683** | **6.00974e-005** | **-2.13796** |
| **tumor necrosis factor, alpha-induced protein 8-like 2** | **TNFAIP8L2** | **1.93838e-005** | **-3.0688** | **0.000402709** | **-2.19724** | **7.89343e-005** | **-2.60708** |
| **parvin, gamma** | **PARVG** | **6.48682e-005** | **-1.28507** | **0.0141516** | **-1.12447** | **6.51271e-005** | **-1.28492** |
| **chromosome 20 open reading frame 3** | **C20orf3** | **2.99904e-005** | **-1.34425** | **0.00132457** | **-1.20443** | **6.34103e-005** | **-1.31255** |
| **ATP synthase, H+ transporting, mitochondrial F1 complex, alpha subunit 1, cardia** | **ATP5A1** | **3.90534e-005** | **-1.34502** | **0.000825001** | **-1.22723** | **7.08968e-005** | **-1.31923** |
| **SLAM family member 8** | **SLAMF8** | **4.2671e-005** | **-1.35095** | **0.000160089** | **-1.2945** | **2.55947e-005** | **-1.3749** |
| **asparaginyl-tRNA synthetase** | **NARS** | **2.99407e-005** | **-1.35357** | **0.000595783** | **-1.23612** | **6.71655e-005** | **-1.31843** |
| **aldo-keto reductase family 7, member A2 (aflatoxin aldehyde reductase)** | **AKR7A2** | **8.32481e-005** | **-1.35732** | **0.0398651** | **-1.12489** | **9.92101e-005** | **-1.34898** |
| **signal transducer and activator of transcription 1, 91kDa** | **STAT1** | **6.06884e-005** | **-1.39649** | **0.00179476** | **-1.24319** | **3.96141e-005** | **-1.41969** |
| **NADH dehydrogenase (ubiquinone) 1, subcomplex unknown, 1, 6kDa** | **NDUFC1** | **3.5465e-005** | **-1.42655** | **0.000959163** | **-1.26858** | **4.37207e-005** | **-1.41487** |
| **vesicle-associated membrane protein 8 (endobrevin)** | **VAMP8** | **9.09309e-005** | **-1.43111** | **0.00411726** | **-1.24106** | **9.09612e-005** | **-1.43109** |
| **dihydrouridine synthase 1-like (S. cerevisiae)** | **DUS1L** | **4.33248e-005** | **-1.43287** | **0.000143127** | **-1.36798** | **3.7523e-005** | **-1.44126** |
| **protein tyrosine phosphatase-like A domain containing 1 /// similar to butyrate-** | **LOC732402 /// PTPLAD1** | **1.50584e-006** | **-1.44471** | **0.00012049** | **-1.25731** | **1.23186e-006** | **-1.45567** |
| **copper metabolism (Murr1) domain containing 1** | **COMMD1** | **1.54559e-005** | **-1.44488** | **0.000110316** | **-1.34334** | **2.15851e-005** | **-1.42606** |
| **CDNA FLJ25645 fis, clone SYN00113** | **---** | **3.38499e-005** | **-1.46603** | **0.00787359** | **-1.20328** | **6.99792e-005** | **-1.42254** |
| **JTV1 gene** | **JTV1** | **2.06814e-005** | **-1.46753** | **0.00167352** | **-1.25107** | **5.11344e-005** | **-1.41468** |
| **Wiskott-Aldrich syndrome (eczema-thrombocytopenia)** | **WAS** | **3.39741e-005** | **-1.46916** | **0.00048831** | **-1.3235** | **5.23313e-005** | **-1.44266** |
| **phosphopantothenoylcysteine synthetase** | **PPCS** | **1.18458e-005** | **-1.48117** | **0.000403188** | **-1.29884** | **6.66448e-005** | **-1.38344** |
| **WD repeat domain, phosphoinositide interacting 2** | **WIPI2** | **5.21525e-006** | **-1.49445** | **0.000548494** | **-1.26579** | **4.08636e-005** | **-1.37946** |
| **exportin 6** | **XPO6** | **7.73493e-005** | **-1.50949** | **0.000158596** | **-1.46008** | **3.54427e-005** | **-1.56803** |
| **ethylmalonic encephalopathy 1** | **ETHE1** | **4.59066e-005** | **-1.51479** | **0.0124421** | **-1.21093** | **6.03737e-005** | **-1.4957** |
| **transcription factor A, mitochondrial** | **TFAM** | **3.42623e-005** | **-1.53052** | **0.00349217** | **-1.26557** | **8.02956e-005** | **-1.47178** |
| **NADH dehydrogenase (ubiquinone) 1 beta subcomplex, 10, 22kDa** | **NDUFB10** | **9.64108e-006** | **-1.53102** | **0.000432758** | **-1.31634** | **5.43291e-005** | **-1.42246** |
| **acetyl-Coenzyme A acyltransferase 1 (peroxisomal 3-oxoacyl-Coenzyme A thiolase)** | **ACAA1** | **5.73792e-005** | **-1.53364** | **0.00201454** | **-1.31308** | **7.1888e-005** | **-1.51705** |
| **chromosome 11 open reading frame2** | **C11orf2** | **9.92579e-005** | **-1.53369** | **0.00182052** | **-1.34236** | **5.10956e-005** | **-1.58655** |
| **GLE1 RNA export mediator homolog (yeast)** | **GLE1** | **3.06178e-006** | **-1.53885** | **0.00157723** | **-1.23043** | **2.2516e-005** | **-1.41744** |
| **mitochondrial ribosomal protein S35** | **MRPS35** | **1.0742e-005** | **-1.54635** | **0.00014928** | **-1.38319** | **1.28405e-005** | **-1.53365** |
| **glutaminyl-tRNA synthetase** | **QARS** | **5.27321e-005** | **-1.55011** | **0.000712859** | **-1.37624** | **3.24851e-005** | **-1.58849** |
| **nuclear transcription factor Y, beta** | **NFYB** | **7.47872e-005** | **-1.57959** | **0.000393017** | **-1.45411** | **3.10154e-005** | **-1.65682** |
| **zinc finger, MYND-type containing 19** | **ZMYND19** | **1.81221e-005** | **-1.60114** | **0.00012102** | **-1.46217** | **2.14031e-005** | **-1.58766** |
| **unc-51-like kinase 2 (C. elegans)** | **ULK2** | **1.03045e-005** | **-1.60176** | **0.000876786** | **-1.32198** | **4.23413e-005** | **-1.49738** |
| **FtsJ homolog 2 (E. coli)** | **FTSJ2** | **7.93183e-005** | **-1.63886** | **0.000904592** | **-1.44093** | **8.97319e-005** | **-1.62742** |
| **PHD finger protein 12** | **PHF12** | **3.77508e-005** | **-1.64176** | **0.000476147** | **-1.44292** | **2.83116e-005** | **-1.66839** |
| **glutaredoxin 5** | **GLRX5** | **4.95052e-006** | **-1.66653** | **0.00101131** | **-1.31618** | **6.73527e-006** | **-1.63972** |
| **DEAH (Asp-Glu-Ala-His) box polypeptide 35** | **DHX35** | **8.25076e-005** | **-1.67594** | **0.00282289** | **-1.38559** | **4.35773e-005** | **-1.74251** |
| **N-acyl-phosphatidylethanolamine-hydrolyzing phospholipase D** | **NAPE-PLD** | **7.73281e-005** | **-1.68635** | **0.00411418** | **-1.36211** | **9.34508e-005** | **-1.66735** |
| **chromobox homolog 6** | **CBX6** | **6.80794e-006** | **-1.69285** | **0.000109039** | **-1.47312** | **2.10964e-005** | **-1.59414** |
| **DNA (cytosine-5-)-methyltransferase 1** | **DNMT1** | **2.52063e-005** | **-1.70684** | **0.000348488** | **-1.48201** | **1.67912e-005** | **-1.74869** |
| **family with sequence similarity 120C** | **FAM120C** | **2.38306e-005** | **-1.72892** | **0.000209182** | **-1.53179** | **2.27577e-005** | **-1.73372** |
| **programmed cell death 6** | **PDCD6** | **5.4576e-005** | **-1.73748** | **0.000383085** | **-1.54935** | **7.23679e-005** | **-1.70732** |
| **chromosome 6 open reading frame 70** | **C6orf70** | **6.02543e-005** | **-1.73801** | **0.000409669** | **-1.5516** | **2.67286e-005** | **-1.83199** |
| **Transcribed locus** | **---** | **2.38125e-005** | **-1.73853** | **0.000162962** | **-1.55877** | **6.47497e-005** | **-1.63965** |
| **mitogen-activated protein kinase 14** | **MAPK14** | **2.07957e-005** | **-1.74817** | **0.000172754** | **-1.55062** | **5.78548e-005** | **-1.64614** |
| **tyrosine kinase 2** | **TYK2** | **2.32594e-005** | **-1.75948** | **0.000160678** | **-1.5732** | **3.71196e-005** | **-1.71012** |
| **NADH dehydrogenase (ubiquinone) flavoprotein 3, 10kDa** | **NDUFV3** | **4.55964e-006** | **-1.76472** | **0.000170585** | **-1.46239** | **1.11309e-005** | **-1.67694** |
| **solute carrier family 1 (neutral amino acid transporter), member 5** | **SLC1A5** | **9.32323e-005** | **-1.78486** | **0.000229748** | **-1.68255** | **1.03953e-005** | **-2.09405** |
| **phosphopantothenoylcysteine decarboxylase** | **PPCDC** | **1.54064e-005** | **-1.78613** | **0.00101607** | **-1.42154** | **1.14319e-005** | **-1.82021** |
| **Transcribed locus** | **---** | **6.33453e-006** | **-1.79663** | **0.000240722** | **-1.47576** | **4.27551e-005** | **-1.61024** |
| **Transcribed locus** | **---** | **1.6135e-005** | **-1.82042** | **0.000242616** | **-1.55143** | **6.09891e-005** | **-1.67687** |
| **acidic (leucine-rich) nuclear phosphoprotein 32 family, member A** | **ANP32A** | **8.24914e-006** | **-1.83266** | **0.000981883** | **-1.41228** | **1.7951e-005** | **-1.74601** |
| **nuclear factor (erythroid-derived 2)-like 1** | **NFE2L1** | **5.97941e-006** | **-1.83403** | **0.00020724** | **-1.50352** | **5.50428e-006** | **-1.84375** |
| **butyrophilin, subfamily 3, member A3 /// butyrophilin, subfamily 3, member A2** | **BTN3A2 /// BTN3A3** | **1.74597e-005** | **-1.83673** | **0.000740974** | **-1.47687** | **4.20808e-005** | **-1.73637** |
| **ubiquitin-like 7 (bone marrow stromal cell-derived)** | **UBL7** | **9.97034e-005** | **-1.83986** | **0.000787864** | **-1.60259** | **6.43391e-005** | **-1.89897** |
| **hypothetical LOC145853** | **LOC145853** | **4.81646e-005** | **-1.84302** | **0.000165398** | **-1.69855** | **3.53321e-005** | **-1.88326** |
| **ceroid-lipofuscinosis, neuronal 6, late infantile, variant** | **CLN6** | **6.13306e-005** | **-1.85876** | **0.00883549** | **-1.36917** | **3.36566e-005** | **-1.94103** |
| **calcium/calmodulin-dependent protein kinase kinase 2, beta** | **CAMKK2** | **8.90843e-006** | **-1.86156** | **0.000102443** | **-1.60629** | **1.61629e-005** | **-1.7914** |
| **arylsulfatase D** | **ARSD** | **3.60496e-005** | **-1.87508** | **0.000573239** | **-1.57082** | **5.39742e-005** | **-1.82352** |
| **myosin IF** | **MYO1F** | **6.70969e-006** | **-1.88422** | **0.00100481** | **-1.42208** | **3.80632e-005** | **-1.69047** |
| **chromosome 12 open reading frame 32** | **C12orf32** | **7.19813e-006** | **-1.88459** | **0.000370086** | **-1.49842** | **1.28698e-005** | **-1.81434** |
| **arylsulfatase D** | **ARSD** | **4.21961e-005** | **-1.89828** | **0.000302636** | **-1.6624** | **1.20051e-005** | **-2.0866** |
| **Acidic (leucine-rich) nuclear phosphoprotein 32 family, member A** | **ANP32A** | **1.51039e-005** | **-1.90951** | **0.0019132** | **-1.43062** | **2.71918e-005** | **-1.83433** |
| **FYVE, RhoGEF and PH domain containing 3** | **FGD3** | **5.58999e-005** | **-1.93562** | **0.000132953** | **-1.81725** | **1.0305e-005** | **-2.21488** |
| **cytochrome b-561 domain containing 2** | **CYB561D2** | **1.03949e-005** | **-1.93695** | **0.000462964** | **-1.53167** | **1.32425e-005** | **-1.90447** |
| **GRAM domain containing 1A** | **GRAMD1A** | **6.44775e-005** | **-1.95228** | **0.00010758** | **-1.87861** | **9.23776e-005** | **-1.89998** |
| **ADP-ribosylation factor-like 3** | **ARL3** | **7.69468e-005** | **-1.95372** | **0.00498315** | **-1.46795** | **6.51051e-005** | **-1.97923** |
| **Transcribed locus** | **---** | **3.85569e-005** | **-1.95808** | **0.000117997** | **-1.80603** | **3.82548e-005** | **-1.95924** |
| **CDNA: FLJ21556 fis, clone COL06353** | **---** | **9.32513e-006** | **-1.96166** | **0.000284119** | **-1.57899** | **1.24131e-005** | **-1.92231** |
| **Impact homolog (mouse)** | **IMPACT** | **2.5687e-006** | **-1.96195** | **0.000116378** | **-1.5601** | **1.25247e-005** | **-1.7697** |
| **mitochondrial ribosomal protein L16** | **MRPL16** | **2.17496e-005** | **-2.00818** | **0.000230342** | **-1.70127** | **8.89629e-005** | **-1.81317** |
| **neighbor of BRCA1 gene 1 /// similar to neighbor of BRCA1 gene 1** | **LOC727732 /// NBR1** | **1.3418e-005** | **-2.04999** | **0.000470828** | **-1.6076** | **4.93785e-005** | **-1.86253** |
| **chromosome X open reading frame 9** | **CXorf9** | **3.75632e-005** | **-2.05513** | **0.000382454** | **-1.72898** | **4.89203e-005** | **-2.01233** |
| **hect domain and RLD 2** | **HERC2** | **8.12817e-006** | **-2.07788** | **0.000111837** | **-1.7276** | **4.18133e-005** | **-1.84413** |
| **basic helix-loop-helix domain containing, class B, 3** | **BHLHB3** | **5.53199e-006** | **-2.10733** | **0.000812984** | **-1.51861** | **2.02683e-005** | **-1.91351** |
| **ras homolog gene family, member T1** | **RHOT1** | **1.39536e-005** | **-2.11063** | **0.000150885** | **-1.76999** | **4.86364e-005** | **-1.91753** |
| **tubulin tyrosine ligase-like family, member 12** | **TTLL12** | **3.7032e-005** | **-2.13891** | **0.000505158** | **-1.74517** | **5.84716e-005** | **-2.05889** |
| **acyl-Coenzyme A oxidase 1, palmitoyl** | **ACOX1** | **3.06187e-005** | **-2.16515** | **0.000196166** | **-1.86521** | **8.96939e-005** | **-1.98167** |
| **Fucokinase** | **FUK** | **3.06518e-005** | **-2.16788** | **0.000293487** | **-1.81189** | **2.39263e-005** | **-2.2148** |
| **metallophosphoesterase 1** | **MPPE1** | **9.19346e-006** | **-2.20327** | **0.000365551** | **-1.67891** | **6.56193e-005** | **-1.88969** |
| **Pyridoxamine 5'-phosphate oxidase** | **PNPO** | **2.03842e-005** | **-2.24443** | **0.00100558** | **-1.65877** | **2.84807e-005** | **-2.17999** |
| **intestinal cell (MAK-like) kinase** | **ICK** | **7.68298e-005** | **-2.27541** | **0.000600357** | **-1.89571** | **3.15881e-005** | **-2.4818** |
| **hydrogen voltage-gated channel 1** | **HVCN1** | **3.44104e-005** | **-2.30596** | **0.000551363** | **-1.82233** | **2.98031e-005** | **-2.33714** |
| **HEAT repeat containing 6** | **HEATR6** | **1.1215e-005** | **-2.32719** | **0.00023143** | **-1.82017** | **8.81462e-005** | **-1.95782** |
| **family with sequence similarity 60, member A** | **FAM60A** | **2.49509e-005** | **-2.33723** | **0.000837937** | **-1.74524** | **9.62711e-005** | **-2.07269** |
| **Full-length cDNA clone CS0DI051YA02 of Placenta Cot 25-normalized of Homo sapien** | **---** | **1.68826e-005** | **-2.37696** | **0.000806879** | **-1.72809** | **3.71803e-005** | **-2.21193** |
| **RNA pseudouridylate synthase domain containing 3** | **RPUSD3** | **7.12864e-006** | **-2.38011** | **0.000118291** | **-1.88725** | **5.03332e-005** | **-2.0155** |
| **poliovirus receptor** | **PVR** | **1.57942e-005** | **-2.39641** | **0.00129582** | **-1.67175** | **3.54072e-005** | **-2.22537** |
| **similar to AT rich interactive domain 1B (SWI1-like) isoform 1** | **LOC729446** | **7.96495e-005** | **-2.41825** | **0.000153824** | **-2.26462** | **7.86968e-005** | **-2.42122** |
| **vacuolar protein sorting 45 homolog (S. cerevisiae)** | **VPS45** | **2.78004e-005** | **-2.44519** | **0.00113839** | **-1.76707** | **6.47031e-005** | **-2.25522** |
| **iron-sulfur cluster assembly 2 homolog (S. cerevisiae)** | **ISCA2** | **1.49189e-005** | **-2.45283** | **0.000379011** | **-1.85338** | **2.9555e-005** | **-2.29954** |
| **toll-like receptor 8** | **TLR8** | **9.15004e-005** | **-2.51452** | **0.0017291** | **-1.88416** | **8.18538e-005** | **-2.54483** |
| **Utrophin** | **UTRN** | **1.65683e-005** | **-2.55459** | **0.000196348** | **-2.02903** | **1.68559e-005** | **-2.55013** |
| **poly (ADP-ribose) polymerase family, member 2** | **PARP2** | **1.84445e-005** | **-2.61825** | **0.000294398** | **-2.01181** | **6.59657e-005** | **-2.30516** |
| **mutS homolog 5 (E. coli) /// chromosome 6 open reading frame 26** | **C6orf26 /// MSH5** | **3.32914e-005** | **-2.85401** | **0.000333148** | **-2.22613** | **3.71594e-005** | **-2.81784** |
| **yippee-like 3 (Drosophila)** | **YPEL3** | **1.10105e-005** | **-2.86553** | **0.000141583** | **-2.20445** | **2.17379e-005** | **-2.65958** |
| **Butyrophilin, subfamily 3, member A3** | **BTN3A3** | **3.40577e-005** | **-2.86672** | **0.000359784** | **-2.22098** | **3.82702e-005** | **-2.828** |
| **family with sequence similarity 105, member A** | **FAM105A** | **5.4212e-005** | **-3.20008** | **0.000362746** | **-2.52831** | **1.84386e-005** | **-3.70979** |
| **solute carrier family 25 (carnitine/acylcarnitine translocase), member 20** | **SLC25A20** | **3.58193e-006** | **-3.99036** | **0.000289153** | **-2.33214** | **4.9436e-005** | **-2.83788** |
| **hypermethylated in cancer 1** | **HIC1** | **5.26337e-005** | **3.61289** | **0.000501033** | **2.66378** | **0.00023281** | **2.94068** |
| **chromosome 17 open reading frame 69** | **C17orf69** | **9.6193e-005** | **3.35194** | **0.00145381** | **2.35533** | **0.000238987** | **2.95947** |
| **hCG2040376** | **hCG_2040376** | **5.51076e-005** | **2.96197** | **0.000711266** | **2.21515** | **0.000145837** | **2.63743** |
| **splA/ryanodine receptor domain and SOCS box containing 1** | **SPSB1** | **7.87991e-005** | **2.41513** | **0.000339641** | **2.09583** | **0.000157297** | **2.25473** |
| **v-ets erythroblastosis virus E26 oncogene homolog 2 (avian)** | **ETS2** | **4.80848e-005** | **2.36415** | **0.000607089** | **1.88442** | **0.000184372** | **2.08701** |
| **potassium inwardly-rectifying channel, subfamily J, member 5** | **KCNJ5** | **9.32105e-005** | **1.98743** | **0.00819023** | **1.44805** | **0.025173** | **1.34702** |
| **period homolog 1 (Drosophila)** | **PER1** | **2.0048e-005** | **1.90495** | **0.00155422** | **1.46088** | **0.000184109** | **1.64911** |
| **solute carrier family 2 (facilitated glucose transporter), member 3** | **SLC2A3** | **9.94184e-005** | **1.78676** | **0.000456571** | **1.61927** | **0.00049069** | **1.6121** |
| **chromosome 20 open reading frame 59** | **C20orf59** | **8.22563e-005** | **1.68884** | **0.141187** | **1.14702** | **0.0130089** | **1.29171** |
| **ATPase, class II, type 9B** | **ATP9B** | **7.97566e-005** | **1.63673** | **0.0248039** | **1.23434** | **0.00908134** | **1.2919** |
| **ubiquitin specific peptidase 12** | **USP12** | **9.3778e-005** | **1.56305** | **0.000972177** | **1.3958** | **0.000104449** | **1.55435** |
| **branched chain aminotransferase 2, mitochondrial** | **BCAT2** | **2.28808e-005** | **1.55749** | **0.0093038** | **1.22058** | **0.000131641** | **1.43792** |
| **hCG2007354** | **hCG_2007354** | **6.1526e-005** | **1.53607** | **0.051236** | **1.16172** | **0.00023025** | **1.44395** |
| **potassium channel, subfamily T, member 1** | **KCNT1** | **1.34372e-005** | **1.52994** | **0.00491259** | **1.22292** | **0.00278337** | **1.24599** |
| **ubiquitin associated protein 1** | **UBAP1** | **3.48504e-005** | **1.52822** | **0.000293469** | **1.39161** | **0.000225023** | **1.40697** |
| **Transcribed locus** | **---** | **3.90957e-005** | **1.50633** | **0.0048404** | **1.24418** | **0.0046664** | **1.24579** |
| **CASP2 and RIPK1 domain containing adaptor with death domain** | **CRADD** | **8.63857e-005** | **1.49973** | **0.00663452** | **1.25172** | **0.0126532** | **1.22156** |
| **epithelial membrane protein 1** | **EMP1** | **8.93161e-005** | **1.48586** | **0.00137419** | **1.32349** | **0.000158134** | **1.44806** |
| **ubiquitin associated protein 1** | **UBAP1** | **3.38581e-005** | **1.48523** | **0.00070072** | **1.31634** | **0.000146505** | **1.39697** |
| **transmembrane protein 61** | **TMEM61** | **7.36118e-005** | **1.37642** | **0.000110629** | **1.35625** | **0.000268444** | **1.3151** |
| **Clone IMAGE:120153 mRNA sequence** | **---** | **5.80523e-005** | **1.34388** | **0.00126199** | **1.2232** | **0.0412389** | **1.11443** |
| **RUN domain containing 3A** | **RUNDC3A** | **2.65164e-005** | **1.33858** | **0.0210954** | **1.12066** | **0.000350349** | **1.24064** |
| **Zinc finger, SWIM-type containing 1** | **ZSWIM1** | **1.17266e-006** | **1.33286** | **0.571521** | **1.01771** | **0.261642** | **-1.03625** |
| **hypothetical protein LOC338579** | **LOC338579** | **9.9646e-006** | **1.33096** | **0.097898** | **-1.06993** | **0.0651824** | **1.07955** |
| **keratocan** | **KERA** | **7.07577e-005** | **1.30742** | **0.00180278** | **1.19447** | **0.681719** | **1.01849** |
| **olfactory receptor, family 2, subfamily L, member 2** | **OR2L2** | **5.62892e-005** | **1.30433** | **0.000487804** | **1.22741** | **0.0038992** | **1.1651** |
| **hypothetical protein LOC145845** | **LOC145845** | **7.41069e-005** | **1.26263** | **0.000729902** | **1.19253** | **0.0216361** | **1.10707** |
| **CDNA clone IMAGE:4823340** | **---** | **5.89406e-005** | **1.24399** | **0.0184299** | **1.10063** | **0.328257** | **1.03613** |
| **Transcribed locus, strongly similar to XP_530670.1 PREDICTED: hypothetical prote** | **---** | **9.54122e-006** | **1.20355** | **0.45456** | **1.01886** | **0.379908** | **1.0223** |
| **ubiquitin C** | **UBC** | **7.4032e-005** | **1.17872** | **0.00237149** | **1.11101** | **0.000111829** | **1.16969** |
| **solute carrier family 2 (facilitated glucose transporter), member 3** | **SLC2A3** | **8.11244e-005** | **2.0367** | **0.000169347** | **1.91995** | **0.000184869** | **1.90684** |
| **hydroxysteroid (11-beta) dehydrogenase 1-like** | **HSD11B1L** | **4.24305e-005** | **1.9289** | **0.00128** | **1.53939** | **0.000351019** | **1.66813** |
| **Transcribed locus** | **---** | **4.06478e-005** | **1.7135** | **0.000528417** | **1.48624** | **0.00062794** | **1.47321** |
| **STAM binding protein** | **STAMBP** | **2.14447e-005** | **-1.765** | **0.000537247** | **-1.47501** | **0.000282784** | **-1.52434** |
| **T-cell lymphoma invasion and metastasis 2** | **TIAM2** | **7.07906e-005** | **-2.365** | **0.00105296** | **-1.85037** | **0.000860567** | **-1.88224** |
| **RNA binding protein S1, serine-rich domain** | **RNPS1** | **5.58273e-005** | **-1.2718** | **0.00171086** | **-1.16918** | **0.000271891** | **-1.22068** |
| **metadherin** | **MTDH** | **6.21513e-005** | **-1.3309** | **0.00677726** | **-1.16407** | **0.000161086** | **-1.29145** |
| **G protein-coupled receptor 172A** | **GPR172A** | **3.81109e-005** | **-1.3543** | **0.0039797** | **-1.18105** | **0.00135814** | **-1.2155** |
| **solute carrier family 9 (sodium/hydrogen exchanger), member 3 regulator 1** | **SLC9A3R1** | **4.54861e-005** | **-1.4793** | **0.000252568** | **-1.37744** | **0.00692229** | **-1.2216** |
| **hypothetical protein LOC220906 /// ribosomal protein L7-like 1** | **LOC220906 /// RPL7L1** | **5.58651e-005** | **-1.5436** | **0.000875434** | **-1.36308** | **0.000174147** | **-1.46252** |
| **anaphase promoting complex subunit 4** | **ANAPC4** | **6.74332e-005** | **-1.5678** | **0.0124445** | **-1.2417** | **0.000454904** | **-1.42932** |
| **TruB pseudouridine (psi) synthase homolog 2 (E. coli)** | **TRUB2** | **6.64906e-005** | **-1.5713** | **0.00100771** | **-1.38084** | **0.000130895** | **-1.51841** |
| **ubiquitin specific peptidase 33** | **USP33** | **8.78862e-005** | **-1.6253** | **0.0116682** | **-1.27632** | **0.00176485** | **-1.39256** |
| **LIM domain binding 1** | **LDB1** | **9.82093e-005** | **-1.6298** | **0.0098907** | **-1.29198** | **0.00152625** | **-1.41118** |
| **flotillin 2** | **FLOT2** | **6.42889e-005** | **-1.6441** | **0.00284798** | **-1.35626** | **0.00236451** | **-1.36799** |
| **myeloid differentiation primary response gene (88)** | **MYD88** | **3.33232e-005** | **-1.7249** | **0.000460159** | **-1.49091** | **0.000161814** | **-1.57538** |
| **ralA binding protein 1** | **RALBP1** | **5.69944e-005** | **-1.7454** | **0.000616313** | **-1.51788** | **0.000795298** | **-1.49703** |
| **ATG16 autophagy related 16-like 2 (S. cerevisiae)** | **ATG16L2** | **1.26439e-005** | **-1.8016** | **0.000506679** | **-1.46699** | **0.000242198** | **-1.52267** |
| **TAR (HIV-1) RNA binding protein 1** | **TARBP1** | **7.83101e-005** | **-1.9631** | **0.000692017** | **-1.67561** | **0.000836241** | **-1.65422** |
| **phenylalanyl-tRNA synthetase 2, mitochondrial** | **FARS2** | **0.000100933** | **-1.9968** | **0.00669865** | **-1.47619** | **0.000328487** | **-1.82166** |
| **hypothetical protein LOC643641** | **LOC643641** | **9.30634e-005** | **-2.0308** | **0.0144303** | **-1.41077** | **0.000862821** | **-1.71094** |
| **chromosome 1 open reading frame 181** | **C1orf181** | **4.07181e-005** | **-2.0968** | **0.00135604** | **-1.61618** | **0.00081362** | **-1.67334** |
| **aminolevulinate, delta-, dehydratase** | **ALAD** | **6.85152e-005** | **-2.2134** | **0.000228418** | **-1.992** | **0.00103583** | **-1.76327** |
| **solute carrier family 25 (mitochondrial carrier, Aralar), member 12** | **SLC25A12** | **8.30319e-005** | **-2.2723** | **0.000176099** | **-2.11947** | **0.00018779** | **-2.10721** |
| **interferon regulatory factor 2 binding protein 2** | **IRF2BP2** | **6.02813e-005** | **-2.2909** | **0.00262452** | **-1.66627** | **0.000228489** | **-2.03098** |
| **hypothetical protein LOC145783** | **LOC145783** | **3.22078e-005** | **-2.3521** | **0.00093543** | **-1.76578** | **0.0001108** | **-2.10218** |
| **chromosome 10 open reading frame 97** | **C10orf97** | **4.62291e-005** | **-2.4464** | **0.00140372** | **-1.79594** | **0.000225143** | **-2.10296** |
| **Dicer1, Dcr-1 homolog (Drosophila)** | **DICER1** | **3.36161e-005** | **-2.7764** | **0.000902554** | **-1.98453** | **0.000109169** | **-2.44199** |
| **proteasome (prosome, macropain) subunit, alpha type, 6** | **PSMA6** | **2.50364e-005** | **-1.12058** | **0.00208211** | **-1.06799** | **0.00200121** | **-1.0684** |
| **translocase of outer mitochondrial membrane 20 homolog (yeast)** | **TOMM20** | **8.91901e-005** | **-1.19862** | **0.000503721** | **-1.158** | **0.000262349** | **-1.17264** |
| **lysyl-tRNA synthetase** | **KARS** | **6.4111e-005** | **-1.22107** | **0.00421378** | **-1.12206** | **0.000150844** | **-1.19812** |
| **Deoxythymidylate kinase (thymidylate kinase)** | **DTYMK** | **2.8264e-005** | **-1.24975** | **0.00442084** | **-1.12341** | **0.00150144** | **-1.14663** |
| **NADH dehydrogenase (ubiquinone) 1 beta subcomplex, 8, 19kDa** | **NDUFB8** | **8.34828e-005** | **-1.26516** | **0.000590415** | **-1.20339** | **0.0012524** | **-1.18214** |
| **integrin, beta 2 (complement component 3 receptor 3 and 4 subunit)** | **ITGB2** | **6.77926e-006** | **-1.28331** | **0.000790624** | **-1.15415** | **0.000116355** | **-1.19972** |
| **N-6 adenine-specific DNA methyltransferase 1 (putative)** | **N6AMT1** | **3.01895e-005** | **-1.28394** | **0.00291999** | **-1.15048** | **0.00646448** | **-1.13141** |
| **keratinocyte associated protein 2** | **KRTCAP2** | **8.03475e-005** | **-1.32204** | **0.000928962** | **-1.22876** | **0.000104853** | **-1.31085** |
| **CCR4-NOT transcription complex, subunit 2** | **CNOT2** | **6.07652e-005** | **-1.32961** | **0.00759631** | **-1.15963** | **0.00119714** | **-1.21731** |
| **Parkinson disease (autosomal recessive, early onset) 7** | **PARK7** | **8.46957e-006** | **-1.33309** | **0.0013984** | **-1.16936** | **0.000116928** | **-1.23954** |
| **septin 9** | **SEPT9** | **6.10564e-005** | **-1.34467** | **0.00408812** | **-1.18605** | **0.00100789** | **-1.23296** |
| **phosphonoformate immuno-associated protein 5** | **RP11-298P3.3** | **7.37826e-005** | **-1.36792** | **0.000720462** | **-1.26724** | **0.000351843** | **-1.29663** |
| **cystatin A (stefin A)** | **CSTA** | **7.50595e-005** | **-1.37038** | **0.00400164** | **-1.20504** | **0.000720353** | **-1.26956** |
| **prickle homolog 4 (Drosophila)** | **PRICKLE4** | **2.24163e-005** | **-1.37305** | **0.00143092** | **-1.21042** | **0.00189081** | **-1.20144** |
| **methyltransferase like 9** | **METTL9** | **4.61573e-005** | **-1.38148** | **0.00118564** | **-1.24173** | **0.000312484** | **-1.29411** |
| **mannosidase, alpha, class 2B, member 1** | **MAN2B1** | **9.70851e-005** | **-1.38377** | **0.000867118** | **-1.28054** | **0.000384918** | **-1.31638** |
| **ribosomal protein S19 binding protein 1** | **RPS19BP1** | **4.39848e-005** | **-1.38631** | **0.0136703** | **-1.15856** | **0.000305236** | **-1.29688** |
| **NCK-associated protein 1-like** | **NCKAP1L** | **4.51996e-005** | **-1.41147** | **0.000955789** | **-1.26776** | **0.000145356** | **-1.35135** |
| **nucleoporin 62kDa** | **NUP62** | **4.25576e-005** | **-1.41967** | **0.000737207** | **-1.28159** | **0.000128261** | **-1.36158** |
| **TNF receptor-associated factor 7** | **TRAF7** | **2.50164e-005** | **-1.42773** | **0.000317723** | **-1.30323** | **0.0002048** | **-1.32253** |
| **coiled-coil-helix-coiled-coil-helix domain containing 8** | **CHCHD8** | **7.93701e-005** | **-1.43285** | **0.00253378** | **-1.2584** | **0.000313458** | **-1.35679** |
| **retinoid X receptor, beta** | **RXRB** | **7.75055e-005** | **-1.45169** | **0.00176099** | **-1.28472** | **0.000595898** | **-1.33715** |
| **TP53 activated protein 1** | **TP53AP1** | **4.5128e-005** | **-1.46538** | **0.00150136** | **-1.28026** | **0.000282154** | **-1.36067** |
| **basic transcription factor 3-like 4** | **BTF3L4** | **9.82166e-005** | **-1.46928** | **0.0125449** | **-1.21308** | **0.000229772** | **-1.41576** |
| **vacuolar protein sorting 28 homolog (S. cerevisiae)** | **VPS28** | **7.98087e-005** | **-1.46989** | **0.00234705** | **-1.28291** | **0.000264033** | **-1.39656** |
| **fumarate hydratase** | **FH** | **1.44519e-005** | **-1.48014** | **0.000271278** | **-1.32339** | **0.000115123** | **-1.36433** |
| **chromosome 14 open reading frame 166** | **C14orf166** | **5.8793e-005** | **-1.49116** | **0.0074157** | **-1.23096** | **0.000807844** | **-1.33623** |
| **dolichyl-phosphate (UDP-N-acetylglucosamine) N-acetylglucosaminephosphotransfera** | **DPAGT1** | **7.04081e-005** | **-1.49453** | **0.0710302** | **-1.13906** | **0.00105791** | **-1.33229** |
| **presenilin associated, rhomboid-like** | **PARL** | **6.5898e-005** | **-1.49866** | **0.00149714** | **-1.31412** | **0.000193361** | **-1.42831** |
| **mitochondrial ribosomal protein L52** | **MRPL52** | **7.55371e-005** | **-1.52513** | **0.00318154** | **-1.29578** | **0.00012328** | **-1.48984** |
| **glutathione S-transferase kappa 1** | **GSTK1** | **9.17899e-005** | **-1.52939** | **0.000567052** | **-1.40483** | **0.000151827** | **-1.49247** |
| **TRAF-type zinc finger domain containing 1** | **TRAFD1** | **5.45941e-005** | **-1.53127** | **0.000435067** | **-1.39419** | **0.000132769** | **-1.46876** |
| **tRNA isopentenyltransferase 1** | **TRIT1** | **8.97214e-005** | **-1.53946** | **0.00571888** | **-1.27902** | **0.000150247** | **-1.50087** |
| **BRCA1/BRCA2-containing complex, subunit 3** | **BRCC3** | **5.17693e-005** | **-1.54441** | **0.000543439** | **-1.38741** | **0.000280242** | **-1.42772** |
| **solute carrier family 9 (sodium/hydrogen exchanger), member 8** | **SLC9A8** | **3.89184e-005** | **-1.55179** | **0.0176212** | **-1.20421** | **0.0027132** | **-1.29218** |
| **mediator complex subunit 30** | **MED30** | **5.67178e-005** | **-1.55379** | **0.00110461** | **-1.35687** | **0.00011041** | **-1.50394** |
| **phosphoinositide-3-kinase, regulatory subunit 1 (p85 alpha)** | **PIK3R1** | **7.88263e-005** | **-1.55797** | **0.00266516** | **-1.32443** | **0.00186165** | **-1.34478** |
| **sorcin** | **SRI** | **3.15777e-005** | **-1.56191** | **0.000319956** | **-1.40493** | **0.00113163** | **-1.33401** |
| **nuclear factor (erythroid-derived 2)-like 1** | **NFE2L1** | **3.61636e-005** | **-1.57035** | **0.000533358** | **-1.3873** | **0.000145214** | **-1.46905** |
| **hypothetical protein LOC145758** | **LOC145758** | **6.51632e-005** | **-1.57762** | **0.0215938** | **-1.21658** | **0.00277439** | **-1.32442** |
| **chromosome 7 open reading frame 28A** | **C7orf28A** | **5.26239e-005** | **-1.58555** | **0.00240498** | **-1.32742** | **0.000110525** | **-1.52679** |
| **RAB GTPase activating protein 1** | **RABGAP1** | **9.68849e-005** | **-1.59606** | **0.00395129** | **-1.3306** | **0.000516908** | **-1.46369** |
| **zinc finger, MIZ-type containing 2** | **ZMIZ2** | **9.08618e-005** | **-1.59803** | **0.00033416** | **-1.49293** | **0.000157767** | **-1.55157** |
| **family with sequence similarity 39, member D pseudogene /// family with sequence** | **CXYorf1 /// FAM39B /// FAM39DP /// FAM39E /// LOC376475 /// LOC653635** | **5.63145e-005** | **-1.59955** | **0.00110406** | **-1.38408** | **0.000731284** | **-1.41005** |
| **Discs, large homolog 1 (Drosophila)** | **DLG1** | **2.3285e-005** | **-1.60667** | **0.0119763** | **-1.22643** | **0.00027961** | **-1.4269** |
| **nucleoporin 93kDa** | **NUP93** | **6.71235e-005** | **-1.60981** | **0.00070184** | **-1.42944** | **0.000213948** | **-1.51481** |
| **chromosome Y open reading frame 15B** | **CYorf15B** | **4.01814e-005** | **-1.61004** | **0.000461258** | **-1.42783** | **0.000366911** | **-1.4429** |
| **protein kinase, cAMP-dependent, catalytic, alpha** | **PRKACA** | **3.17184e-005** | **-1.62413** | **0.000512627** | **-1.4169** | **0.00027136** | **-1.45878** |
| **transmembrane protein 179B** | **TMEM179B** | **3.28106e-005** | **-1.63219** | **0.00291475** | **-1.31969** | **0.000194179** | **-1.49016** |
| **signal transducer and activator of transcription 5A** | **STAT5A** | **3.97644e-005** | **-1.63358** | **0.00148146** | **-1.36812** | **0.000634802** | **-1.4215** |
| **coenzyme Q6 homolog, monooxygenase (S. cerevisiae)** | **COQ6** | **5.11785e-005** | **-1.64862** | **0.00238751** | **-1.35875** | **0.00015362** | **-1.55278** |
| **mitochondrial ribosomal protein 63** | **MRP63** | **8.37002e-005** | **-1.65101** | **0.00353342** | **-1.35861** | **0.000356301** | **-1.52373** |
| **arginine-glutamic acid dipeptide (RE) repeats** | **RERE** | **6.97455e-005** | **-1.66012** | **0.00026865** | **-1.54011** | **0.000169015** | **-1.5792** |
| **PHD finger protein 3** | **PHF3** | **7.25836e-005** | **-1.66661** | **0.00944338** | **-1.29801** | **0.000818875** | **-1.46068** |
| **Pentatricopeptide repeat domain 3** | **PTCD3** | **2.11632e-005** | **-1.67096** | **0.00158939** | **-1.35362** | **0.000774825** | **-1.39687** |
| **Mitochondrial ribosomal protein L41** | **MRPL41** | **4.5838e-005** | **-1.67166** | **0.00148749** | **-1.39548** | **0.000301997** | **-1.50888** |
| **Rho GTPase activating protein 1** | **ARHGAP1** | **9.20395e-005** | **-1.68004** | **0.00504583** | **-1.35388** | **0.000215024** | **-1.59845** |
| **LIM domain only 4** | **LMO4** | **2.68772e-005** | **-1.68144** | **0.000402446** | **-1.45957** | **0.000135323** | **-1.54056** |
| **NADH dehydrogenase (ubiquinone) Fe-S protein 2, 49kDa (NADH-coenzyme Q reductase** | **NDUFS2** | **3.55512e-005** | **-1.68829** | **0.000983593** | **-1.41851** | **0.000288838** | **-1.5065** |
| **LIM domain binding 1** | **LDB1** | **8.15541e-005** | **-1.69601** | **0.00293647** | **-1.39265** | **0.00154258** | **-1.43908** |
| **general transcription factor II, i /// general transcription factor II, i, pseud** | **GTF2I /// GTF2IP1 /// LOC100093631 /// LOC732437** | **3.44645e-005** | **-1.70035** | **0.00181221** | **-1.38293** | **0.000150048** | **-1.56554** |
| **M-phase phosphoprotein 10 (U3 small nucleolar ribonucleoprotein)** | **MPHOSPH10** | **6.44522e-005** | **-1.71463** | **0.0178188** | **-1.27235** | **0.00119219** | **-1.45485** |
| **family with sequence similarity 82, member B /// similar to Protein FAM82B** | **FAM82B /// LOC642197** | **7.05132e-005** | **-1.71591** | **0.00530228** | **-1.35432** | **0.000355268** | **-1.56024** |
| **canopy 2 homolog (zebrafish)** | **CNPY2** | **8.41079e-005** | **-1.73241** | **0.00158023** | **-1.46037** | **0.00116871** | **-1.48444** |
| **exosome component 7** | **EXOSC7** | **2.83637e-005** | **-1.73316** | **0.0660427** | **-1.17856** | **0.00105566** | **-1.42561** |
| **CD99 molecule-like 2** | **CD99L2** | **8.81906e-005** | **-1.73686** | **0.0991635** | **-1.17996** | **0.00062902** | **-1.54301** |
| **phosphatidylinositol glycan anchor biosynthesis, class S** | **PIGS** | **4.03232e-006** | **-1.74703** | **0.00241198** | **-1.29646** | **0.000123025** | **-1.46699** |
| **isoleucyl-tRNA synthetase 2, mitochondrial** | **IARS2** | **3.03365e-005** | **-1.75835** | **0.00466921** | **-1.34219** | **0.000161934** | **-1.59296** |
| **family with sequence similarity 78, member A** | **FAM78A** | **9.258e-006** | **-1.76214** | **0.00158716** | **-1.3576** | **0.000116002** | **-1.53351** |
| **pyruvate dehydrogenase (lipoamide) beta** | **PDHB** | **8.56049e-005** | **-1.77225** | **0.00209902** | **-1.46128** | **0.000120183** | **-1.73328** |
| **eukaryotic translation initiation factor 2B, subunit 1 alpha, 26kDa** | **EIF2B1** | **7.91821e-005** | **-1.77346** | **0.00479605** | **-1.39308** | **0.00018927** | **-1.6769** |
| **anaphase promoting complex subunit 5** | **ANAPC5** | **6.50978e-005** | **-1.77807** | **0.00062815** | **-1.54759** | **0.000353981** | **-1.60007** |
| **nucleoporin 214kDa** | **NUP214** | **1.07315e-005** | **-1.77928** | **0.00204607** | **-1.35598** | **0.00011174** | **-1.55848** |
| **Transcribed locus** | **---** | **2.29861e-005** | **-1.78866** | **0.000180416** | **-1.5829** | **0.000473191** | **-1.50293** |
| **PRP31 pre-mRNA processing factor 31 homolog (S. cerevisiae)** | **PRPF31** | **6.34882e-005** | **-1.79155** | **0.021319** | **-1.28485** | **0.00463894** | **-1.39196** |
| **chromosome 3 open reading frame 21** | **C3orf21** | **4.63809e-005** | **-1.80032** | **0.023787** | **-1.26912** | **0.000142274** | **-1.67617** |
| **Interleukin 12 receptor, beta 1** | **IL12RB1** | **5.44261e-005** | **-1.80179** | **0.000572767** | **-1.55745** | **0.00147462** | **-1.47671** |
| **NCK interacting protein with SH3 domain** | **NCKIPSD** | **6.10714e-005** | **-1.80417** | **0.00122688** | **-1.50105** | **0.00184277** | **-1.46734** |
| **NudC domain containing 2** | **NUDCD2** | **9.98856e-005** | **-1.81199** | **0.00172631** | **-1.51084** | **0.00196389** | **-1.49938** |
| **prolyl endopeptidase-like** | **PREPL** | **2.74358e-005** | **-1.81556** | **0.00235191** | **-1.4076** | **0.000198364** | **-1.60773** |
| **C2 calcium-dependent domain containing 3** | **C2CD3** | **7.82291e-005** | **-1.81788** | **0.00010415** | **-1.78271** | **0.00320613** | **-1.44478** |
| **anaphase promoting complex subunit 7** | **ANAPC7** | **7.49738e-005** | **-1.82536** | **0.00197195** | **-1.4865** | **0.000756446** | **-1.57275** |
| **CTD (carboxy-terminal domain, RNA polymerase II, polypeptide A) small phosphatas** | **CTDSP2** | **6.42856e-005** | **-1.83213** | **0.00106738** | **-1.53314** | **0.000305818** | **-1.65354** |
| **sorting and assembly machinery component 50 homolog (S. cerevisiae)** | **SAMM50** | **2.38012e-005** | **-1.83487** | **0.00113066** | **-1.46271** | **0.000364683** | **-1.55471** |
| **CDNA clone IMAGE:4814828** | **---** | **8.64755e-005** | **-1.83507** | **0.00030262** | **-1.68613** | **0.00125883** | **-1.5424** |
| **DnaJ (Hsp40) homolog, subfamily C, member 11** | **DNAJC11** | **9.7053e-006** | **-1.83816** | **0.00127124** | **-1.40584** | **0.000286367** | **-1.51294** |
| **histidine triad nucleotide binding protein 2** | **HINT2** | **8.01532e-005** | **-1.83822** | **0.000681145** | **-1.59697** | **0.00056499** | **-1.6156** |
| **selectin P ligand** | **SELPLG** | **8.76049e-005** | **-1.84803** | **0.00110146** | **-1.56393** | **0.000228847** | **-1.72951** |
| **WW domain containing E3 ubiquitin protein ligase 1** | **WWP1** | **1.51973e-005** | **-1.85047** | **0.000256441** | **-1.56085** | **0.000128705** | **-1.62227** |
| **myotubularin related protein 4** | **MTMR4** | **5.03585e-005** | **-1.85177** | **0.00620358** | **-1.38339** | **0.000727304** | **-1.56109** |
| **zinc finger protein 161 homolog (mouse)** | **ZFP161** | **9.23475e-005** | **-1.87381** | **0.00388979** | **-1.46588** | **0.000122746** | **-1.83565** |
| **related RAS viral (r-ras) oncogene homolog** | **RRAS** | **5.86182e-005** | **-1.87434** | **0.00266596** | **-1.46938** | **0.000129334** | **-1.77389** |
| **integrin alpha FG-GAP repeat containing 2** | **ITFG2** | **6.08376e-005** | **-1.88473** | **0.00486719** | **-1.42613** | **0.000125397** | **-1.79104** |
| **elongation protein 3 homolog (S. cerevisiae)** | **ELP3** | **7.62459e-005** | **-1.88818** | **0.00527901** | **-1.43407** | **0.000650818** | **-1.63042** |
| **MIT, microtubule interacting and transport, domain containing 1** | **MITD1** | **4.24574e-005** | **-1.89084** | **0.00078446** | **-1.56391** | **0.000568027** | **-1.59458** |
| **ankyrin repeat and FYVE domain containing 1** | **ANKFY1** | **4.3807e-005** | **-1.89623** | **0.00643923** | **-1.39057** | **0.00129635** | **-1.52338** |
| **Hypothetical protein LOC729082** | **LOC729082** | **3.96006e-005** | **-1.90874** | **0.00102444** | **-1.54396** | **0.000335559** | **-1.6528** |
| **transcriptional regulating factor 1** | **TRERF1** | **4.41307e-005** | **-1.91508** | **0.000541198** | **-1.61773** | **0.00224286** | **-1.48509** |
| **zinc finger protein 627** | **ZNF627** | **2.47159e-005** | **-1.91517** | **0.000902432** | **-1.52437** | **0.000316809** | **-1.62066** |
| **KIAA0329** | **KIAA0329** | **3.60346e-005** | **-1.92297** | **0.0006016** | **-1.5948** | **0.000441827** | **-1.62535** |
| **vacuolar protein sorting 26 homolog B (S. pombe)** | **VPS26B** | **1.02247e-005** | **-1.93584** | **0.000343637** | **-1.55522** | **0.000171051** | **-1.61783** |
| **high-mobility group 20A** | **HMG20A** | **8.45378e-005** | **-1.94143** | **0.00251807** | **-1.53335** | **0.000409806** | **-1.73002** |
| **MFNG O-fucosylpeptide 3-beta-N-acetylglucosaminyltransferase** | **MFNG** | **4.41145e-005** | **-1.94862** | **0.00259564** | **-1.48782** | **0.00011307** | **-1.81992** |
| **unc-51-like kinase 3 (C. elegans)** | **ULK3** | **4.5232e-005** | **-1.95474** | **0.000455695** | **-1.66316** | **0.000539319** | **-1.64511** |
| **tudor domain containing 3** | **TDRD3** | **4.59224e-005** | **-1.96245** | **0.00501225** | **-1.43911** | **0.00539026** | **-1.43294** |
| **zinc finger protein 248** | **ZNF248** | **8.00194e-005** | **-1.96313** | **0.00241467** | **-1.5443** | **0.00039838** | **-1.7433** |
| **alanyl-tRNA synthetase 2, mitochondrial (putative)** | **AARS2** | **8.19082e-005** | **-1.96932** | **0.000452152** | **-1.73486** | **0.000264236** | **-1.80284** |
| **chromobox homolog 1 (HP1 beta homolog Drosophila )** | **CBX1** | **7.93507e-005** | **-1.97853** | **0.0140271** | **-1.38706** | **0.00095824** | **-1.64982** |
| **nudix (nucleoside diphosphate linked moiety X)-type motif 13** | **NUDT13** | **3.88923e-005** | **-1.97971** | **0.00021185** | **-1.75193** | **0.000254684** | **-1.73018** |
| **Nedd4 binding protein 1** | **N4BP1** | **4.0338e-005** | **-1.98807** | **0.000261667** | **-1.73677** | **0.000575226** | **-1.64813** |
| **syntaxin 10** | **STX10** | **7.43836e-005** | **-1.99008** | **0.00314961** | **-1.52557** | **0.00130375** | **-1.61673** |
| **DEAD (Asp-Glu-Ala-Asp) box polypeptide 58** | **DDX58** | **3.9916e-005** | **-1.99809** | **0.000103534** | **-1.86031** | **0.000160276** | **-1.80304** |
| **bromodomain containing 8** | **BRD8** | **8.85084e-005** | **-1.99994** | **0.00329562** | **-1.53883** | **0.00130589** | **-1.63814** |
| **RAN binding protein 10** | **RANBP10** | **2.96201e-005** | **-2.01763** | **0.000235132** | **-1.73679** | **0.000147206** | **-1.79347** |
| **membrane bound O-acyltransferase domain containing 1** | **MBOAT1** | **3.28203e-005** | **-2.02279** | **0.00052215** | **-1.66016** | **0.0003222** | **-1.71424** |
| **deoxythymidylate kinase (thymidylate kinase) /// similar to deoxythymidylate kin** | **DTYMK /// LOC727761** | **1.1839e-005** | **-2.03157** | **0.00377007** | **-1.41224** | **0.000723219** | **-1.5486** |
| **Clone CDABP0105 mRNA sequence** | **---** | **3.63749e-005** | **-2.05393** | **0.00020416** | **-1.80251** | **0.000878629** | **-1.63088** |
| **leucyl-tRNA synthetase** | **LARS** | **6.7618e-005** | **-2.05527** | **0.0046746** | **-1.50841** | **0.000931217** | **-1.68366** |
| **chromosome 14 open reading frame 159** | **C14orf159** | **9.48153e-005** | **-2.08454** | **0.000427287** | **-1.8437** | **0.000165422** | **-1.98937** |
| **regulatory factor X-associated protein** | **RFXAP** | **1.39312e-005** | **-2.09397** | **0.00104864** | **-1.55491** | **0.000115837** | **-1.79102** |
| **G protein-coupled receptor kinase interactor 2** | **GIT2** | **8.58603e-005** | **-2.09652** | **0.0039349** | **-1.56243** | **0.00087652** | **-1.74157** |
| **chromosome 1 open reading frame 59** | **C1orf59** | **3.41475e-005** | **-2.09739** | **0.000237286** | **-1.80602** | **0.000173988** | **-1.84746** |
| **WD repeat domain 77** | **WDR77** | **1.71225e-005** | **-2.10856** | **0.000128965** | **-1.81111** | **0.000177132** | **-1.77158** |
| **speckle-type POZ protein** | **SPOP** | **3.88193e-005** | **-2.11113** | **0.00382187** | **-1.512** | **0.000745419** | **-1.68684** |
| **Transcribed locus** | **---** | **6.43121e-005** | **-2.11123** | **0.00197593** | **-1.6218** | **0.000523411** | **-1.78574** |
| **insulin induced gene 2** | **INSIG2** | **2.05755e-005** | **-2.11491** | **0.00218566** | **-1.52282** | **0.000316379** | **-1.72561** |
| **R3H domain and coiled-coil containing 1** | **R3HCC1** | **3.37778e-005** | **-2.12056** | **0.00855529** | **-1.43087** | **0.00500382** | **-1.48032** |
| **membrane interacting protein of RGS16** | **MIR16** | **6.54127e-005** | **-2.12399** | **0.00177972** | **-1.64186** | **0.000745968** | **-1.74906** |
| **chloride channel, nucleotide-sensitive, 1A** | **CLNS1A** | **2.58672e-005** | **-2.13002** | **0.00281784** | **-1.52069** | **0.000217635** | **-1.80653** |
| **mitochondrial translation optimization 1 homolog (S. cerevisiae)** | **MTO1** | **1.50013e-005** | **-2.13747** | **0.000588808** | **-1.63879** | **0.000205347** | **-1.75748** |
| **chromosome 17 open reading frame 62** | **C17orf62** | **8.91972e-005** | **-2.14533** | **0.00153208** | **-1.70223** | **0.000592041** | **-1.83169** |
| **ribonuclease L (2',5'-oligoisoadenylate synthetase-dependent)** | **RNASEL** | **5.28081e-005** | **-2.15083** | **0.00157393** | **-1.64984** | **0.00217161** | **-1.61275** |
| **FLJ35348** | **FLJ35348** | **7.69283e-005** | **-2.15122** | **0.000765427** | **-1.7811** | **0.000531635** | **-1.83219** |
| **chromosome 2 open reading frame 7** | **C2orf7** | **3.37729e-005** | **-2.16805** | **0.00195131** | **-1.59445** | **0.000793614** | **-1.69673** |
| **interleukin 15 receptor, alpha** | **IL15RA** | **2.01434e-005** | **-2.18693** | **0.000597875** | **-1.6893** | **0.000135149** | **-1.87872** |
| **ORAI calcium release-activated calcium modulator 1** | **ORAI1** | **6.74485e-005** | **-2.20297** | **0.00104056** | **-1.75494** | **0.000188549** | **-2.01362** |
| **ataxia telangiectasia and Rad3 related /// similar to ataxia telangiectasia and** | **ATR /// LOC648152** | **7.21564e-005** | **-2.20909** | **0.000895603** | **-1.78694** | **0.000148447** | **-2.07188** |
| **bromodomain and WD repeat domain containing 1** | **BRWD1** | **4.70123e-005** | **-2.23765** | **0.0010208** | **-1.73666** | **0.000981456** | **-1.74182** |
| **metallophosphoesterase 1** | **MPPE1** | **7.88195e-006** | **-2.24198** | **0.000251679** | **-1.72594** | **0.000107289** | **-1.83039** |
| **methylcrotonoyl-Coenzyme A carboxylase 1 (alpha)** | **MCCC1** | **3.50251e-005** | **-2.24356** | **0.000356036** | **-1.84958** | **0.000185316** | **-1.94731** |
| **hypothetical protein LOC100049716** | **LOC100049716** | **7.6254e-005** | **-2.25445** | **0.000155153** | **-2.11265** | **0.000158049** | **-2.10917** |
| **Transcribed locus** | **---** | **6.59657e-005** | **-2.27488** | **0.000500828** | **-1.90269** | **0.00211729** | **-1.69561** |
| **acyl-CoA synthetase family member 2** | **ACSF2** | **4.60848e-005** | **-2.30557** | **0.000494763** | **-1.87511** | **0.000240326** | **-1.99037** |
| **hypothetical protein LOC134145** | **LOC134145** | **4.09363e-005** | **-2.30903** | **0.00307136** | **-1.61899** | **0.000182212** | **-2.02128** |
| **Alstrom syndrome 1** | **ALMS1** | **6.86641e-005** | **-2.31302** | **0.000212185** | **-2.08355** | **0.000527684** | **-1.92489** |
| **Senataxin** | **SETX** | **9.49431e-005** | **-2.31326** | **0.00202203** | **-1.76069** | **0.000928534** | **-1.87943** |
| **hypothetical protein LOC56757** | **LOC56757** | **1.8283e-005** | **-2.31838** | **0.00069746** | **-1.72693** | **0.000121348** | **-1.97213** |
| **protein tyrosine phosphatase, receptor type, O** | **PTPRO** | **9.29869e-005** | **-2.32568** | **0.00122347** | **-1.84038** | **0.000136629** | **-2.24055** |
| **Transcribed locus** | **---** | **3.49695e-005** | **-2.33302** | **0.000462747** | **-1.86547** | **0.000127667** | **-2.07584** |
| **mitochondrial ribosomal protein S14** | **MRPS14** | **8.24489e-005** | **-2.33995** | **0.000132693** | **-2.23467** | **0.000467884** | **-1.99097** |
| **COMM domain containing 3** | **COMMD3** | **9.51628e-005** | **-2.34958** | **0.00165487** | **-1.80989** | **0.00106673** | **-1.87917** |
| **retinoblastoma-like 1 (p107)** | **RBL1** | **8.39867e-005** | **-2.35267** | **0.000180978** | **-2.1846** | **0.000231228** | **-2.13514** |
| **DEAH (Asp-Glu-Ala-His) box polypeptide 40** | **DHX40** | **2.0531e-005** | **-2.39283** | **0.00166932** | **-1.66408** | **0.00226155** | **-1.62794** |
| **WD repeat domain 7** | **WDR7** | **1.7911e-005** | **-2.39907** | **0.000306783** | **-1.87813** | **0.000147198** | **-1.99215** |
| **KIAA1432** | **KIAA1432** | **4.9864e-005** | **-2.41492** | **0.000609986** | **-1.91847** | **0.000310594** | **-2.03423** |
| **PYD and CARD domain containing** | **PYCARD** | **8.52784e-005** | **-2.42113** | **0.00247587** | **-1.77135** | **0.000426632** | **-2.07006** |
| **tumor suppressor candidate 4** | **TUSC4** | **4.30326e-005** | **-2.47271** | **0.000428821** | **-1.99094** | **0.000121745** | **-2.23291** |
| **CDNA FLJ34214 fis, clone FCBBF3021807** | **---** | **6.79613e-005** | **-2.48074** | **0.000165672** | **-2.26713** | **0.000434534** | **-2.06804** |
| **TBC1 domain family, member 2B** | **TBC1D2B** | **7.91681e-005** | **-2.50813** | **0.000396849** | **-2.13204** | **0.000195389** | **-2.28526** |
| **xeroderma pigmentosum, complementation group A** | **XPA** | **4.35188e-005** | **-2.51847** | **0.00270713** | **-1.72598** | **0.00019278** | **-2.17448** |
| **transmembrane protein 97** | **TMEM97** | **6.88748e-005** | **-2.52282** | **0.00386522** | **-1.72523** | **0.000113337** | **-2.39509** |
| **chromosome 17 open reading frame 90** | **C17orf90** | **9.26627e-006** | **-2.53189** | **0.0001862** | **-1.94008** | **0.00015059** | **-1.97353** |
| **epithelial cell transforming sequence 2 oncogene** | **ECT2** | **6.22083e-005** | **-2.61786** | **0.000954735** | **-1.98777** | **0.00019954** | **-2.31458** |
| **MKL/myocardin-like 2** | **MKL2** | **7.77412e-005** | **-2.72871** | **0.00283436** | **-1.87623** | **0.000371179** | **-2.29733** |
| **synaptojanin 2 binding protein** | **SYNJ2BP** | **4.58004e-005** | **-2.79922** | **0.00396611** | **-1.78037** | **0.000221477** | **-2.35398** |
| **ataxia telangiectasia and Rad3 related** | **ATR** | **4.09811e-005** | **-2.82471** | **0.00149132** | **-1.94412** | **0.000365436** | **-2.22912** |
| **arrestin, beta 1** | **ARRB1** | **9.32089e-005** | **-2.85588** | **0.00212492** | **-2.01622** | **0.000418896** | **-2.39756** |
| **Arachidonate 5-lipoxygenase** | **ALOX5** | **9.84627e-005** | **-2.90155** | **0.001082** | **-2.19955** | **0.000102777** | **-2.88616** |
| **hypothetical protein LOC84792** | **MGC12966** | **6.77153e-005** | **-2.92163** | **0.000715994** | **-2.23274** | **0.000109761** | **-2.75602** |
| **PTPRF interacting protein, binding protein 2 (liprin beta 2)** | **PPFIBP2** | **9.58289e-005** | **-2.92187** | **0.000500175** | **-2.4019** | **0.00025516** | **-2.59532** |
| **adrenergic, beta, receptor kinase 2** | **ADRBK2** | **4.40721e-005** | **-2.99322** | **0.000475087** | **-2.2825** | **0.000103415** | **-2.70282** |
| **CTD (carboxy-terminal domain, RNA polymerase II, polypeptide A) small phosphatas** | **CTDSP2** | **7.75994e-005** | **-3.04564** | **0.00723507** | **-1.82293** | **0.000276793** | **-2.60396** |
| **serine/threonine kinase 36, fused homolog (Drosophila)** | **STK36** | **4.69358e-005** | **-3.36266** | **0.000887738** | **-2.33224** | **0.000761019** | **-2.37377** |
| **NLR family, CARD domain containing 4** | **NLRC4** | **1.22771e-005** | **-3.91985** | **0.000184016** | **-2.73376** | **0.000135574** | **-2.83802** |
| **5.5 kb mRNA upregulated in retinoic acid treated HL-60 neutrophilic cells** | **---** | **1.50913e-005** | **-3.95695** | **0.00103375** | **-2.29392** | **0.000357198** | **-2.59646** |
| **Transcriptional regulating factor 1** | **TRERF1** | **4.08183e-005** | **-4.20779** | **0.00106701** | **-2.62056** | **0.000153032** | **-3.43345** |
| **HSPB (heat shock 27kDa) associated protein 1** | **HSPBAP1** | **3.94348e-005** | **-4.30775** | **0.000667305** | **-2.82583** | **0.000198278** | **-3.35545** |
| **Ca2+-dependent secretion activator** | **CADPS** | **0.00536448** | **1.23068** | **8.91246e-005** | **1.43383** | **0.000255067** | **1.37416** |
| **prion protein 2 (dublet)** | **PRND** | **0.000932999** | **1.23055** | **4.13478e-005** | **1.35445** | **0.0112281** | **1.15125** |
| **thyrotropin-releasing hormone degrading enzyme** | **TRHDE** | **0.00128138** | **1.19274** | **7.76288e-005** | **1.28485** | **0.176453** | **1.06118** |
| **tudor domain containing 5** | **TDRD5** | **0.00788581** | **1.11279** | **6.85785e-005** | **1.22638** | **0.00232595** | **1.13869** |
| **Clone 23738 mRNA sequence** | **---** | **0.00164368** | **1.2438** | **9.33032e-005** | **1.36966** | **0.00215206** | **1.23347** |
| **chromosome 5 open reading frame 41** | **C5orf41** | **0.000567614** | **-1.4161** | **2.53486e-005** | **-1.65353** | **0.000235744** | **-1.47501** |
| **hypothetical protein LOC285147** | **LOC285147** | **0.000170726** | **-1.19016** | **1.10538e-005** | **-1.26743** | **0.000198609** | **-1.18642** |
| **chromosome 1 open reading frame 66** | **C1orf66** | **0.0192119** | **-1.25828** | **9.22227e-005** | **-1.65052** | **0.000283277** | **-1.54965** |
| **COX15 homolog, cytochrome c oxidase assembly protein (yeast)** | **COX15** | **0.00298399** | **-1.36943** | **9.7388e-005** | **-1.63629** | **0.000299983** | **-1.53746** |
| **CDNA clone IMAGE:4304686** | **---** | **0.000467425** | **-1.40035** | **7.79421e-005** | **-1.51946** | **0.000129583** | **-1.48327** |
| **CDNA: FLJ23242 fis, clone COL01514** | **---** | **0.000475154** | **-1.44274** | **7.86408e-006** | **-1.79931** | **0.000150538** | **-1.52573** |
| **Transcribed locus, strongly similar to XP_529518.1 PREDICTED: hypothetical prote** | **---** | **0.000155827** | **-1.51549** | **2.32991e-005** | **-1.67603** | **0.000271274** | **-1.47501** |
| **dynein, axonemal, heavy chain 1** | **DNAH1** | **0.000150033** | **-1.53855** | **5.81119e-005** | **-1.61789** | **0.000104532** | **-1.56773** |
| **Transcribed locus** | **---** | **0.000178308** | **-1.54617** | **2.44805e-005** | **-1.72836** | **0.000473317** | **-1.47113** |
| **Virus-induced signaling adapter** | **VISA** | **0.000291381** | **-1.72957** | **4.07134e-005** | **-1.99453** | **0.000187217** | **-1.7831** |
| **CDNA clone IMAGE:4157286** | **---** | **0.000122704** | **-1.79251** | **1.07418e-005** | **-2.15161** | **0.000889782** | **-1.57633** |
| **tigger transposable element derived 2** | **TIGD2** | **0.00037233** | **-1.96398** | **8.65672e-005** | **-2.23641** | **0.000860668** | **-1.83171** |
| **metallothionein 1X** | **MT1X** | **0.000149648** | **3.47208** | **0.000319445** | **3.11062** | **3.00072e-005** | **4.46602** |
| **metallothionein 1X** | **MT1X** | **0.000232234** | **2.66164** | **0.000598304** | **2.3838** | **5.30768e-005** | **3.20376** |
| **metallothionein 1F** | **MT1F** | **0.000211204** | **2.64875** | **0.000573519** | **2.36119** | **4.83875e-005** | **3.18118** |
| **metallothionein 1 pseudogene 2** | **MT1P2** | **0.000128921** | **2.52661** | **0.000448618** | **2.21519** | **2.96496e-005** | **2.99287** |
| **metallothionein 1F** | **MT1F** | **0.000180762** | **2.41809** | **0.000417986** | **2.21667** | **3.93967e-005** | **2.8676** |
| **prostaglandin I2 (prostacyclin) receptor (IP)** | **PTGIR** | **0.000124167** | **2.22931** | **0.00118347** | **1.82598** | **8.34724e-005** | **2.31582** |
| **spectrin, beta, non-erythrocytic 4** | **SPTBN4** | **0.000178514** | **2.05116** | **0.000325847** | **1.94895** | **6.31667e-005** | **2.25102** |
| **FK506 binding protein 4, 59kDa** | **FKBP4** | **0.000346261** | **1.54488** | **0.000794986** | **1.47788** | **3.06756e-005** | **1.78243** |
| **thyroid hormone receptor, alpha (erythroblastic leukemia viral (v-erb-a) oncogen** | **THRA** | **0.0011747** | **1.52448** | **0.000676518** | **1.57497** | **2.11621e-005** | **1.98437** |
| **melanocortin 1 receptor (alpha melanocyte stimulating hormone receptor) /// tubu** | **MC1R /// TUBB3** | **0.000240908** | **1.41137** | **0.000228044** | **1.41465** | **8.95921e-005** | **1.47355** |
| **protocadherin gamma subfamily A, 12 /// protocadherin gamma subfamily A, 11 ///** | **PCDHGA10 /// PCDHGA11 /// PCDHGA12 /// PCDHGA3 /// PCDHGA5 /// PCDHGA6** | **0.000194323** | **1.38285** | **0.00381714** | **1.24142** | **4.28124e-005** | **1.47183** |
| **actin filament associated protein 1-like 1** | **AFAP1L1** | **0.000634866** | **1.34747** | **0.000243109** | **1.40047** | **2.12785e-005** | **1.56193** |
| **v-maf musculoaponeurotic fibrosarcoma oncogene homolog (avian)** | **MAF** | **0.00433561** | **1.26892** | **0.0171996** | **1.20509** | **4.90906e-005** | **1.53371** |
| **hypothetical protein BC009233** | **LOC92659** | **0.00259488** | **1.17243** | **0.000192399** | **1.25236** | **3.90515e-006** | **1.41445** |
| **Transcribed locus** | **---** | **0.039754** | **1.14801** | **0.00940728** | **1.20428** | **7.89605e-005** | **1.4337** |
| **protocadherin alpha 9 /// protocadherin alpha subfamily C, 2 /// protocadherin a** | **PCDHA1 /// PCDHA10 /// PCDHA11 /// PCDHA12 /// PCDHA13 /// PCDHA2 /// PCDHA3 ///** | **0.000625237** | **1.10426** | **0.000697053** | **1.10271** | **1.23405e-005** | **1.16998** |
| **opioid receptor, sigma 1** | **OPRS1** | **0.000709483** | **-1.5545** | **0.00558777** | **-1.38544** | **7.95075e-005** | **-1.78136** |
| **c-src tyrosine kinase** | **CSK** | **0.000169615** | **-1.5859** | **0.000542682** | **-1.49028** | **7.30841e-005** | **-1.66374** |
| **TRAF2 and NCK interacting kinase** | **TNIK** | **0.000150726** | **-2.0837** | **0.00045535** | **-1.8975** | **3.29761e-005** | **-2.39693** |
| **coiled-coil domain containing 128** | **CCDC128** | **0.000106369** | **-2.2981** | **0.000520423** | **-1.98343** | **9.6587e-005** | **-2.31987** |
| **mutL homolog 3 (E. coli)** | **MLH3** | **0.0110329** | **-1.20522** | **0.00166047** | **-1.28783** | **3.7421e-005** | **-1.49972** |
| **synaptogyrin 2** | **SYNGR2** | **0.000281456** | **-1.21813** | **0.0032043** | **-1.15205** | **6.04858e-005** | **-1.26714** |
| **HECT domain and ankyrin repeat containing, E3 ubiquitin protein ligase 1** | **HACE1** | **0.0124955** | **-1.24114** | **0.00186319** | **-1.34268** | **1.8508e-005** | **-1.67981** |
| **mitogen-activated protein kinase kinase 5** | **MAP2K5** | **0.00868219** | **-1.24569** | **0.000126235** | **-1.48794** | **9.53199e-006** | **-1.69924** |
| **UCHL5 interacting protein** | **UCHL5IP** | **0.000173941** | **-1.31729** | **0.000355917** | **-1.28706** | **1.62697e-005** | **-1.43432** |
| **huntingtin interacting protein 1 related /// similar to huntingtin interacting p** | **HIP1R /// LOC728014** | **0.00178238** | **-1.34471** | **0.0164769** | **-1.22699** | **2.03309e-005** | **-1.66987** |
| **UPF2 regulator of nonsense transcripts homolog (yeast)** | **UPF2** | **0.000101113** | **-1.36785** | **0.0431245** | **-1.12869** | **7.76956e-005** | **-1.38121** |
| **TBC1 domain family, member 16** | **TBC1D16** | **0.000198324** | **-1.37003** | **0.00124934** | **-1.28172** | **2.49068e-005** | **-1.49132** |
| **tumor necrosis factor (ligand) superfamily, member 13b** | **TNFSF13B** | **0.0003927** | **-1.37576** | **0.000332401** | **-1.3851** | **7.52354e-005** | **-1.47583** |
| **coatomer protein complex, subunit gamma** | **COPG** | **0.000675621** | **-1.40365** | **0.00971106** | **-1.25407** | **2.8255e-005** | **-1.64131** |
| **adenylosuccinate lyase** | **ADSL** | **0.000153301** | **-1.42952** | **0.0143659** | **-1.20233** | **9.27671e-005** | **-1.461** |
| **v-Ki-ras2 Kirsten rat sarcoma viral oncogene homolog** | **KRAS** | **0.00011014** | **-1.47219** | **0.00619432** | **-1.24978** | **4.04194e-005** | **-1.5431** |
| **DTW domain containing 1** | **DTWD1** | **0.000360519** | **-1.48408** | **0.000112704** | **-1.57563** | **6.88753e-005** | **-1.61831** |
| **chromosome 16 open reading frame 13** | **C16orf13** | **0.000628825** | **-1.51022** | **0.00527618** | **-1.35351** | **2.44013e-005** | **-1.83397** |
| **tumor protein p53 (Li-Fraumeni syndrome)** | **TP53** | **0.000833372** | **-1.54091** | **0.0018592** | **-1.47216** | **7.78129e-005** | **-1.7853** |
| **vacuolar protein sorting 16 homolog (S. cerevisiae)** | **VPS16** | **0.000222313** | **-1.55078** | **0.00285759** | **-1.36495** | **7.32408e-005** | **-1.64929** |
| **zinc finger and BTB domain containing 10** | **ZBTB10** | **0.000178796** | **-1.55446** | **0.000133302** | **-1.57913** | **9.16072e-005** | **-1.61193** |
| **Pentatricopeptide repeat domain 3** | **PTCD3** | **0.000559188** | **-1.59984** | **0.000383149** | **-1.63759** | **4.89072e-005** | **-1.8778** |
| **tRNA aspartic acid methyltransferase 1** | **TRDMT1** | **0.000225094** | **-1.61157** | **0.00182186** | **-1.43648** | **4.62733e-005** | **-1.77579** |
| **Chromosome 16 open reading frame 13** | **C16orf13** | **0.000139286** | **-1.61969** | **0.00254313** | **-1.38951** | **8.45243e-005** | **-1.66747** |
| **protein phosphatase 1F (PP2C domain containing)** | **PPM1F** | **0.000117138** | **-1.64069** | **0.000321285** | **-1.54987** | **1.8961e-005** | **-1.83692** |
| **KIAA1856 protein** | **KIAA1856** | **0.000350735** | **-1.64862** | **0.000223852** | **-1.69641** | **5.79939e-005** | **-1.85804** |
| **diacylglycerol lipase, beta** | **DAGLB** | **0.00054065** | **-1.67619** | **0.0031234** | **-1.49782** | **4.41611e-005** | **-2.00884** |
| **negative regulator of ubiquitin-like proteins 1** | **NUB1** | **0.000155931** | **-1.67629** | **0.000373989** | **-1.59043** | **6.53521e-005** | **-1.77149** |
| **DnaJ (Hsp40) homolog, subfamily C, member 19** | **DNAJC19** | **0.000111525** | **-1.68101** | **0.00231531** | **-1.41903** | **6.80327e-005** | **-1.7333** |
| **hypothetical protein FLJ11184** | **FLJ11184** | **0.00011848** | **-1.68158** | **0.00426566** | **-1.37875** | **9.27861e-005** | **-1.70713** |
| **low density lipoprotein receptor-related protein 5-like** | **LRP5L** | **0.000346699** | **-1.75272** | **0.000145278** | **-1.86652** | **2.38093e-005** | **-2.15317** |
| **mitochondrial ribosomal protein L19** | **MRPL19** | **0.000265737** | **-1.76633** | **0.00231705** | **-1.53032** | **7.0312e-005** | **-1.94689** |
| **retinoblastoma binding protein 9** | **RBBP9** | **0.00029216** | **-1.76661** | **0.00708636** | **-1.43435** | **4.54095e-005** | **-2.03118** |
| **KIAA0500 protein** | **KIAA0500** | **0.000418227** | **-1.76948** | **0.000113908** | **-1.95198** | **2.28955e-005** | **-2.23018** |
| **chromosome 11 open reading frame 49** | **C11orf49** | **0.000493929** | **-1.83712** | **0.000118666** | **-2.064** | **8.19125e-005** | **-2.13115** |
| **component of oligomeric golgi complex 6** | **COG6** | **0.000292231** | **-1.85608** | **0.000300989** | **-1.85188** | **4.10064e-005** | **-2.17935** |
| **protein tyrosine phosphatase, non-receptor type 18 (brain-derived)** | **PTPN18** | **0.000189155** | **-1.85777** | **0.000857857** | **-1.66712** | **5.05347e-005** | **-2.05962** |
| **polymerase (DNA directed), beta** | **POLB** | **0.000105843** | **-1.87095** | **0.000474646** | **-1.68444** | **8.59056e-005** | **-1.90001** |
| **histamine N-methyltransferase** | **HNMT** | **0.000539328** | **-1.90366** | **0.00105801** | **-1.8014** | **8.4766e-005** | **-2.24087** |
| **Pellino homolog 1 (Drosophila)** | **PELI1** | **0.000140615** | **-1.91299** | **0.00015842** | **-1.89556** | **4.70314e-005** | **-2.08803** |
| **mannosidase, endo-alpha** | **MANEA** | **0.000223161** | **-1.93595** | **0.000476178** | **-1.82368** | **2.5023e-005** | **-2.34029** |
| **yippee-like 2 (Drosophila)** | **YPEL2** | **0.000109801** | **-1.98366** | **0.000685239** | **-1.7261** | **2.07535e-005** | **-2.28573** |
| **Ets variant gene 5 (ets-related molecule)** | **ETV5** | **0.000132851** | **-2.01839** | **0.00143594** | **-1.6779** | **1.79005e-005** | **-2.4132** |
| **SAC3 domain containing 1** | **SAC3D1** | **0.000383789** | **-2.10026** | **0.00108627** | **-1.91014** | **5.08565e-005** | **-2.57028** |
| **TatD DNase domain containing 3** | **TATDN3** | **0.000636731** | **-2.10464** | **0.000875369** | **-2.04076** | **9.42803e-005** | **-2.56349** |
| **chromosome 1 open reading frame 25** | **C1orf25** | **0.000108718** | **-2.16912** | **0.000404402** | **-1.93458** | **5.4902e-005** | **-2.3112** |
| **methylmalonyl CoA epimerase** | **MCEE** | **0.000101221** | **-2.21849** | **0.000719507** | **-1.86819** | **7.73573e-005** | **-2.27521** |
| **thiosulfate sulfurtransferase (rhodanese)** | **TST** | **0.000101609** | **-2.22314** | **0.000742554** | **-1.86689** | **9.70013e-005** | **-2.2328** |
| **leucine rich repeat containing 8 family, member D** | **LRRC8D** | **0.000173694** | **-2.27905** | **0.00345603** | **-1.73437** | **9.51933e-005** | **-2.42175** |
| **nuclear factor (erythroid-derived 2)-like 3** | **NFE2L3** | **0.000269841** | **-2.50522** | **0.0001574** | **-2.66586** | **6.17596e-005** | **-2.9851** |
| **hypothetical protein LOC254128** | **LOC254128** | **0.000211596** | **-2.52421** | **0.00726628** | **-1.75363** | **5.77644e-005** | **-2.94006** |
| **chloride channel 4** | **CLCN4** | **0.000117227** | **-2.87136** | **0.000232231** | **-2.64259** | **5.67326e-005** | **-3.14898** |
| **leucine-rich repeat kinase 2** | **LRRK2** | **0.000111713** | **-3.88733** | **0.00051427** | **-3.08211** | **7.7878e-005** | **-4.12024** |
| **SIN3 homolog B, transcription regulator (yeast)** | **SIN3B** | **5.33041e-005** | **-1.68848** | **2.37688e-005** | **-1.77385** | **4.24843e-005** | **-1.71155** |
| **kelch-like 22 (Drosophila)** | **KLHL22** | **6.7966e-006** | **-1.82433** | **2.3959e-005** | **-1.69069** | **3.66153e-006** | **-1.89898** |
| **xylosylprotein beta 1,4-galactosyltransferase, polypeptide 7 (galactosyltransfer** | **B4GALT7** | **5.14557e-006** | **-1.62228** | **7.04672e-006** | **-1.59701** | **9.42915e-006** | **-1.57447** |
| **GTPase, IMAP family member 5** | **GIMAP5** | **1.91106e-006** | **-2.69185** | **8.05723e-006** | **-2.34632** | **2.79146e-006** | **-2.59226** |
| **heat shock 70kDa protein 1A** | **HSPA1A** | **4.04919e-006** | **3.19971** | **2.58489e-005** | **2.59699** | **4.96888e-006** | **3.12221** |
| **glutamyl-prolyl-tRNA synthetase** | **EPRS** | **2.22051e-006** | **-2.03641** | **5.5913e-005** | **-1.65406** | **8.06808e-006** | **-1.86246** |
| **muscleblind-like (Drosophila)** | **MBNL1** | **1.26183e-005** | **-1.25371** | **8.8948e-005** | **-1.19954** | **3.21557e-005** | **-1.22652** |
| **threonyl-tRNA synthetase** | **TARS** | **6.77111e-006** | **-1.34333** | **5.73587e-005** | **-1.26336** | **6.60454e-006** | **-1.34437** |
| **thioredoxin reductase 1** | **TXNRD1** | **5.75177e-006** | **1.33681** | **6.15629e-006** | **1.33406** | **2.27237e-006** | **1.37657** |
| **WD repeat domain 77** | **WDR77** | **2.64586e-006** | **-1.92746** | **4.93823e-005** | **-1.61517** | **7.30431e-006** | **-1.8047** |
| **aldehyde dehydrogenase 2 family (mitochondrial)** | **ALDH2** | **5.09854e-006** | **-1.83693** | **2.06008e-005** | **-1.6887** | **4.74791e-006** | **-1.84528** |
| **methionyl-tRNA synthetase** | **MARS** | **1.04981e-006** | **-1.65698** | **3.02964e-005** | **-1.42576** | **6.35634e-007** | **-1.70072** |
| **NADH dehydrogenase (ubiquinone) Fe-S protein 3, 30kDa (NADH-coenzyme Q reductase** | **NDUFS3** | **3.86571e-007** | **-1.59237** | **1.31216e-005** | **-1.38264** | **8.56046e-007** | **-1.53694** |
| **heat shock 70kDa protein 1B** | **HSPA1B** | **3.09624e-005** | **7.38214** | **9.13219e-005** | **5.8615** | **6.40924e-005** | **6.3093** |
| **chromosome 10 open reading frame 26** | **C10orf26** | **3.27192e-006** | **-1.90532** | **8.40474e-005** | **-1.57307** | **7.81656e-006** | **-1.80146** |
| **phosphoenolpyruvate carboxykinase 2 (mitochondrial)** | **PCK2** | **3.40237e-006** | **-2.08236** | **2.59595e-005** | **-1.80551** | **5.10038e-006** | **-2.02038** |
| **solute carrier family 35 (CMP-sialic acid transporter), member A1** | **SLC35A1** | **8.146e-006** | **-2.1606** | **3.54641e-005** | **-1.9286** | **1.08084e-005** | **-2.11187** |
| **inositol polyphosphate-5-phosphatase, 145kDa** | **INPP5D** | **1.32038e-006** | **-1.84807** | **7.42774e-006** | **-1.67169** | **1.16752e-006** | **-1.86233** |
| **phospholipase C, beta 2** | **PLCB2** | **1.7885e-005** | **-1.72** | **5.43495e-005** | **-1.6152** | **3.08817e-006** | **-1.92112** |
| **IMP (inosine monophosphate) dehydrogenase 1** | **IMPDH1** | **9.01169e-006** | **-1.76911** | **1.55766e-005** | **-1.71259** | **5.52973e-006** | **-1.82324** |
| **metallothionein 1G** | **MT1G** | **1.43444e-006** | **2.73118** | **6.48199e-006** | **2.36309** | **4.16245e-007** | **3.11839** |
| **neutrophil cytosolic factor 1, (chronic granulomatous disease, autosomal 1) ///** | **NCF1 /// NCF1B /// NCF1C** | **1.15793e-005** | **-2.21649** | **6.87058e-005** | **-1.92274** | **9.55318e-006** | **-2.25337** |
| **G1 to S phase transition 2** | **GSPT2** | **5.31504e-005** | **-1.88355** | **6.51084e-005** | **-1.85652** | **7.25247e-005** | **-1.84246** |
| **Janus kinase 2 (a protein tyrosine kinase)** | **JAK2** | **2.8929e-005** | **-2.25048** | **6.75267e-005** | **-2.09052** | **1.48982e-005** | **-2.39186** |
| **Janus kinase 2 (a protein tyrosine kinase)** | **JAK2** | **8.25899e-006** | **-2.00531** | **5.19018e-005** | **-1.76688** | **1.37338e-005** | **-1.93294** |
| **metallothionein 1H /// metallothionein 1 pseudogene 2** | **MT1H /// MT1P2** | **1.17455e-005** | **2.71422** | **4.00175e-005** | **2.39465** | **3.2461e-006** | **3.13598** |
| **solute carrier family 16, member 6 (monocarboxylic acid transporter 7)** | **SLC16A6** | **1.61712e-005** | **1.70478** | **9.61308e-006** | **1.75775** | **1.5201e-005** | **1.71089** |
| **zinc finger, MYM-type 3** | **ZMYM3** | **3.70144e-005** | **-2.3558** | **8.88423e-005** | **-2.17207** | **2.12021e-005** | **-2.48737** |
| **eukaryotic translation initiation factor 5** | **EIF5** | **1.76321e-005** | **1.34146** | **5.88171e-005** | **1.29302** | **6.99009e-006** | **1.38305** |
| **septin 8** | **SEPT8** | **6.68299e-007** | **-1.72004** | **5.64282e-006** | **-1.54677** | **2.42036e-007** | **-1.82165** |
| **iron-sulfur cluster scaffold homolog (E. coli)** | **ISCU** | **5.90789e-006** | **-1.52907** | **4.1835e-005** | **-1.41001** | **1.09868e-005** | **-1.48836** |
| **spectrin repeat containing, nuclear envelope 1** | **SYNE1** | **1.67843e-005** | **-3.21344** | **6.32101e-005** | **-2.73866** | **2.26905e-005** | **-3.09455** |
| **butyrophilin, subfamily 3, member A1** | **BTN3A1** | **2.71048e-005** | **-2.07992** | **1.88529e-005** | **-2.1426** | **3.28189e-006** | **-2.50149** |
| **chromosome 16 open reading frame 35** | **C16orf35** | **1.0822e-005** | **-1.8857** | **9.03119e-005** | **-1.65067** | **2.30187e-005** | **-1.79427** |
| **general transcription factor II, i /// general transcription factor II, i, pseud** | **GTF2I /// GTF2IP1 /// LOC100093631 /// LOC732437** | **2.9882e-006** | **-1.62235** | **4.74549e-005** | **-1.43275** | **7.57187e-006** | **-1.5514** |
| **regulator of G-protein signaling 14** | **RGS14** | **7.56093e-006** | **1.84291** | **7.94038e-005** | **1.60316** | **4.18864e-005** | **1.66128** |
| **exportin 6** | **XPO6** | **2.39332e-006** | **-1.60857** | **5.18828e-005** | **-1.40752** | **4.88687e-006** | **-1.55507** |
| **tyrosyl-tRNA synthetase** | **YARS** | **4.63829e-008** | **-1.56302** | **1.731e-007** | **-1.48152** | **4.52265e-008** | **-1.56472** |
| **transcription factor 4** | **TCF4** | **3.50014e-007** | **-2.63559** | **2.03828e-007** | **-2.77895** | **4.05662e-007** | **-2.59884** |
| **absent in melanoma 1** | **AIM1** | **5.7005e-007** | **-3.0199** | **1.90258e-005** | **-2.1565** | **2.02807e-006** | **-2.64452** |
| **butyrophilin, subfamily 3, member A2** | **BTN3A2** | **3.21297e-006** | **-1.95226** | **6.94575e-005** | **-1.61555** | **6.59522e-006** | **-1.86022** |
| **lipoma HMGIC fusion partner-like 2** | **LHFPL2** | **7.77636e-006** | **1.35938** | **5.3374e-005** | **1.28247** | **7.62438e-006** | **1.36025** |
| **metallothionein 1E** | **MT1E** | **8.88948e-006** | **3.42852** | **2.39768e-005** | **3.02558** | **2.1035e-006** | **4.18496** |
| **TRAF2 and NCK interacting kinase** | **TNIK** | **3.3074e-005** | **-2.0177** | **2.46429e-005** | **-2.06502** | **1.94717e-005** | **-2.10446** |
| **DAZ interacting protein 3, zinc finger** | **DZIP3** | **1.08495e-005** | **-1.50443** | **1.16721e-005** | **-1.49965** | **2.99308e-005** | **-1.44165** |
| **exonuclease domain containing 1** | **EXOD1** | **1.17144e-005** | **-2.55093** | **6.53997e-005** | **-2.16949** | **2.4304e-005** | **-2.37562** |
| **DEAH (Asp-Glu-Ala-Asp/His) box polypeptide 57** | **DHX57** | **9.30207e-005** | **-1.66834** | **6.6092e-005** | **-1.70303** | **1.66546e-005** | **-1.85998** |
| **melanocortin 1 receptor (alpha melanocyte stimulating hormone receptor) /// tubu** | **MC1R /// TUBB3** | **2.9909e-005** | **1.49966** | **7.25056e-005** | **1.44305** | **1.91278e-005** | **1.53052** |
| **methionyl-tRNA synthetase** | **MARS** | **2.7659e-007** | **-1.77022** | **2.54558e-006** | **-1.57937** | **3.49956e-007** | **-1.74718** |
| **similar to Neutrophil cytosol factor 1 (NCF-1) (Neutrophil NADPH oxidase factor** | **LOC648998 /// LOC652625 /// LOC652699 /// NCF1 /// NCF1B /// NCF1C** | **2.73369e-005** | **-1.91578** | **9.76344e-005** | **-1.75568** | **1.86539e-005** | **-1.96973** |
| **nuclear factor (erythroid-derived 2)-like 1** | **NFE2L1** | **1.2057e-006** | **-1.57462** | **4.70609e-005** | **-1.36051** | **6.89064e-006** | **-1.46137** |
| **mitogen-activated protein kinase kinase 3** | **MAP2K3** | **1.19303e-006** | **1.58313** | **8.38923e-006** | **1.45584** | **5.75003e-006** | **1.47821** |
| **metallothionein 1M** | **MT1M** | **1.33632e-005** | **2.92983** | **9.45382e-005** | **2.37299** | **7.80804e-006** | **3.12147** |
| **1-acylglycerol-3-phosphate O-acyltransferase 5 (lysophosphatidic acid acyltransf** | **AGPAT5** | **9.67057e-007** | **-1.95416** | **1.86606e-006** | **-1.87166** | **1.24934e-006** | **-1.92107** |
| **GTPase, IMAP family member 5** | **GIMAP5** | **1.38202e-005** | **-2.39015** | **9.96327e-005** | **-2.01121** | **1.04846e-005** | **-2.45352** |
| **zinc finger protein 64 homolog (mouse)** | **ZFP64** | **2.90682e-006** | **-2.58207** | **2.26597e-005** | **-2.1449** | **1.90131e-006** | **-2.6933** |
| **polymerase (RNA) III (DNA directed) polypeptide B** | **POLR3B** | **4.24104e-005** | **-2.08883** | **8.48596e-006** | **-2.4052** | **4.45527e-005** | **-2.08035** |
| **receptor (chemosensory) transporter protein 4** | **RTP4** | **3.24366e-005** | **-2.11816** | **3.10444e-005** | **-2.12593** | **3.56911e-005** | **-2.10142** |
| **nucleotide-binding oligomerization domain containing 2** | **NOD2** | **3.23249e-005** | **-2.36604** | **9.92379e-006** | **-2.66198** | **1.90858e-005** | **-2.49063** |
| **apolipoprotein L, 3** | **APOL3** | **3.26057e-007** | **-3.98953** | **6.63807e-006** | **-2.76969** | **7.12461e-007** | **-3.59772** |
| **angiogenic factor with G patch and FHA domains 1** | **AGGF1** | **4.73951e-007** | **-1.8658** | **7.01112e-006** | **-1.60606** | **2.52388e-006** | **-1.69363** |
| **egl nine homolog 3 (C. elegans)** | **EGLN3** | **1.20388e-005** | **5.00129** | **7.83055e-005** | **3.69826** | **8.67784e-006** | **5.29545** |
| **dual adaptor of phosphotyrosine and 3-phosphoinositides** | **DAPP1** | **2.69226e-007** | **-3.06331** | **8.69653e-007** | **-2.70848** | **4.79581e-007** | **-2.87884** |
| **nudix (nucleoside diphosphate linked moiety X)-type motif 5** | **NUDT5** | **7.3624e-008** | **-1.71738** | **5.63294e-005** | **-1.3135** | **3.8611e-006** | **-1.43957** |
| **chromosome Y open reading frame 15B** | **CYorf15B** | **4.36445e-005** | **-1.73676** | **6.95915e-006** | **-1.96169** | **1.07079e-005** | **-1.90353** |
| **reticulon 4 interacting protein 1** | **RTN4IP1** | **1.55335e-005** | **-2.39486** | **7.9822e-005** | **-2.07027** | **2.15702e-005** | **-2.32269** |
| **ankyrin repeat domain 13A** | **ANKRD13A** | **1.00767e-006** | **-1.46089** | **4.36854e-005** | **-1.28987** | **4.1862e-007** | **-1.51254** |
| **RAB3D, member RAS oncogene family** | **RAB3D** | **8.7819e-007** | **-3.07352** | **3.92713e-006** | **-2.62158** | **1.27294e-006** | **-2.9494** |
| **pyruvate dehydrogenase kinase, isozyme 4** | **PDK4** | **2.08539e-006** | **-3.58237** | **5.72871e-006** | **-3.15622** | **2.07233e-006** | **-3.58531** |
| **zinc finger protein 652** | **ZNF652** | **3.81598e-005** | **-2.4152** | **7.28709e-005** | **-2.26932** | **1.24551e-005** | **-2.70918** |
| **anaphase promoting complex subunit 7** | **ANAPC7** | **7.3006e-006** | **-1.7921** | **6.8171e-005** | **-1.57881** | **4.80985e-005** | **-1.60823** |
| **chromosome 14 open reading frame 101** | **C14orf101** | **7.11215e-006** | **-1.61745** | **5.78895e-005** | **-1.46586** | **5.23522e-005** | **-1.4723** |
| **hypothetical protein LOC124512** | **LOC124512** | **2.11985e-005** | **-1.53358** | **7.02197e-005** | **-1.45354** | **1.91069e-005** | **-1.54107** |
| **KIAA1450 protein** | **KIAA1450** | **5.99548e-006** | **1.56314** | **1.18801e-005** | **1.51509** | **1.49131e-006** | **1.67464** |
| **OMA1 homolog, zinc metallopeptidase (S. cerevisiae)** | **OMA1** | **5.44079e-005** | **-2.45067** | **9.23327e-005** | **-2.32463** | **3.46174e-005** | **-2.56781** |
| **disrupted in renal carcinoma 2** | **DIRC2** | **7.56847e-005** | **-2.38098** | **8.79886e-005** | **-2.3456** | **1.54732e-005** | **-2.8157** |
| **mannosyl (alpha-1,3-)-glycoprotein beta-1,4-N-acetylglucosaminyltransferase, iso** | **MGAT4A** | **1.60131e-005** | **-2.76835** | **7.9663e-005** | **-2.34298** | **1.05294e-005** | **-2.90126** |
| **hypothetical protein MGC16169** | **MGC16169** | **3.48732e-005** | **-1.99984** | **4.8904e-005** | **-1.94895** | **1.07027e-005** | **-2.19945** |
| **coiled-coil domain containing 100** | **CCDC100** | **2.46236e-005** | **-1.7936** | **7.12793e-005** | **-1.67959** | **4.47711e-005** | **-1.72751** |
| **KIAA1450 protein** | **KIAA1450** | **1.21179e-006** | **1.64849** | **4.15927e-006** | **1.55358** | **6.35529e-007** | **1.70458** |
| **LysM, putative peptidoglycan-binding, domain containing 2** | **LYSMD2** | **1.37294e-005** | **-2.1895** | **1.01582e-005** | **-2.24647** | **5.01315e-006** | **-2.39154** |
| **IKAROS family zinc finger 1 (Ikaros)** | **IKZF1** | **2.17835e-005** | **-2.9458** | **4.02244e-005** | **-2.74431** | **4.77554e-005** | **-2.69191** |
| **tumor necrosis factor, alpha-induced protein 8-like 1** | **TNFAIP8L1** | **1.54257e-005** | **-2.65107** | **6.10875e-005** | **-2.30954** | **2.01335e-005** | **-2.57846** |
| **progestin and adipoQ receptor family member VIII** | **PAQR8** | **6.62375e-006** | **-3.95232** | **2.40773e-005** | **-3.30853** | **2.1046e-006** | **-4.70074** |
| **hypothetical protein LOC129293** | **LOC129293** | **4.60795e-006** | **-2.5375** | **3.27265e-005** | **-2.12654** | **4.71909e-006** | **-2.53165** |
| **CDNA FLJ38039 fis, clone CTONG2013934** | **---** | **3.91309e-006** | **-2.73452** | **7.68428e-006** | **-2.5532** | **3.93943e-006** | **-2.73261** |
| **Transcribed locus, strongly similar to NP_001028856.1 chaperonin subunit 6a (zet** | **---** | **3.00146e-005** | **-2.08072** | **4.46603e-005** | **-2.01567** | **4.96256e-005** | **-1.99905** |
| **Transcribed locus** | **---** | **3.16463e-005** | **-2.53832** | **1.29188e-005** | **-2.79298** | **6.0226e-006** | **-3.04575** |
| **chromosome 20 open reading frame 19** | **C20orf19** | **3.66585e-006** | **-3.02642** | **1.21e-005** | **-2.65532** | **1.71966e-005** | **-2.56066** |
| **chromosome 1 open reading frame 162** | **C1orf162** | **9.6754e-006** | **-1.70515** | **1.58207e-005** | **-1.65905** | **1.97809e-006** | **-1.87676** |
| **transmembrane protein 201** | **TMEM201** | **8.54671e-006** | **-1.60454** | **6.89482e-005** | **-1.45625** | **1.95537e-005** | **-1.5413** |
| **Transcribed locus** | **---** | **1.61075e-006** | **-3.95188** | **1.53591e-005** | **-2.9613** | **5.07942e-006** | **-3.3903** |
| **chromosome 12 open reading frame 26** | **C12orf26** | **7.17069e-006** | **-2.44958** | **2.15376e-005** | **-2.21713** | **6.24986e-005** | **-2.02785** |
| **yippee-like 2 (Drosophila)** | **YPEL2** | **4.83384e-006** | **-3.08365** | **2.80372e-005** | **-2.54171** | **6.71927e-006** | **-2.96809** |
| **CDNA FLJ45384 fis, clone BRHIP3021987** | **---** | **1.86004e-006** | **-3.48992** | **5.29917e-006** | **-3.07061** | **1.60505e-006** | **-3.5565** |
| **Transcribed locus, strongly similar to NP_038703.3 spectrin beta 1 [Mus musculus** | **---** | **1.83172e-006** | **-2.55648** | **1.70244e-006** | **-2.5745** | **1.05393e-006** | **-2.69849** |
| **family with sequence similarity 118, member B** | **FAM118B** | **8.73127e-006** | **-1.64224** | **1.03681e-005** | **-1.62755** | **1.06009e-005** | **-1.62568** |
| **mannosyl (alpha-1,3-)-glycoprotein beta-1,4-N-acetylglucosaminyltransferase, iso** | **MGAT4A** | **5.86105e-005** | **-3.93799** | **5.69254e-005** | **-3.95597** | **3.75072e-005** | **-4.2265** |
| **Sep (O-phosphoserine) tRNA:Sec (selenocysteine) tRNA synthase** | **SEPSECS** | **1.1781e-005** | **-2.07674** | **1.41553e-005** | **-2.04732** | **1.08527e-005** | **-2.09016** |
| **GTPase, IMAP family member 2** | **GIMAP2** | **3.65785e-006** | **-4.29345** | **2.67514e-005** | **-3.2499** | **6.98384e-006** | **-3.90368** |
| **serine/threonine kinase 36, fused homolog (Drosophila)** | **STK36** | **1.76707e-005** | **-2.15723** | **6.51252e-005** | **-1.94444** | **1.54158e-005** | **-2.18207** |
| **cerebellin 3 precursor** | **CBLN3** | **6.25818e-005** | **-2.4795** | **3.54305e-005** | **-2.63317** | **2.35268e-005** | **-2.75382** |
| **TIFA-related protein TIFAB** | **TIFAB** | **4.9986e-007** | **-3.3115** | **4.19723e-005** | **-2.12341** | **9.18266e-007** | **-3.08577** |
| **chromosome 5 open reading frame 20 /// TIFA-related protein TIFAB** | **C5orf20 /// TIFAB** | **6.55931e-006** | **-4.02275** | **1.97697e-005** | **-3.44529** | **2.89791e-006** | **-4.55145** |
| **serum response factor binding protein 1** | **SRFBP1** | **9.30096e-006** | **-1.78102** | **5.79566e-005** | **-1.60355** | **1.0215e-005** | **-1.77085** |
| **GTPase, IMAP family member 1** | **GIMAP1** | **1.33551e-006** | **-2.62547** | **1.2611e-006** | **-2.64026** | **1.00347e-006** | **-2.70071** |
| **interleukin 12 receptor, beta 1** | **IL12RB1** | **5.51652e-007** | **-1.51672** | **2.12815e-005** | **-1.33001** | **1.48212e-006** | **-1.45817** |
| **acetyl-Coenzyme A carboxylase beta** | **ACACB** | **1.90697e-006** | **8.78959** | **4.4918e-006** | **7.31523** | **2.15842e-006** | **8.55269** |
| **CDNA FLJ38785 fis, clone LIVER2001329** | **---** | **9.3759e-007** | **-2.50113** | **1.95886e-006** | **-2.34159** | **7.28244e-007** | **-2.56066** |
| **CDNA FLJ30303 fis, clone BRACE2003269** | **---** | **5.04444e-005** | **-2.29359** | **4.68566e-005** | **-2.30957** | **3.8718e-005** | **-2.35178** |
| **M-phase phosphoprotein 9** | **MPHOSPH9** | **1.06633e-005** | **-3.07952** | **9.30507e-005** | **-2.42116** | **6.16283e-005** | **-2.52744** |
| **signal-regulatory protein beta 2** | **SIRPB2** | **5.56164e-005** | **-1.66667** | **5.85046e-005** | **-1.66179** | **7.33395e-006** | **-1.89135** |
| **MRNA; cDNA DKFZp434N0220 (from clone DKFZp434N0220)** | **---** | **1.52679e-006** | **-4.05143** | **2.61621e-006** | **-3.75923** | **1.26574e-006** | **-4.1616** |
| **Homo sapiens, Similar to hypothetical protein FLJ20378, clone IMAGE:4179392, mRN** | **---** | **1.71992e-005** | **-2.33174** | **2.82472e-005** | **-2.22978** | **8.02945e-005** | **-2.03961** |
| **acetyl-Coenzyme A carboxylase beta** | **ACACB** | **1.90697e-006** | **8.78959** | **4.4918e-006** | **7.31523** | **2.15842e-006** | **8.55269** |
| **heat shock 70kDa protein 1B** | **HSPA1B** | **3.09624e-005** | **7.38214** | **9.13219e-005** | **5.8615** | **6.40924e-005** | **6.3093** |
| **egl nine homolog 3 (C. elegans)** | **EGLN3** | **1.20388e-005** | **5.00129** | **7.83055e-005** | **3.69826** | **8.67784e-006** | **5.29545** |
| **metallothionein 1E** | **MT1E** | **8.88948e-006** | **3.42852** | **2.39768e-005** | **3.02558** | **2.1035e-006** | **4.18496** |
| **heat shock 70kDa protein 1A** | **HSPA1A** | **4.04919e-006** | **3.19971** | **2.58489e-005** | **2.59699** | **4.96888e-006** | **3.12221** |
| **metallothionein 1M** | **MT1M** | **1.33632e-005** | **2.92983** | **9.45382e-005** | **2.37299** | **7.80804e-006** | **3.12147** |
| **metallothionein 1G** | **MT1G** | **1.43444e-006** | **2.73118** | **6.48199e-006** | **2.36309** | **4.16245e-007** | **3.11839** |
| **metallothionein 1H /// metallothionein 1 pseudogene 2** | **MT1H /// MT1P2** | **1.17455e-005** | **2.71422** | **4.00175e-005** | **2.39465** | **3.2461e-006** | **3.13598** |
| **regulator of G-protein signaling 14** | **RGS14** | **7.56093e-006** | **1.84291** | **7.94038e-005** | **1.60316** | **4.18864e-005** | **1.66128** |
| **solute carrier family 16, member 6 (monocarboxylic acid transporter 7)** | **SLC16A6** | **1.61712e-005** | **1.70478** | **9.61308e-006** | **1.75775** | **1.5201e-005** | **1.71089** |
| **KIAA1450 protein** | **KIAA1450** | **1.21179e-006** | **1.64849** | **4.15927e-006** | **1.55358** | **6.35529e-007** | **1.70458** |
| **mitogen-activated protein kinase kinase 3** | **MAP2K3** | **1.19303e-006** | **1.58313** | **8.38923e-006** | **1.45584** | **5.75003e-006** | **1.47821** |
| **KIAA1450 protein** | **KIAA1450** | **5.99548e-006** | **1.56314** | **1.18801e-005** | **1.51509** | **1.49131e-006** | **1.67464** |
| **melanocortin 1 receptor (alpha melanocyte stimulating hormone receptor) /// tubu** | **MC1R /// TUBB3** | **2.9909e-005** | **1.49966** | **7.25056e-005** | **1.44305** | **1.91278e-005** | **1.53052** |
| **lipoma HMGIC fusion partner-like 2** | **LHFPL2** | **7.77636e-006** | **1.35938** | **5.3374e-005** | **1.28247** | **7.62438e-006** | **1.36025** |
| **eukaryotic translation initiation factor 5** | **EIF5** | **1.76321e-005** | **1.34146** | **5.88171e-005** | **1.29302** | **6.99009e-006** | **1.38305** |
| **hioredoxin reductase 1** | **TXNRD1** | **5.75177e-006** | **1.33681** | **6.15629e-006** | **1.33406** | **2.27237e-006** | **1.37657** |
| **phospholipase C, beta 2** | **PLCB2** | **1.7885e-005** | **-1.72** | **5.43495e-005** | **-1.6152** | **3.08817e-006** | **-1.92112** |
| **anaphase promoting complex subunit 7** | **ANAPC7** | **7.3006e-006** | **-1.7921** | **6.8171e-005** | **-1.57881** | **4.80985e-005** | **-1.60823** |
| **coiled-coil domain containing 100** | **CCDC100** | **2.46236e-005** | **-1.7936** | **7.12793e-005** | **-1.67959** | **4.47711e-005** | **-1.72751** |
| **angiogenic factor with G patch and FHA domains 1** | **AGGF1** | **4.73951e-007** | **-1.8658** | **7.01112e-006** | **-1.60606** | **2.52388e-006** | **-1.69363** |
| **chromosome 16 open reading frame 35** | **C16orf35** | **1.0822e-005** | **-1.8857** | **9.03119e-005** | **-1.65067** | **2.30187e-005** | **-1.79427** |
| **TRAF2 and NCK interacting kinase** | **TNIK** | **3.3074e-005** | **-2.0177** | **2.46429e-005** | **-2.06502** | **1.94717e-005** | **-2.10446** |
| **solute carrier family 35 (CMP-sialic acid transporter), member A1** | **SLC35A1** | **8.146e-006** | **-2.1606** | **3.54641e-005** | **-1.9286** | **1.08084e-005** | **-2.11187** |
| **LysM, putative peptidoglycan-binding, domain containing 2** | **LYSMD2** | **1.37294e-005** | **-2.1895** | **1.01582e-005** | **-2.24647** | **5.01315e-006** | **-2.39154** |
| **zinc finger, MYM-type 3** | **ZMYM3** | **3.70144e-005** | **-2.3558** | **8.88423e-005** | **-2.17207** | **2.12021e-005** | **-2.48737** |
| **zinc finger protein 652** | **ZNF652** | **3.81598e-005** | **-2.4152** | **7.28709e-005** | **-2.26932** | **1.24551e-005** | **-2.70918** |
| **cerebellin 3 precursor** | **CBLN3** | **6.25818e-005** | **-2.4795** | **3.54305e-005** | **-2.63317** | **2.35268e-005** | **-2.75382** |
| **hypothetical protein LOC129293** | **LOC129293** | **4.60795e-006** | **-2.5375** | **3.27265e-005** | **-2.12654** | **4.71909e-006** | **-2.53165** |
| **IKAROS family zinc finger 1 (Ikaros)** | **IKZF1** | **2.17835e-005** | **-2.9458** | **4.02244e-005** | **-2.74431** | **4.77554e-005** | **-2.69191** |
| **absent in melanoma 1** | **AIM1** | **5.7005e-007** | **-3.0199** | **1.90258e-005** | **-2.1565** | **2.02807e-006** | **-2.64452** |
| **TIFA-related protein TIFAB** | **TIFAB** | **4.9986e-007** | **-3.3115** | **4.19723e-005** | **-2.12341** | **9.18266e-007** | **-3.08577** |
| **muscleblind-like (Drosophila)** | **MBNL1** | **1.26183e-005** | **-1.25371** | **8.8948e-005** | **-1.19954** | **3.21557e-005** | **-1.22652** |
| **threonyl-tRNA synthetase** | **TARS** | **6.77111e-006** | **-1.34333** | **5.73587e-005** | **-1.26336** | **6.60454e-006** | **-1.34437** |
| **ankyrin repeat domain 13A** | **ANKRD13A** | **1.00767e-006** | **-1.46089** | **4.36854e-005** | **-1.28987** | **4.1862e-007** | **-1.51254** |
| **DAZ interacting protein 3, zinc finger** | **DZIP3** | **1.08495e-005** | **-1.50443** | **1.16721e-005** | **-1.49965** | **2.99308e-005** | **-1.44165** |
| **interleukin 12 receptor, beta 1** | **IL12RB1** | **5.51652e-007** | **-1.51672** | **2.12815e-005** | **-1.33001** | **1.48212e-006** | **-1.45817** |
| **iron-sulfur cluster scaffold homolog (E. coli)** | **ISCU** | **5.90789e-006** | **-1.52907** | **4.1835e-005** | **-1.41001** | **1.09868e-005** | **-1.48836** |
| **hypothetical protein LOC124512** | **LOC124512** | **2.11985e-005** | **-1.53358** | **7.02197e-005** | **-1.45354** | **1.91069e-005** | **-1.54107** |
| **tyrosyl-tRNA synthetase** | **YARS** | **4.63829e-008** | **-1.56302** | **1.731e-007** | **-1.48152** | **4.52265e-008** | **-1.56472** |
| **nuclear factor (erythroid-derived 2)-like 1** | **NFE2L1** | **1.2057e-006** | **-1.57462** | **4.70609e-005** | **-1.36051** | **6.89064e-006** | **-1.46137** |
| **NADH dehydrogenase (ubiquinone) Fe-S protein 3, 30kDa (NADH-coenzyme Q reductase** | **NDUFS3** | **3.86571e-007** | **-1.59237** | **1.31216e-005** | **-1.38264** | **8.56046e-007** | **-1.53694** |
| **transmembrane protein 201** | **TMEM201** | **8.54671e-006** | **-1.60454** | **6.89482e-005** | **-1.45625** | **1.95537e-005** | **-1.5413** |
| **exportin 6** | **XPO6** | **2.39332e-006** | **-1.60857** | **5.18828e-005** | **-1.40752** | **4.88687e-006** | **-1.55507** |
| **chromosome 14 open reading frame 101** | **C14orf101** | **7.11215e-006** | **-1.61745** | **5.78895e-005** | **-1.46586** | **5.23522e-005** | **-1.4723** |
| **xylosylprotein beta 1,4-galactosyltransferase, polypeptide 7 (galactosyltransfer** | **B4GALT7** | **5.14557e-006** | **-1.62228** | **7.04672e-006** | **-1.59701** | **9.42915e-006** | **-1.57447** |
| **general transcription factor II, i /// general transcription factor II, i, pseud** | **GTF2I /// GTF2IP1 /// LOC100093631 /// LOC732437** | **2.9882e-006** | **-1.62235** | **4.74549e-005** | **-1.43275** | **7.57187e-006** | **-1.5514** |
| **family with sequence similarity 118, member B** | **FAM118B** | **8.73127e-006** | **-1.64224** | **1.03681e-005** | **-1.62755** | **1.06009e-005** | **-1.62568** |
| **methionyl-tRNA synthetase** | **MARS** | **1.04981e-006** | **-1.65698** | **3.02964e-005** | **-1.42576** | **6.35634e-007** | **-1.70072** |
| **signal-regulatory protein beta 2** | **SIRPB2** | **5.56164e-005** | **-1.66667** | **5.85046e-005** | **-1.66179** | **7.33395e-006** | **-1.89135** |
| **DEAH (Asp-Glu-Ala-Asp/His) box polypeptide 57** | **DHX57** | **9.30207e-005** | **-1.66834** | **6.6092e-005** | **-1.70303** | **1.66546e-005** | **-1.85998** |
| **SIN3 homolog B, transcription regulator (yeast)** | **SIN3B** | **5.33041e-005** | **-1.68848** | **2.37688e-005** | **-1.77385** | **4.24843e-005** | **-1.71155** |
| **chromosome 1 open reading frame 162** | **C1orf162** | **9.6754e-006** | **-1.70515** | **1.58207e-005** | **-1.65905** | **1.97809e-006** | **-1.87676** |
| **nudix (nucleoside diphosphate linked moiety X)-type motif 5** | **NUDT5** | **7.3624e-008** | **-1.71738** | **5.63294e-005** | **-1.3135** | **3.8611e-006** | **-1.43957** |
| **septin 8** | **SEPT8** | **6.68299e-007** | **-1.72004** | **5.64282e-006** | **-1.54677** | **2.42036e-007** | **-1.82165** |
| **chromosome Y open reading frame 15B** | **CYorf15B** | **4.36445e-005** | **-1.73676** | **6.95915e-006** | **-1.96169** | **1.07079e-005** | **-1.90353** |
| **IMP (inosine monophosphate) dehydrogenase 1** | **IMPDH1** | **9.01169e-006** | **-1.76911** | **1.55766e-005** | **-1.71259** | **5.52973e-006** | **-1.82324** |
| **methionyl-tRNA synthetase** | **MARS** | **2.7659e-007** | **-1.77022** | **2.54558e-006** | **-1.57937** | **3.49956e-007** | **-1.74718** |
| **serum response factor binding protein 1** | **SRFBP1** | **9.30096e-006** | **-1.78102** | **5.79566e-005** | **-1.60355** | **1.0215e-005** | **-1.77085** |
| **kelch-like 22 (Drosophila)** | **KLHL22** | **6.7966e-006** | **-1.82433** | **2.3959e-005** | **-1.69069** | **3.66153e-006** | **-1.89898** |
| **aldehyde dehydrogenase 2 family (mitochondrial)** | **ALDH2** | **5.09854e-006** | **-1.83693** | **2.06008e-005** | **-1.6887** | **4.74791e-006** | **-1.84528** |
| **inositol polyphosphate-5-phosphatase, 145kDa** | **INPP5D** | **1.32038e-006** | **-1.84807** | **7.42774e-006** | **-1.67169** | **1.16752e-006** | **-1.86233** |
| **G1 to S phase transition 2** | **GSPT2** | **5.31504e-005** | **-1.88355** | **6.51084e-005** | **-1.85652** | **7.25247e-005** | **-1.84246** |
| **chromosome 10 open reading frame 26** | **C10orf26** | **3.27192e-006** | **-1.90532** | **8.40474e-005** | **-1.57307** | **7.81656e-006** | **-1.80146** |
| **similar to Neutrophil cytosol factor 1 (NCF-1) (Neutrophil NADPH oxidase factor** | **LOC648998 /// LOC652625 /// LOC652699 /// NCF1 /// NCF1B /// NCF1C** | **2.73369e-005** | **-1.91578** | **9.76344e-005** | **-1.75568** | **1.86539e-005** | **-1.96973** |
| **WD repeat domain 77** | **WDR77** | **2.64586e-006** | **-1.92746** | **4.93823e-005** | **-1.61517** | **7.30431e-006** | **-1.8047** |
| **butyrophilin, subfamily 3, member A2** | **BTN3A2** | **3.21297e-006** | **-1.95226** | **6.94575e-005** | **-1.61555** | **6.59522e-006** | **-1.86022** |
| **1-acylglycerol-3-phosphate O-acyltransferase 5 (lysophosphatidic acid acyltransf** | **AGPAT5** | **9.67057e-007** | **-1.95416** | **1.86606e-006** | **-1.87166** | **1.24934e-006** | **-1.92107** |
| **hypothetical protein MGC16169** | **MGC16169** | **3.48732e-005** | **-1.99984** | **4.8904e-005** | **-1.94895** | **1.07027e-005** | **-2.19945** |
| **Janus kinase 2 (a protein tyrosine kinase)** | **JAK2** | **8.25899e-006** | **-2.00531** | **5.19018e-005** | **-1.76688** | **1.37338e-005** | **-1.93294** |
| **glutamyl-prolyl-tRNA synthetase** | **EPRS** | **2.22051e-006** | **-2.03641** | **5.5913e-005** | **-1.65406** | **8.06808e-006** | **-1.86246** |
| **Sep (O-phosphoserine) tRNA:Sec (selenocysteine) tRNA synthase** | **SEPSECS** | **1.1781e-005** | **-2.07674** | **1.41553e-005** | **-2.04732** | **1.08527e-005** | **-2.09016** |
| **butyrophilin, subfamily 3, member A1** | **BTN3A1** | **2.71048e-005** | **-2.07992** | **1.88529e-005** | **-2.1426** | **3.28189e-006** | **-2.50149** |
| **Transcribed locus, strongly similar to NP_001028856.1 chaperonin subunit 6a (zet** | **---** | **3.00146e-005** | **-2.08072** | **4.46603e-005** | **-2.01567** | **4.96256e-005** | **-1.99905** |
| **phosphoenolpyruvate carboxykinase 2 (mitochondrial)** | **PCK2** | **3.40237e-006** | **-2.08236** | **2.59595e-005** | **-1.80551** | **5.10038e-006** | **-2.02038** |
| **polymerase (RNA) III (DNA directed) polypeptide B** | **POLR3B** | **4.24104e-005** | **-2.08883** | **8.48596e-006** | **-2.4052** | **4.45527e-005** | **-2.08035** |
| **receptor (chemosensory) transporter protein 4** | **RTP4** | **3.24366e-005** | **-2.11816** | **3.10444e-005** | **-2.12593** | **3.56911e-005** | **-2.10142** |
| **serine/threonine kinase 36, fused homolog (Drosophila)** | **STK36** | **1.76707e-005** | **-2.15723** | **6.51252e-005** | **-1.94444** | **1.54158e-005** | **-2.18207** |
| **neutrophil cytosolic factor 1, (chronic granulomatous disease, autosomal 1) ///** | **NCF1 /// NCF1B /// NCF1C** | **1.15793e-005** | **-2.21649** | **6.87058e-005** | **-1.92274** | **9.55318e-006** | **-2.25337** |
| **Janus kinase 2 (a protein tyrosine kinase)** | **JAK2** | **2.8929e-005** | **-2.25048** | **6.75267e-005** | **-2.09052** | **1.48982e-005** | **-2.39186** |
| **CDNA FLJ30303 fis, clone BRACE2003269** | **---** | **5.04444e-005** | **-2.29359** | **4.68566e-005** | **-2.30957** | **3.8718e-005** | **-2.35178** |
| **Homo sapiens, Similar to hypothetical protein FLJ20378, clone IMAGE:4179392, mRN** | **---** | **1.71992e-005** | **-2.33174** | **2.82472e-005** | **-2.22978** | **8.02945e-005** | **-2.03961** |
| **nucleotide-binding oligomerization domain containing 2** | **NOD2** | **3.23249e-005** | **-2.36604** | **9.92379e-006** | **-2.66198** | **1.90858e-005** | **-2.49063** |
| **disrupted in renal carcinoma 2** | **DIRC2** | **7.56847e-005** | **-2.38098** | **8.79886e-005** | **-2.3456** | **1.54732e-005** | **-2.8157** |
| **GTPase, IMAP family member 5** | **GIMAP5** | **1.38202e-005** | **-2.39015** | **9.96327e-005** | **-2.01121** | **1.04846e-005** | **-2.45352** |
| **reticulon 4 interacting protein 1** | **RTN4IP1** | **1.55335e-005** | **-2.39486** | **7.9822e-005** | **-2.07027** | **2.15702e-005** | **-2.32269** |
| **chromosome 12 open reading frame 26** | **C12orf26** | **7.17069e-006** | **-2.44958** | **2.15376e-005** | **-2.21713** | **6.24986e-005** | **-2.02785** |
| **OMA1 homolog, zinc metallopeptidase (S. cerevisiae)** | **OMA1** | **5.44079e-005** | **-2.45067** | **9.23327e-005** | **-2.32463** | **3.46174e-005** | **-2.56781** |
| **CDNA FLJ38785 fis, clone LIVER2001329** | **---** | **9.3759e-007** | **-2.50113** | **1.95886e-006** | **-2.34159** | **7.28244e-007** | **-2.56066** |
| **Transcribed locus** | **---** | **3.16463e-005** | **-2.53832** | **1.29188e-005** | **-2.79298** | **6.0226e-006** | **-3.04575** |
| **exonuclease domain containing 1** | **EXOD1** | **1.17144e-005** | **-2.55093** | **6.53997e-005** | **-2.16949** | **2.4304e-005** | **-2.37562** |
| **Transcribed locus, strongly similar to NP_038703.3 spectrin beta 1 [Mus musculus** | **---** | **1.83172e-006** | **-2.55648** | **1.70244e-006** | **-2.5745** | **1.05393e-006** | **-2.69849** |
| **zinc finger protein 64 homolog (mouse)** | **ZFP64** | **2.90682e-006** | **-2.58207** | **2.26597e-005** | **-2.1449** | **1.90131e-006** | **-2.6933** |
| **GTPase, IMAP family member 1** | **GIMAP1** | **1.33551e-006** | **-2.62547** | **1.2611e-006** | **-2.64026** | **1.00347e-006** | **-2.70071** |
| **transcription factor 4** | **TCF4** | **3.50014e-007** | **-2.63559** | **2.03828e-007** | **-2.77895** | **4.05662e-007** | **-2.59884** |
| **tumor necrosis factor, alpha-induced protein 8-like 1** | **TNFAIP8L1** | **1.54257e-005** | **-2.65107** | **6.10875e-005** | **-2.30954** | **2.01335e-005** | **-2.57846** |
| **GTPase, IMAP family member 5** | **GIMAP5** | **1.91106e-006** | **-2.69185** | **8.05723e-006** | **-2.34632** | **2.79146e-006** | **-2.59226** |
| **CDNA FLJ38039 fis, clone CTONG2013934** | **---** | **3.91309e-006** | **-2.73452** | **7.68428e-006** | **-2.5532** | **3.93943e-006** | **-2.73261** |
| **mannosyl (alpha-1,3-)-glycoprotein beta-1,4-N-acetylglucosaminyltransferase, iso** | **MGAT4A** | **1.60131e-005** | **-2.76835** | **7.9663e-005** | **-2.34298** | **1.05294e-005** | **-2.90126** |
| **chromosome 20 open reading frame 19** | **C20orf19** | **3.66585e-006** | **-3.02642** | **1.21e-005** | **-2.65532** | **1.71966e-005** | **-2.56066** |
| **dual adaptor of phosphotyrosine and 3-phosphoinositides** | **DAPP1** | **2.69226e-007** | **-3.06331** | **8.69653e-007** | **-2.70848** | **4.79581e-007** | **-2.87884** |
| **RAB3D, member RAS oncogene family** | **RAB3D** | **8.7819e-007** | **-3.07352** | **3.92713e-006** | **-2.62158** | **1.27294e-006** | **-2.9494** |
| **M-phase phosphoprotein 9** | **MPHOSPH9** | **1.06633e-005** | **-3.07952** | **9.30507e-005** | **-2.42116** | **6.16283e-005** | **-2.52744** |
| **yippee-like 2 (Drosophila)** | **YPEL2** | **4.83384e-006** | **-3.08365** | **2.80372e-005** | **-2.54171** | **6.71927e-006** | **-2.96809** |
| **spectrin repeat containing, nuclear envelope 1** | **SYNE1** | **1.67843e-005** | **-3.21344** | **6.32101e-005** | **-2.73866** | **2.26905e-005** | **-3.09455** |
| **CDNA FLJ45384 fis, clone BRHIP3021987** | **---** | **1.86004e-006** | **-3.48992** | **5.29917e-006** | **-3.07061** | **1.60505e-006** | **-3.5565** |
| **pyruvate dehydrogenase kinase, isozyme 4** | **PDK4** | **2.08539e-006** | **-3.58237** | **5.72871e-006** | **-3.15622** | **2.07233e-006** | **-3.58531** |
| **mannosyl (alpha-1,3-)-glycoprotein beta-1,4-N-acetylglucosaminyltransferase, iso** | **MGAT4A** | **5.86105e-005** | **-3.93799** | **5.69254e-005** | **-3.95597** | **3.75072e-005** | **-4.2265** |
| **Transcribed locus** | **---** | **1.61075e-006** | **-3.95188** | **1.53591e-005** | **-2.9613** | **5.07942e-006** | **-3.3903** |
| **progestin and adipoQ receptor family member VIII** | **PAQR8** | **6.62375e-006** | **-3.95232** | **2.40773e-005** | **-3.30853** | **2.1046e-006** | **-4.70074** |
| **apolipoprotein L, 3** | **APOL3** | **3.26057e-007** | **-3.98953** | **6.63807e-006** | **-2.76969** | **7.12461e-007** | **-3.59772** |
| **chromosome 5 open reading frame 20 /// TIFA-related protein TIFAB** | **C5orf20 /// TIFAB** | **6.55931e-006** | **-4.02275** | **1.97697e-005** | **-3.44529** | **2.89791e-006** | **-4.55145** |
| **MRNA; cDNA DKFZp434N0220 (from clone DKFZp434N0220)** | **---** | **1.52679e-006** | **-4.05143** | **2.61621e-006** | **-3.75923** | **1.26574e-006** | **-4.1616** |
| **GTPase, IMAP family member 2** | **GIMAP2** | **3.65785e-006** | **-4.29345** | **2.67514e-005** | **-3.2499** | **6.98384e-006** | **-3.90368** |
